# Revealing misassembled segments in the bovine reference genome by high resolution linkage disequilibrium scan

**DOI:** 10.1186/s12864-016-3049-8

**Published:** 2016-09-05

**Authors:** Adam T. H. Utsunomiya, Daniel J. A. Santos, Solomon A. Boison, Yuri T. Utsunomiya, Marco Milanesi, Derek M. Bickhart, Paolo Ajmone-Marsan, Johann Sölkner, José F. Garcia, Ricardo da Fonseca, Marcos V. G. B. da Silva

**Affiliations:** 1 Faculdade de Ciências Agrárias e Veterinárias, Universidade Estadual Paulista - UNESP, Campus de Jaboticabal, São Paulo, Brasil; 2 Nofima, Ås, Norway; 3 Faculdade de Medicina Veterinária de Araçatuba, Universidade Estadual Paulista - UNESP, Campus de Araçatuba, São Paulo, Brasil; 4 Animal Genomics and Improvement Laboratory, ARS, USDA, Beltsville, MD USA; 5 Institute of Zootechnics and Biodiversity and Ancient DNA Research Center, Università Cattolica del Sacro Cuore, Piacenza, Italy; 6 Nutrigenomics and Proteomics Research Center - PRONUTRIGEN, Università Cattolica del Sacro Cuore, Piacenza, Italy; 7 Department of Sustainable Agricultural Systems, Division of Livestock Sciences, BOKU - University of Natural Resources and Life Sciences, Vienna, Austria; 8 International Atomic Energy Agency (IAEA) Collaborating Centre on Animal Genomics and Bioinformatics, Araçatuba, São Paulo, Brasil; 9 Faculdade de Ciências Agrárias e Tecnológicas, Universidade Estadual Paulista - UNESP, Campus de Dracena, São Paulo, Brasil; 10 Embrapa Gado de Leite, Juiz de Fora, Minas Gerais Brasil

**Keywords:** Misassembly, Linkage disequilibrium, GWAS, Imputation, *Bos taurus*, *Bos indicus*

## Abstract

**Background:**

Misassembly signatures, created by shuffling the order of sequences while assembling a genome, can be detected by the unexpected behavior of marker linkage disequilibrium (LD) decay. We developed a heuristic process to identify misassembly signatures, applied it to the bovine reference genome assembly (UMDv3.1) and presented the consequences of misassemblies in two case studies.

**Results:**

We identified 2,906 single nucleotide polymorphism (SNP) markers presenting unexpected LD decay behavior in 626 putative misassembled contigs, which comprised less than 1 % of the whole genome. Although this represents a small fraction of the reference sequence, these poorly assembled segments can lead to severe implications to local genome context. For instance, we showed that one of the misassembled regions mapped to the *POLL* locus, which affected the annotation of positional candidate genes in a GWAS case study for polledness in Nellore (*Bos indicus* beef cattle). Additionally, we found that poorly performing markers in imputation mapped to putative misassembled regions, and that correction of marker positions based on LD was capable to recover imputation accuracy.

**Conclusions:**

This heuristic approach can be useful to cross validate reference assemblies and to filter out markers located at low confidence genomic regions before conducting downstream analyses.

**Electronic supplementary material:**

The online version of this article (doi:10.1186/s12864-016-3049-8) contains supplementary material, which is available to authorized users.

## Background

Studies using next generation sequencing or single nucleotide polymorphism’s (SNP) array data often rely on genomic coordinates from reference assemblies. However, as stated by Salzberg and York [1]: “Certainly, there might be errors at some small rate in genome sequence data generation and assembling”. These errors frequently affect analyses based on genomic data, such as genotype phasing, imputation and *post hoc* annotation in genome-wide association studies (GWAS). Therefore, systematic identification and correction of misassembled segments in a reference genome improves assembly quality locally, increases the power of analyses, decreases the rate of false positive results and ultimately permits researchers to formulate hypotheses on the basis of more correct findings.

Methods relying on sequence [2, 3], *in situ* hybridization [4] or optical mapping [3] data have been used to pinpoint assembly problems in the bovine reference genome. However, as linkage disequilibrium (LD) decays as a function of distance in a chromosome, the recent availability of a large number of bovine samples genotyped by high-density single nucleotide polymorphism (SNP) arrays offers the opportunity to exploit LD information to correct assembly mistakes. By assessing marker LD, one can identify segments in the reference genome which do not belong to their assigned locations, as well as estimate their most likely true position.

Evidence of misassembly signatures in the bovine UMDv3.1 reference genome have been recently reported. Bohmanova et al. [5], characterizing LD in North American Holstein, identified 223 SNPs (0.57 % of the SNPs in the Illumina® BovineSNP50 BeadChip, Bovine50k hereafter) producing unexpected long distance LD. Using a higher density SNP array (Illumina® BovineHD BeadChip, BovineHD hereafter) to test imputation in Fleckvieh (*Bos taurus*), Pausch et al. [6] identified 5,039 out of 599,535 SNPs (0.89 %) exhibiting poor imputation performance. Poor imputation accuracies of neighboring SNPs were also reported in *Bos indicus*, namely Nellore beef cattle [7] and Gyr dairy cattle [8]. Since the imputation process depends on LD blocks to infer missing marker genotypes, an assembly error would be the main reason for poor genotype imputation [9].

Here, we report a heuristic approach based on LD analysis to identify misassembly signatures and estimate an approximate re-location for the missassembled segments in the UMDv3.1 bovine reference genome. Furthermore, we demonstrate the effect of detecting and correcting assembly errors in two case studies: i) GWAS on the Polled/Horned phenotype in Nellore cattle and ii) imputation in Nellore and Italian Holstein. Misassembled segments directly affected GWAS results and imputation performance. Re-location of misassembled segments provided new insights on the so far unsolved nature of the locus controlling horn development in cattle and substantially improved imputation accuracy locally.

## Results

### Genotypes and data filtering

To minimize spurious LD patterns caused by the confounding effect of breed-specific gametic-phase disequilibrium and structural variants or epistatic loci under selection, we selected genotypes from two genetically divergent populations, one of *B. taurus* (Holstein) and one of *B. indicus* (Nellore) with distinct production purposes (i.e., dairy and beef), to identify unexpected LD patterns in common regions that are likely due to genome misassembly. These breeds were chosen because the largest bulk of re-sequencing data driving the design of the high density cattle SNP panel (BovineHD) were from Holstein (HOL), and most of the *B. indicus* specific SNPs were derived from a draft genome assembly of a Nellore (NEL) bull. Nellore genotypes were provided by the Zebu Genome Consortium (ZGC) and Italian Holstein genotypes were provided by the INNOVAGEN project - “Research and innovation in italian animal breeding II”.

After data filtering, 886 bulls and 564,865 autosomal SNPs and 811 bulls and 485,455 autosomal SNPs were used for estimating marker-pair LD values in HOL and NEL, respectively. Combination of the two SNP lists resulted in 675,859 markers, which covered about 2.5 Gb of the UMDv3.1 reference genome, with an average intermarker distance of 3.71 ± 5.21 kb. Although highly important, sexual chromosomes were excluded from this analysis due to their particularly complex model of inheritance. Moreover, there is not an assembly for the Y chromosome, the pseudo-autosomal region of the X chromosome is not well characterized, and the use of male data only may bias LD estimates.

### Detection of misassemblies

**Fig. 1:**
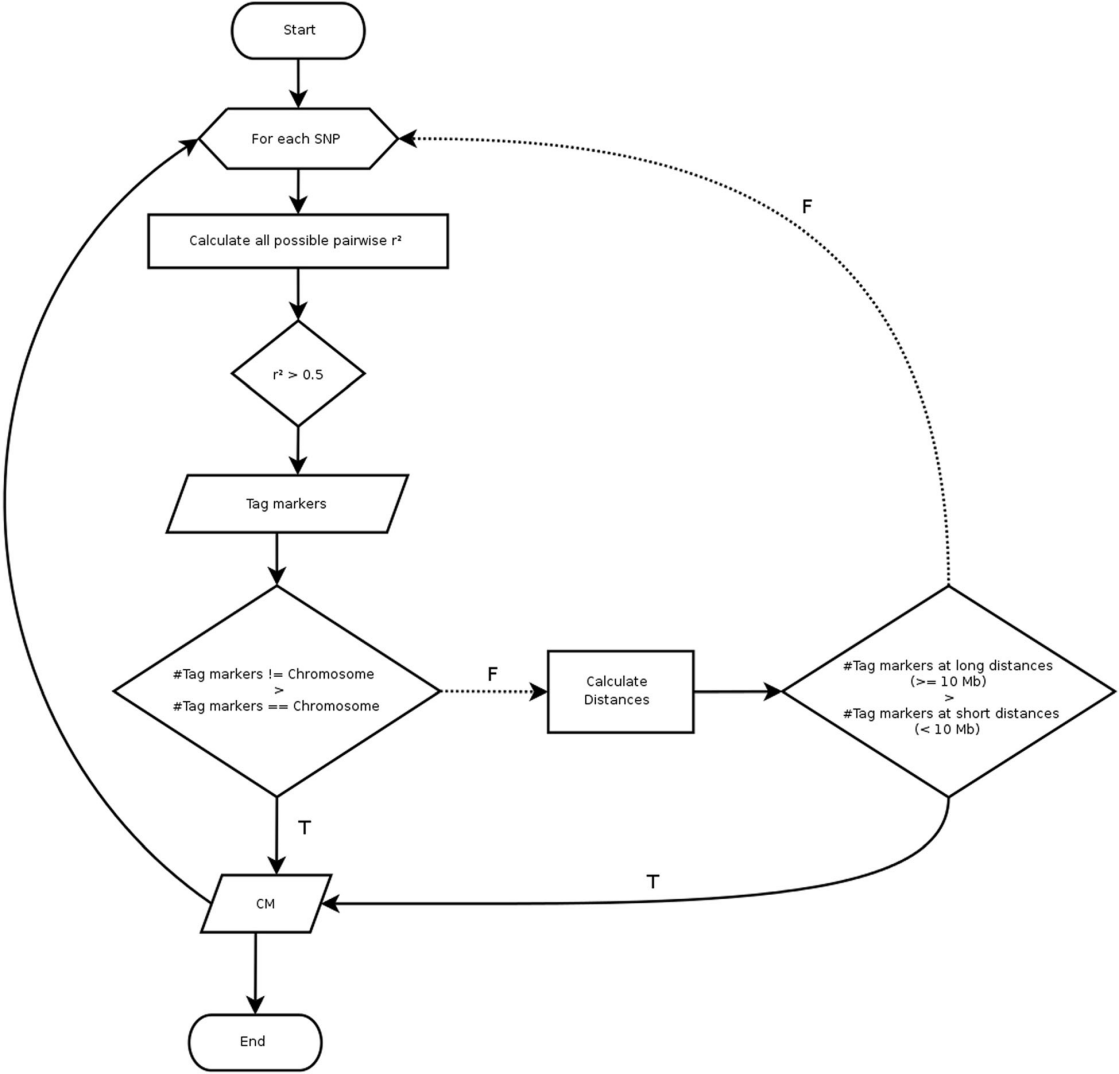
Flowchart of the pipeline to detect Candidate Misplaced Markers (CMM). Oval symbols denote the beginning and end of the pipeline; the hexagon indicates the beginning of a loop structure; rectangles indicate a computation; diamonds indicate the points where decisions are made; parallelograms indicate output of information. The arrows indicate the flow of the pipeline and F and T denote FALSE and TRUE, respectively

**Fig. 2:**
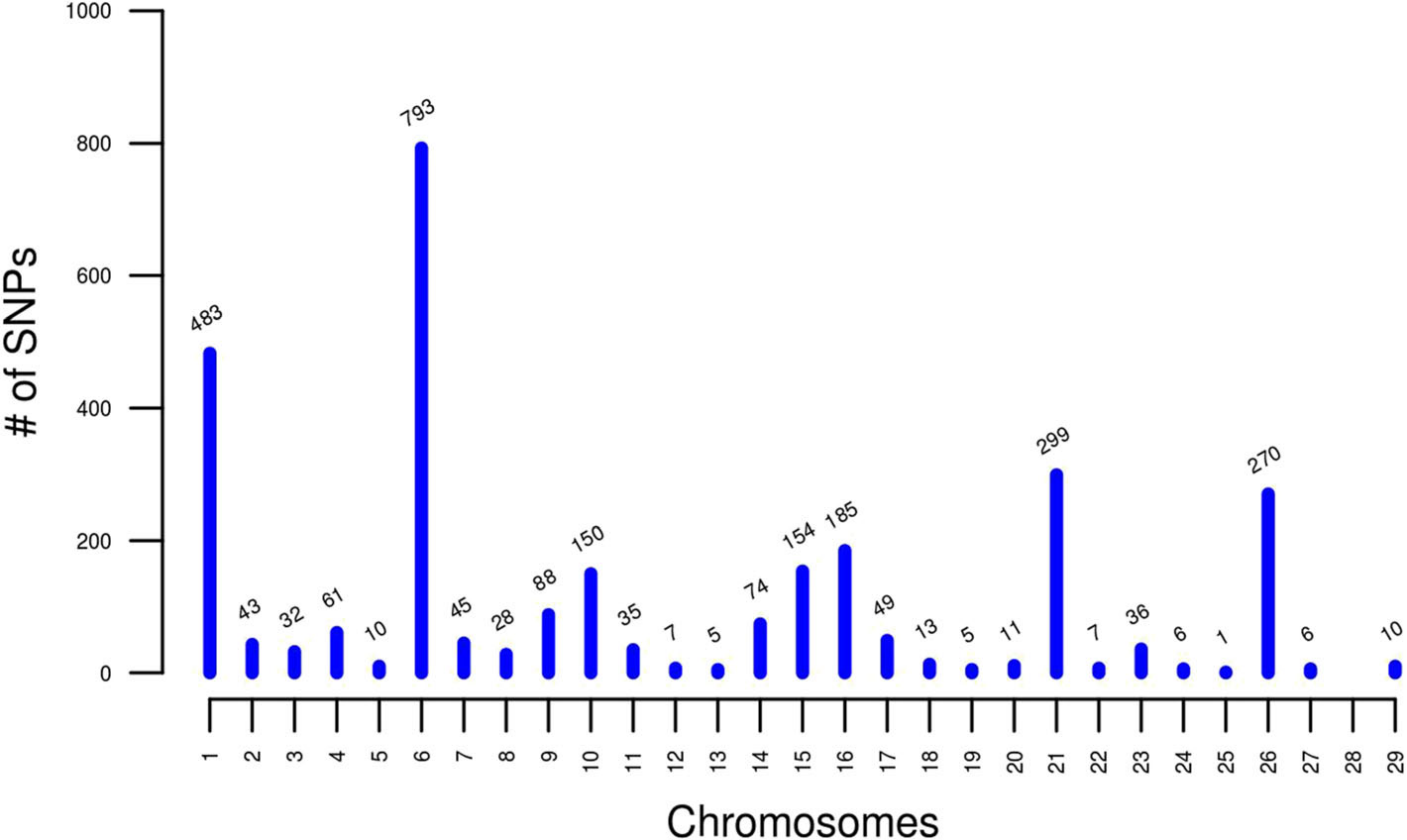
Histogram of the number of SNPs with unexpected Linkage Disequilibrium (LD) pattern detected by chromosome

We applied our pipeline (Fig. 1) to the NEL and HOL datasets, and detected 2,906 Candidate Misplaced Markers (CMM) having unexpected LD patterns. Among these, 1,597 were found in both breeds, 808 only in HOL, and 501 only in NEL. A total of 796 (27.29 %), 484 (16.62 %), 299 (10.29 %) and 270 (9.29 %) CMMs mapped on chromosomes 6, 1, 21 and 26, respectively. These four chromosomes contained 63.49 % of SNPs with unexpected LD behavior likely carrying the largest assembly errors in the UMDv3.1 reference. We found no association between chromosome length and the number of CMMs (Fig. 2). Interestingly, no misplacement was found on chromosome 28, which indicates a highly accurate assembly of this chromosome.

We mapped the 2,906 CMMs against their contigs of origin and identified 626 Candidate Misassembled Contigs (CMCs). A graphical inspection of the LD decay of the detected CMCs allowed us to recognize three types of signatures of misassembly: i. Misplaced SNP (MisSNP); ii. Misassembled Segment (MisSeg); and iii. Partially Misassembled Contig (PMisCon). Briefly, MisSNPs are defined as single markers showing strong LD with SNPs in a segment far apart (>10 Mb) in the same or in a different chromosome and no evidence of LD with SNPs surrounding it. MisSeg comprises one or more CMCs showing markers with strong LD with SNPs within the segment and with SNPs far apart in the same or in a different chromosome, but no evidence of LD with SNPs in adjacent contigs. Finally, a PMisCon is a special case of CMC with markers having a mixture of behaviors. A fraction of them had expected LD values with nearby markers in the region, while others show no evidence of LD at their sites but strong LD with another segment elsewhere in the genome. Examples of MisSNP, MisSeg and PMisCon are shown in Additional file 1: Figure S1, Additional file 2: Figure S2 and Additional file 3: Figure S3, respectively.

From the total number of detected markers, 2,796 (96.21 %) were included in MisSeg, 76 (2.62 %) in PMisCon and 34 (1.17 %) in MisSNP. The complete list of the detected signatures of misassembly, as well as all the graphs built for the MisSNP, MisSeg and PMisCon cases, can be seen in Additional file 4: Table S1 and Additional file 5: Figure S4, Additional file 6: Figure S5 and Additional file 7: Figure S6, respectively. Hereafter, we focus on the MisSeg cases as they comprised the vast majority of the signatures of misassembly. After grouping adjacent CMCs, we were able to identify 246 MisSeg, from which 180 were detected in both breeds, 32 only in HOL and 34 only in NEL. The breed-specific cases represented differences in local coverage by polymorphic SNPs between breeds, since markers with low minor allele frequency (MAF) were removed in the quality control. The largest MisSeg was found in both breeds on chromosome 21, and consisted of 35 adjacent contigs (of which 17 were gaps and 18 were sequence contigs) covering a ~1.08 Mb segment sheltering 297 SNPs (of which 237 were detected in HOL and 164 in NEL). The smallest segment was found on chromosome 5, consisting of a single contig of 679 bp covered by only one marker (detected only in NEL).

**Fig. 3:**
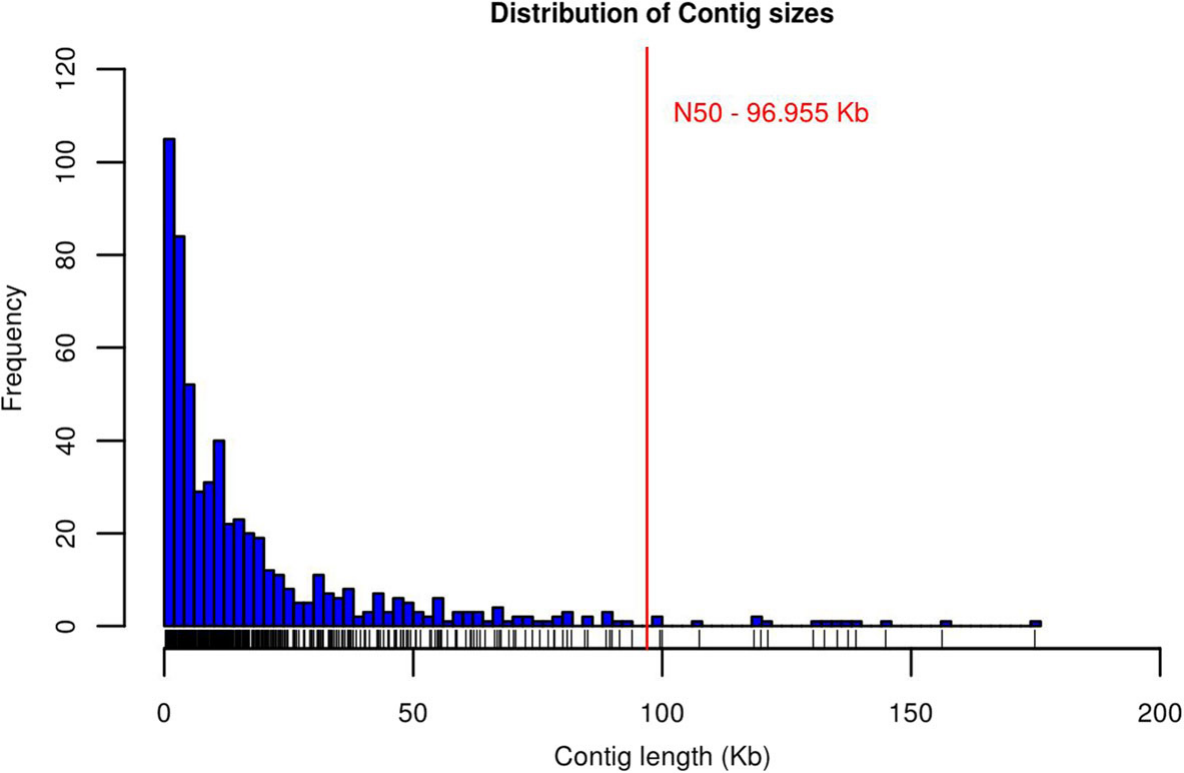
Distribution of frequencies of misassembled contig sizes. The red line represents the N50 of the UMDv3.1 bovine genome assembly

The average size of CMCs included in MisSeg was 18.88 ± 26.02 kb. The vast majority of the CMCs (97,6 %) had length smaller than the N50 in UMD v3.1 (96.955 Kb). Only 2.4 % of the CMCs had length greater than the N50 (Fig. 3). This suggests that smaller contigs are more prone to assembly errors, leading to scaffolding artifacts that can be detected in the form of MisSeg. Although rare, CMCs large than the N50 were also found, which may represent brute force assembly of low quality and low complexity sequences [1].

Considering the percentage of the reference genome covered by MisSeg, we found that less than 1 % of the reference sequence was wrongly assembled, which confirms its robustness. At the chromosome level, we found error rates of 0.179 % for chromosome 6, 0.11 % for chromosome 1, 0.095 % for chromosome 16 and 0.056 % for chromosome 26.

### Estimation of true locations

Approximate locations were estimated for the detected misassembled segments using r^2^ information. Note that this process can only guide the rearrangements of CMCs to their estimated true location, and that the *de facto* correction of these assembly errors demand consorting the LD analysis with re-alignment and re-assembly of raw reads against the targeted locations. Also, due to differences in the extent of LD and allele frequencies between breeds, the locations suggested by LD analysis varied between NEL and HOL. In fact, differences in LD-based estimates in NEL and HOL ranged from 0 to 26 Mb, with an average of 871 kb.

**Fig. 4:**
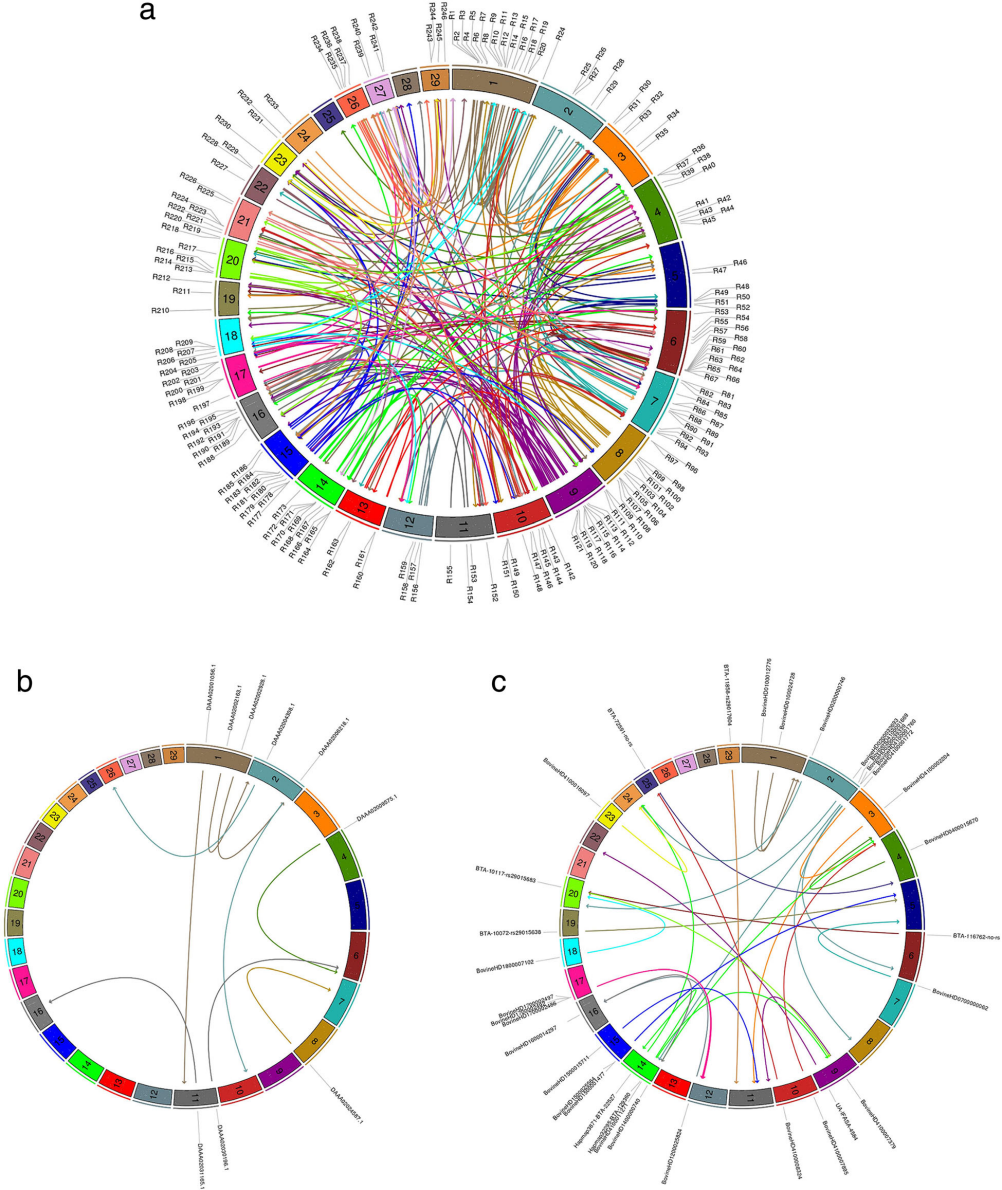
Circos plot of misassemblies. The 29 bovine autosomes are represented by polygons of different colors. Arrows within the inner track indicate the flow of misassemblies from their current to their estimated true locations (*arrow heads*). **a** Annotations in the outer track refer to the SNP probe name in Additional file 4: Table S1. **b** Annotations in the outer track refer to the MisSeg aliases in Additional file 4: Table S1. **c** Annotations in the outer track refer to the contig name in Additional file 4: Table S1

Estimated locations for MisSNP, MisSeg and PMisCon are graphically represented in Fig. 4c, a and b, respectively (a full description of all estimated locations can be found in Additional file 4: Table S1). Interestingly, although some chromosomes presented few or no assembly errors in our LD analysis, all chromosomes acted as CMC receptors.

### Genome-wide scan for absence of horns in Nellore cattle

**Fig. 5:**
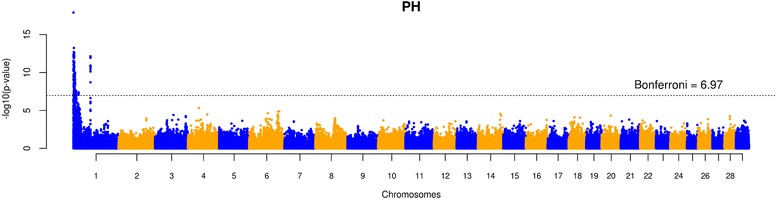
Manhattan plot of -log10(p-value) of SNPs for the Polled/Horned (PH) phenotypes in Nellore cattle

We found 295 markers significantly associated (*p* < 1.07 × 10^−7^) with absence of horns in NEL. These markers were divided into two different signals on chromosome 1 (Fig. 5). The most significant SNP in the first signal (*p* = 1.23 × 10^−18^) was located at 78.66 kb, nearby the widely known *POLL* locus [10]. The most significant SNP in the second signal (*p* = 7.41 × 10^−13^) was located at 60.66 Mb, approximately 60.58 Mb apart from the most significant SNP in the first signal.

We analyzed the LD patterns in the two regions independently to define the limits of the associated loci. Next, we analyzed the LD between the two putative candidate regions and found that the most significant SNPs in the two signals are in very strong LD (*r*^*2*^ = 0.88). In fact, the average r^2^ among all significant SNPs was 0.73 ± 0.18. Looking carefully at the behavior of LD decay in the second signal, we noted strong LD among markers within the region but no evidence of LD with surrounding markers. Indeed, the SNPs pertaining to the second signal are located in the R6 MisSeg (1:60,578,448–60,664,293 bp) identified in our LD-based analysis of assembly errors (Additional file 4: Table S1 and Additional file 6: Figure S5).

### Imputation in Italian Holstein and Nellore

We evaluated the impact of markers in MisSNP, MisSeg or PMisCon on the global and local performance of imputation from medium (Bovine50k) to high density (BovineHD) chips.

The average genome-wide SNP imputation accuracy was 0.9695 ± 0.0271 in NEL and 0.9933 ± 0.0161 in HOL. After excluding all putative assembly problems, accuracies increased marginally to 0.9717 ± 0.0231 in NEL and 0.9937 ± 0.0094 in HOL. After correcting the positions, accuracies also increased marginally to 0.9740 ± 0.0231 in NEL and 0.9944 ± 0.0127 in HOL. However, the local imputation performance before and after correcting SNP locations were 0.7868 ± 0.1971 and 0.9725 ± 0.0386 in NEL and 0.8359 ± 0.1989 and 0.9909 ± 0.0520 in HOL, respectively.

**Fig. 6:**
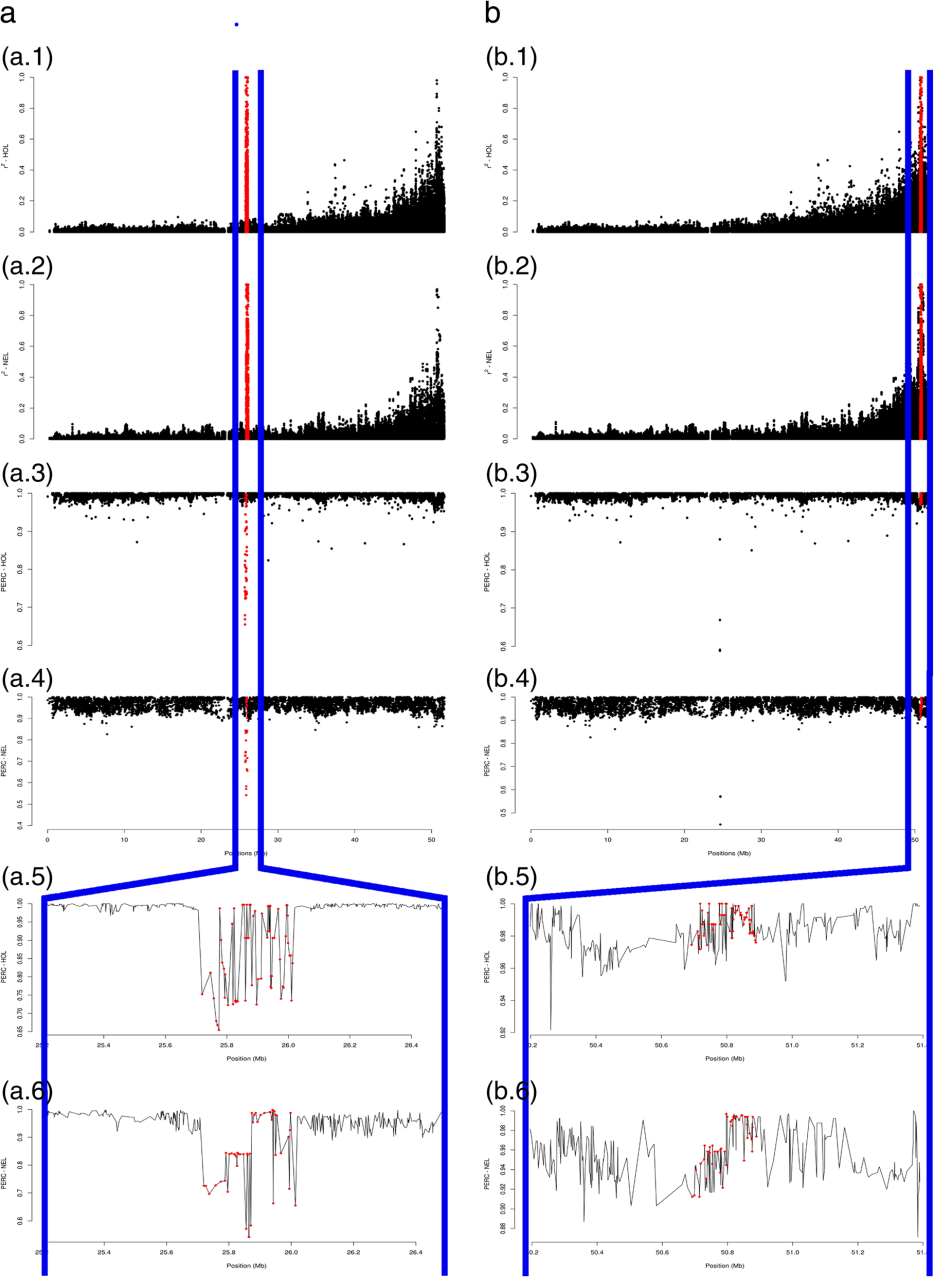
Linkage disequilibrium (LD) decay and SNP imputation accuracies for the R237 segment in Italian Holstein (HOL) and Nellore (NEL). From the top to the bottom of the picture, before (**a**) and after (**b**) correcting the segment using LD information, we plotted the LD decay of the segment in Italian Holstein (a.1, b.1) and Nellore (a.2, b.2), the SNP imputation accuracies in Italian Holstein (a.3, b.3) and Nellore (a.4, b.4), and a magnification of the segment in Italian Holstein (a.5, b.5) and Nellore (a.6, b.6)

In order to illustrate the local effect of correcting assembly errors, we showed the local genotype prediction accuracy before and after correcting the location of the R237 MisSeg (26:25,715,286–26,015,674 bp). Considering the originally assigned location of R237, we obtained average accuracies of 0.8452 ± 0.1339 in NEL and 0.8612 ± 0.1044 in HOL. After correction, SNPs in R237 increased their imputation accuracies considerably in both NEL and HOL, with averages of 0.9616 ± 0.0282 and 0.9866 ± 0.0096, respectively (Fig. 6).

## Discussion

Assembly errors usually arise from nucleotide repeat collapse and expansion; as well as sequence rearrangements and inversions [11]. These two types of errors are mostly caused by repetitive DNA, which increases the complexity of sequence assembly using short reads and imposes great computational challenges in contig formation [12]. These assembly errors are often silent in the sense that without additional data from long reads or population-level genotypes [11] they remain unidentified.

Evidences of assembly problems in the bovine reference have been recently published in LD and imputation studies. For instance, Bohmanova et al. [5] assessed the extent of LD in American Holstein using a medium density SNP panel, namely Illumina® BovineSNP50, and detected 223 SNPs with unexpected LD decay. Milanesi et al. [9], comparing imputation accuracies in Simmental cattle using different reference genome assemblies, found segments poorly imputed in one reference assembly that in the other mapped to a different chromosome with low imputation error rates, arguing that a cluster of consecutive markers with a high percentage of imputation errors may be evidence of misassembled segments in the reference genome. Also, studying strategies for genotype imputation in Gyr (*B. indicus*) dairy cattle, Boison and colleagues [8] found poor imputation accuracies for certain segments of the genome. One of the segments ranged from 44.8 to 45.3 Mb on chromosome 1. The same segment was reported by Carvalheiro et al. [7] in Nellore cattle, and by Pausch et al. [6] in Fleckvieh (*B. taurus*) dairy cattle. In a more detailed analysis we could break this segment down to two segments, ranging from 44.8 to 45.13 Mb and from 45.14 to 45.2 Mb. Overall, these studies reported the unusual LD and imputation behavior of SNP markers as an incidental finding arising as a side product of their main targeted analyses and avoided a systematic mapping and interpretation of the errors, as well as an assessment of their potential impact on coordinates-dependent analyses.

Here, we were able to pinpoint putative assembly errors in the UMDv3.1 bovine reference genome by using population-level LD data generated from high-density SNP genotypes of two divergent breeds of cattle. Our study revealed that, at least to the extent our LD analysis could detect, a small fraction (<1 %) of the reference genome is inadequately assembled. Also, over 97.6 % of missassembled segments were found to be smaller than the N50 in UMDv3.1, reflecting that these errors largely represent low confidence small contigs. Although the detected missassembled segments may not threat the global quality of the UMDv3.1 reference, locally these errors are deemed to affect applications that assume correct marker positions.

Studies that are dependent on the positions of molecular markers, such as assessment of the extent of the linkage disequilibrium, imputation, synteny, genome wide association studies, detection of structural variants, runs of homozygosity, among others, can be greatly affected by local assembly errors. For instance, Fadista & Bendixen [13] sought discrepancies of SNP coordinates in commercial human arrays, and found that one significant marker in a GWAS performed by Macgregor et al. [14] had its position misplaced in the reference genome. Gene annotations for such a marker could lead to a wrong interpretation of a biological pathway and give rise to misleading hypotheses.

To illustrate the impact of assembly errors in GWAS, we use the example of a genome-wide scan for absence of horns in Nellore cattle (Fig. 5). The scan revealed two significant signals separated by over 60 Mb on chromosome 1. Without the proper care, one could assume that the two peaks are independent and proceed with functional annotation. The first peak maps to the well known *POLL* locus in *B. taurus*, which shelters some candidate genes for the trait, such as *OLIG2*, *FOXL2* and *RXFP2* [15]. The nearest gene to the second peak is *GAP43* (growth associated protein 43), located approximately 200 kb downstream of the marker. The gene is involved in innervation and development of long bones [16, 17]. Recently, a histological study of polled and horned bovine fetuses presented evidence of thick nerve fibers in the dermis underlying the horn bud in both polled and wild type animals [18], suggesting the horn is a highly sensitive area. This finding opens a window to speculations about the participation of innervation in the horn development. Given this set of evidences, the logics could suggest a role of *GAP43* in horn development.

In spite of the appealing functional candidacy of *GAP43*, the positional association is clearly wrong. The contig containing the significant marker was estimated to be located within the *POLL* locus, and the region surrounding *GAP43* showed no evidence of assembly errors. Moreover, the presence of portions of the *POLL* locus assembled somewhere else in the genome could be an important contributor to the ongoing dispute regarding the candidate gene and causal variant underlying the trait.

We also carried out an imputation study to assess the impact of misassembled segments in genotype prediction performance in Nellore and Holstein. As expected, the impact of assembly errors on the average imputation accuracy was negligible (0.22 % in Nellore and 0.04 % in Holstein). However, the correction of the positions of the markers using LD increased the local imputation accuracy substantially (differences of 18.57 % in Nellore and 15.5 % in Holstein). These findings show that the use of LD to estimate the correct position of misassembled segments may help to improve the local quality of the assembly, as well as to increase confidence in association, phasing and imputation analyses.

Although very informative, the heuristic approach adopted here has important limitations that are intrinsic to the LD analysis. Ideally, one would require the candidate misassembled contigs to be detected in several breeds independently as a strategy to increase the likelihood that the observed pattern was caused by a problem in the reference assembly, rather than breed-specific linkage due to structural variants or epistatic loci under selection or gametic phase disequilibrium. However, due to heterogeneous marker coverage in different breeds, some signals of assembly problems could remain undetected in some breeds due to the effect of ascertainment bias, and in this case requiring the signal to appear in at least one breed could give rise to false positives.

Khatkar et al. [19] reported a 99.9 % empirical assignment accuracy when markers had their positions predicted by pair-wise LD information. However, in the present study, we found an average difference of 871 kb when locations were estimated based on LD in Italian Holstein or Nellore. This suggests that the extent of LD and allele frequencies in the empirical genotype data can greatly affect the estimates of true locations of misassembled segments. Of note, the estimates provided here are rough approximations, and the parsimony of corrections based solely on LD information must be cross-validated by complementary analyses, such as increase in local imputation accuracies, or reinforced by additional sequence data.

Finally, although some chromosomes exhibited no or few cases of assembly problems, all chromosomes acted as receptors of misassembled segments. This indicates that our estimate of percentage of errors is likely to be underestimated, as some types of assembly errors may not produce a detectable LD signature.

## Conclusions

Assembly errors in the UMDv3.1 bovine reference genome were pinpointed by SNP LD analysis. We showed that the majority of the errors comprised small contigs that were either malformed or placed wrongly in the genome. Although the incidence of errors was low, confirming the robustness of the bovine reference sequence assembly UMDv3.1, the misassembled segments were shown to largely impact local imputation performance and the interpretation of GWAS results. Estimation of the correct location of the misassembled segments significantly improved imputation accuracy locally and excluded a functional candidate gene as a putative determinant of horn development in polled and horned Nellore cattle. Our heuristic approach can be useful in refining draft assemblies already available and as a strategy to filter out markers that could largely affect interpretation of downstream analysis.

## Methods

### Ethics statement

This study involved no animal experimentation and was exempt from local ethics committee evaluation because DNA was extracted from commercialized semen straws.

### Genotypes and data filtering

A total of 1,009 Italian Holstein (Dairy - *Bos taurus*) and 995 Brazilian Nellore (Beef - *Bos indicus*) bulls were genotyped using the BovineHD assay, according to the manufacturer’s protocol. Only autosomal markers with unique genomic coordinates presenting call rate of at least 95 %, minor allele frequency (MAF) greater than 3 % and Fisher’s exact test for Hardy-Weinberg equilibrium greater than 1 × 10^−8^ were considered for LD analyses. Bulls presenting call rate lower than 90 % were excluded. Supplementally, the largest sample set with the least relatedness (<0.4) was optimized using the --rel-cutoff algorithm in PLINK v1.9 [20, 21].

### Detection of markers producing unexpected LD

Linkage disequilibrium between SNPs was measured by the squared Pearson’s product-moment correlation (r^2^) of the genotype vectors (coded as 0, 1 or 2 reference alleles), as implemented in PLINK v1.9 [21]. The algorithm for detecting markers with unexpectedly high LD was divided into a series of steps. First, for each focal SNP, we computed all pairwise squared correlations with the remaining markers in the panel, regardless of their originally assigned genomic location. Second, we filtered in tag markers presenting high LD (*r*^*2*^ > 0.5) with the focal SNP. Third, a table of tag marker counts per chromosome was created. If the largest tag marker count (i.e >50 % of the tag markers) was found in a different chromosome than the one originally assigned for the focal SNP, independently of the tag markers positions, we labeled the focal SNP as a candidate misplaced marker (CMM). Otherwise, if the previous condition failed, we computed the base pair distances between the focal SNP and the tag markers, now on the same chromosome, and classified these distances as long (≥ 10 Mb) or short (<10 Mb). Then, we built a table of tag marker counts per distance class. If the number of correlations at long distances were greater than at short distances, the focal SNP was also labeled as CMM. A scheme of this procedure can be seen in Fig. 1.

The criteria of *r*^*2*^ > 0.5 was empirically defined, based on the low likelihood to find high LD between markers in a different chromosome or far apart on the same chromosome. Nevertheless, LD rapidly decays over up to 5 Mb [5], then the distance of 10 Mb was choose to ensure that SNPs far apart from each other should present low and stable levels of LD. Whether it is not the case, a SNP may be misplaced or located in a misassembled segment of the genome. By using the largest tag marker count (>50 %) for selecting CMM we were extremely selective, once the vast majority of high r^2^ values are expected to be found between SNPs closer to each other, as can be seen in Additional file 8.

As the procedure described above is computationally intensive, additional information about computational performance can be seen in Additional file 9.

### Assessment of signatures of misassembly

Considering the contig as the smallest unit in the reference assembly, candidate misassembled contigs (CMC) were defined as any contig presenting one or more candidate misplaced markers, as detected in the procedure described before. In order to gain insights on the possible sources of errors leading to the misplacement or malformation of contigs, we visually inspected the intra- and inter-chromosomal patterns of LD decay of every single detected CMC. For that matter, we plotted pairwise r^2^ values for all markers within the CMC against all markers on the same chromosome. For the cases where the CMM investigation suggested a different chromosome for the CMC, we plotted r^2^ for both the originally assigned and the new target chromosome. The boundaries of adjacent contigs were merged with the CMC to form a candidate misassembled segment whenever the unexpected LD pattern extended to contigs flanking the CMC.

### Estimation of correct locations for misassembled segments

The unexpected patterns of LD decay were also carefully examined in order to estimate the correct locations of CMC. Estimates for the 5′ and 3′ coordinates were based on the positions of the tag markers presenting the highest r^2^ with the most proximal and distal SNPs within the CMC, respectively. These estimates were taken in both breeds independently, since the allele frequency spectra and consequently the distribution of the markers covering the region could differ between NEL and HOL. Finally, we evaluated the concordance of the estimated true position by the difference of the estimated positions between breeds.

### GWAS for absence of horns in Nellore cattle

Presence or absence of horns was scored by majority voting of image analysis performed by five observers in a subset of 481 Nellore bulls. Phenotype-genotype associations were tested using single-marker regression under a mixed model framework [22], as implemented in the mmscore procedure in GenABEL v1.8-0 [23]. Markers were prioritized for investigation based on a Bonferroni-corrected significance level of α < 0.05.

### Imputation in Nellore and Italian Holstein

We evaluated the impact of excluding and correcting positions of SNP markers in misassembled segments on the global performance of imputation from medium (Bovine50k) to high density (BovineHD) chips by calculating the SNP imputation accuracy as the percentage of correctly imputed genotypes (PERC). We also evaluated the local performance of imputation after correcting the positions of misassembled segments. In order to illustrate that, we examined one of the misassembled segments, located on chromosome 26:25,715,286–26,015,674, before and after correcting the SNP locations by imputing SNPs from a medium (Bovine50k) to high density (BovineHD) panel. To correct the SNP locations we estimated the true location of the segment and then sample the new SNP positions in the segment from an uniform distribution.

The analyses were performed in both breeds dividing the animals in reference and imputation sets in a 5-fold cross-validation scheme. Each group was used as imputation set once. SNPs corresponding to Bovine50k were subset from the BovineHD panel. Imputations were performed using Fimpute [24].

## Additional files

Additional file 1: Figure S1. Example of a Misplaced SNP (MisSNP). It shows the (un)expected linkage disequilibrium between markers when misplaced in the reference genome (PDF 474 kb) Additional file 2: Figure S2. Example of a Misassembled Segment (MisSeg). It shows the (un)expected linkage disequilibrium between markers in an specific segment of the genome when wrongly assembled (PDF 295 kb) Additional file 3: Figure S3. Example of Partially Misassembled Contig (PmisCon). It shows the (un)expected linkage disequilibrium between markers in a partially misassembled contig (PDF 465 kb) Additional file 4: Table S1. Full description of signatures of misassemblies and their estimated true location (XLSX 35 kb) Additional file 5: Figure S4. Misplaced SNPs. It presents all cases of misplaced SNPs found in this study (PDF 3034 kb) Additional file 6: Figure S5. Misassembled segment (MisSeg). It presents all cases of misassembled segments of the reference genome found in this study (PDF 4176 kb) Additional file 7: Figure S6. Partially Misassembled Contig (PmisCon). It presents all cases of partially misassembled contigs of the reference genome found in this study (PDF 7832 kb) Additional file 8: Tag marker count criteria. Describes the empirical criteria used for detecting candidate misplaced markers (PDF 229 kb) Additional file 9: Benchmarking of the r^2^ calculations. Describes the computational performance for calculating all pair-wise r^2^ (PDF 301 kb)

## Abbreviations

Bovine50k: Illumina® BovineSNP50 BeadChip
BovineHD: Illumina® BovineHD BeadChip
CMC: Candidate misplace contig
CMM: Candidate misplaced marker
Gb: Giga bases
GWAS: Genome-Wide Association Study
HOL: Holsteins
LD: Linkage disequilibrium
MAF: Minor allele frequency
Mb: Mega bases
MisSeg: Misplaced segment
MisSNP: Misplaced SNP
NEL: Nellore
PERC: Percentage of correctly imputed genotypes
PH: Polled/Horned
PmisCon: Partially misplaced contig
SNP: Single nucleotide polymorphism
UMDv3.1: Bovine genome assembly produced by University of Maryland
ZGC: Zebu Genome Consortium
