# Supplementary material for: Revealing misassembled segments in the bovine reference genome by high resolution linkage disequilibrium scan
Source: BMC Genomics. 2016 Sep 5;17(1):705. doi: 10.1186/s12864-016-3049-8 (PMC5011828; doi:10.1186/s12864-016-3049-8)

Supplementary Figure S1 - Example of a Misplaced SNP (MisSNP). (a) LD values between markers in the yellow segment (86.8 - 86.9 Mb) and all other markers are plotted along chromosome 1. The yellow segment has unexpected high correlation with a region far apart on the same chromosome 1 (147 - 151 Mb, green segment); (b) A close inspection of the segments (yellow and green) reveal a single marker (BovineHD0100024728 in red) at 86,849,191 bp, responsible for the unexpected LD; (c) LD values are plotted between the misplaced marker and all other markers along the chromosome. The dot at $r^2 = 1$ in correspondence of the misplaced marker indicates the correlation of the marker with itself; (d) LD between markers in the yellow segment and all other markers are plotted along chromosome 1, after the exclusion of the misplaced SNP. The LD pattern suggests that the marker mapped in the wrong location should be moved to around 147 Mb; (e) The re-location of the misplaced marker to position 147 Mb yields the expected LD decay pattern of markers located in the green segment.

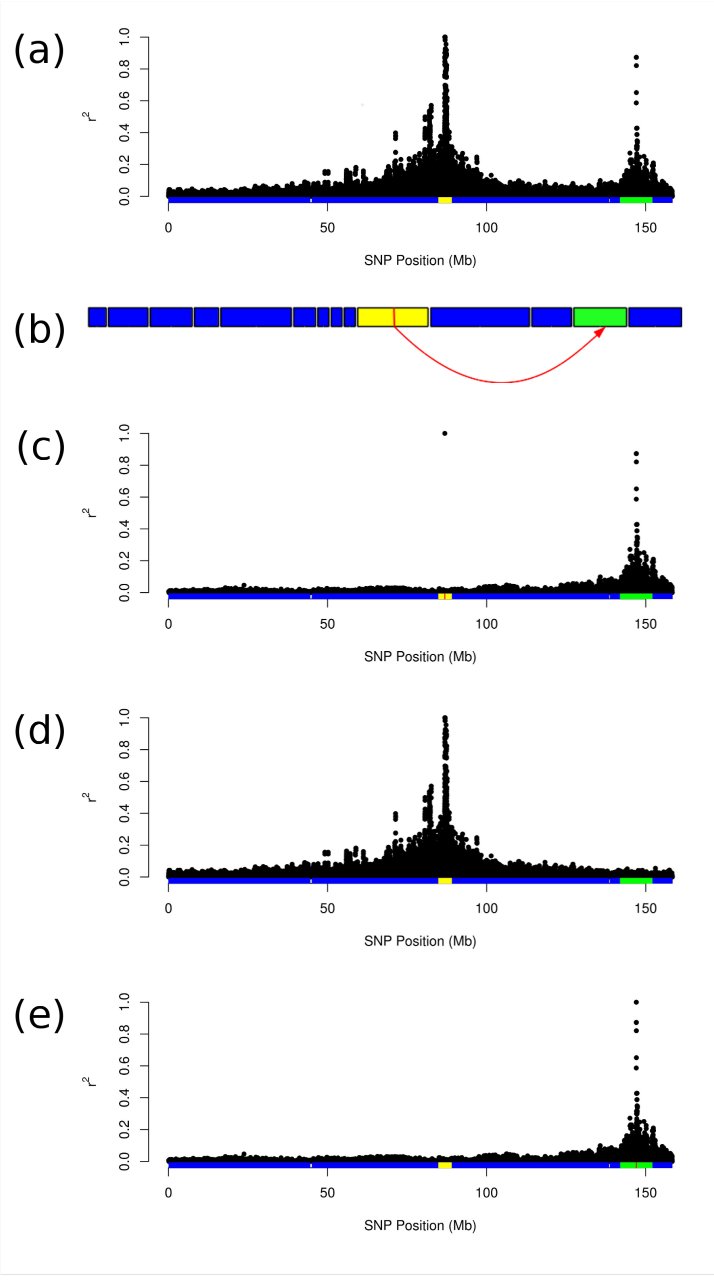

Supplement: Additional file 1: Figure S1. — Example of a Misplaced SNP (MisSNP). It shows the (un)expected linkage disequilibrium between markers when misplaced in the reference genome (PDF 474 kb) [file 12864_2016_3049_MOESM1_ESM.pdf]
