# Supplementary material for: Revealing misassembled segments in the bovine reference genome by high resolution linkage disequilibrium scan
Source: BMC Genomics. 2016 Sep 5;17(1):705. doi: 10.1186/s12864-016-3049-8 (PMC5011828; doi:10.1186/s12864-016-3049-8)

Supplementary Figure S2 - Example of a Misassembled Segment (MisSeg). (a) A segment (see R237 in Supplementary File Figure S5 online) assigned to chromosome 26 position 25.7 - 26 Mb (in red) has unexpected high LD with SNPs at positions 50.6 - 50.9 Mb (in green). (b) The LD pattern suggests that the contigs within this MisSeg (in red) should have been assembled (red arrow) nearby the contigs at 50.6 - 50.9 Mb (in green). (c) Rearrangement of the MisSeg to that position yields the expected LD decay pattern.

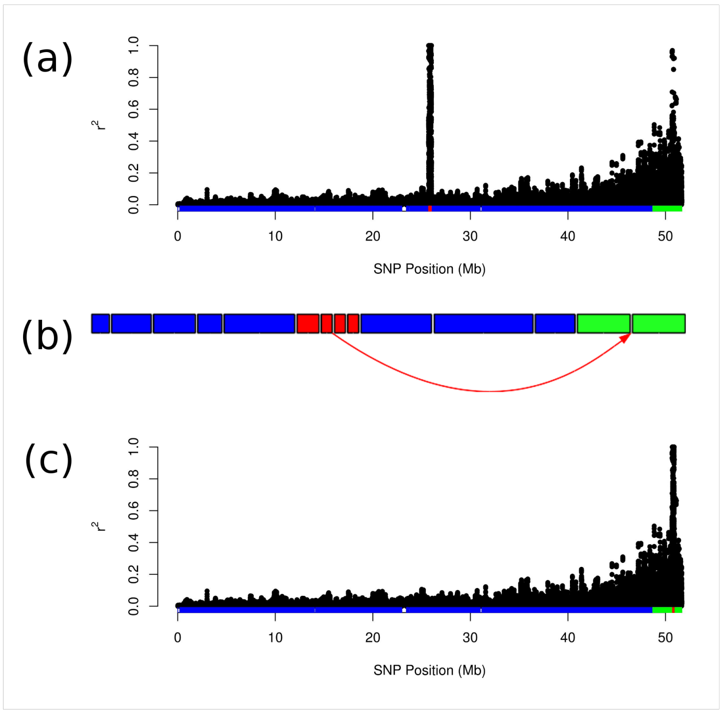

Supplement: Additional file 2: Figure S2. — Example of a Misassembled Segment (MisSeg). It shows the (un)expected linkage disequilibrium between markers in an specific segment of the genome when wrongly assembled (PDF 295 kb) [file 12864_2016_3049_MOESM2_ESM.pdf]
