# Supplementary material for: Revealing misassembled segments in the bovine reference genome by high resolution linkage disequilibrium scan
Source: BMC Genomics. 2016 Sep 5;17(1):705. doi: 10.1186/s12864-016-3049-8 (PMC5011828; doi:10.1186/s12864-016-3049-8)

Supplementary Figure S3 - Example of Partially Misassembled Contig (PMisCon). (a) A contig (yellow) on chromosome 1 (a.1) ranging from 94.57 Mb to 94.74 Mb shows high correlation with a segment (green) on chromosome 2 (a.2) position 7.9 - 8.0 Mb (right panel). (b) Although this contig exhibits an expected behavior of LD decay, a closer inspection reveals that the contig can be divided into two parts: a ”subcontig” (yellow) with expected LD behavior on chromosome 1 (b.1) and no high correlations with SNPs on chromosome 2 (b.2) and a second (grey) that shows an unexpected behaviour of LD decay on chromosome 1 (b.3) and has high correlation with a segment (green) on chromosome 2 (b.4). (c) Re-location of the grey ”subcontig” on chromosome 2 (c.2) corrects LD decay (c.1 and c.2).

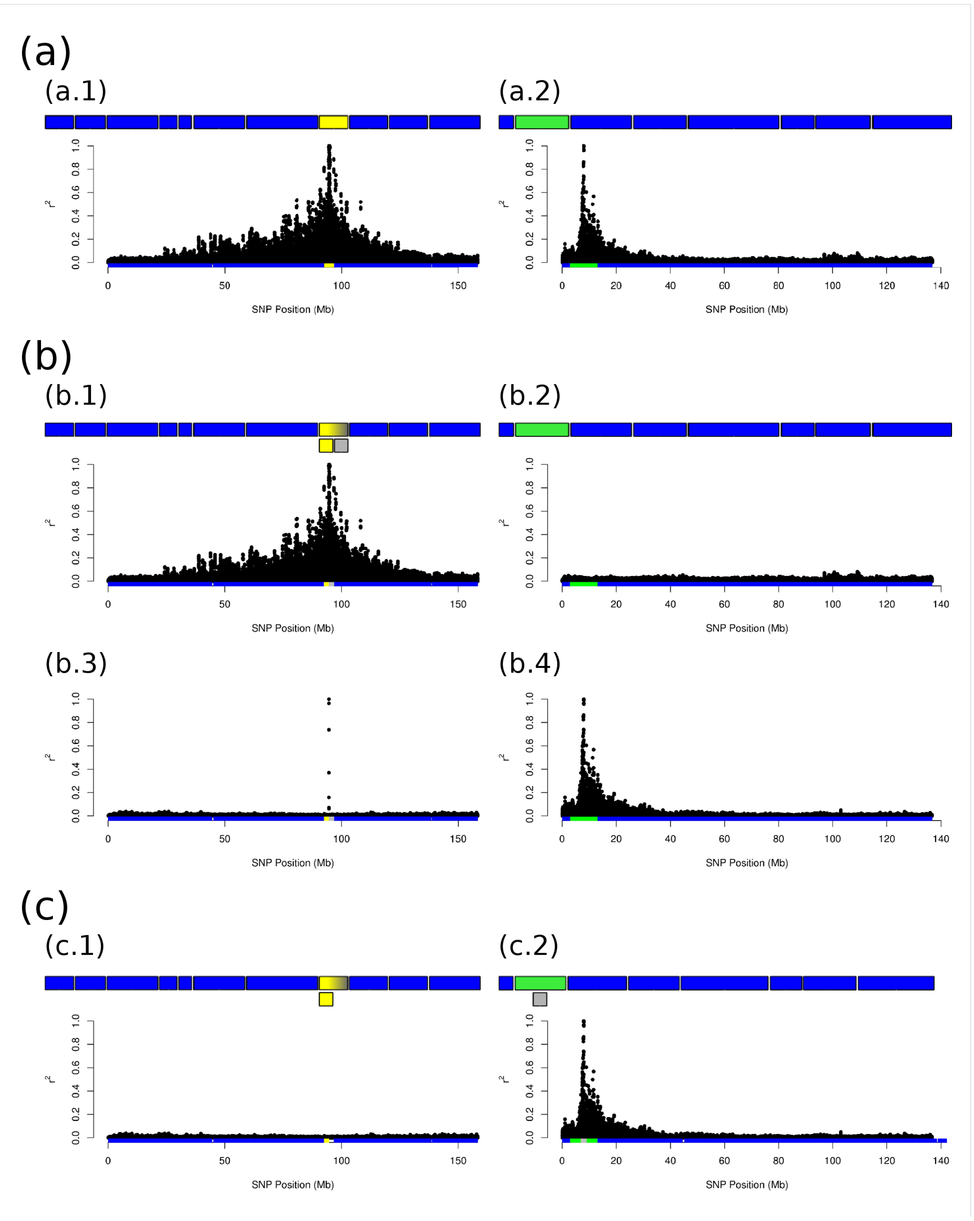

Supplement: Additional file 3: Figure S3. — Example of Partially Misassembled Contig (PmisCon). It shows the (un)expected linkage disequilibrium between markers in a partially misassembled contig (PDF 465 kb) [file 12864_2016_3049_MOESM3_ESM.pdf]
