# Supplementary material for: Revealing misassembled segments in the bovine reference genome by high resolution linkage disequilibrium scan
Source: BMC Genomics. 2016 Sep 5;17(1):705. doi: 10.1186/s12864-016-3049-8 (PMC5011828; doi:10.1186/s12864-016-3049-8)
Supplement: Additional file 5: Figure S4. — Misplaced SNPs. It presents all cases of misplaced SNPs found in this study (PDF 3034 kb) [file 12864_2016_3049_MOESM5_ESM.pdf]

Figure S4 Misplaced SNPs. Left and right columns represent misplacements (red dots) found in Holstein and Nellore, respectively.

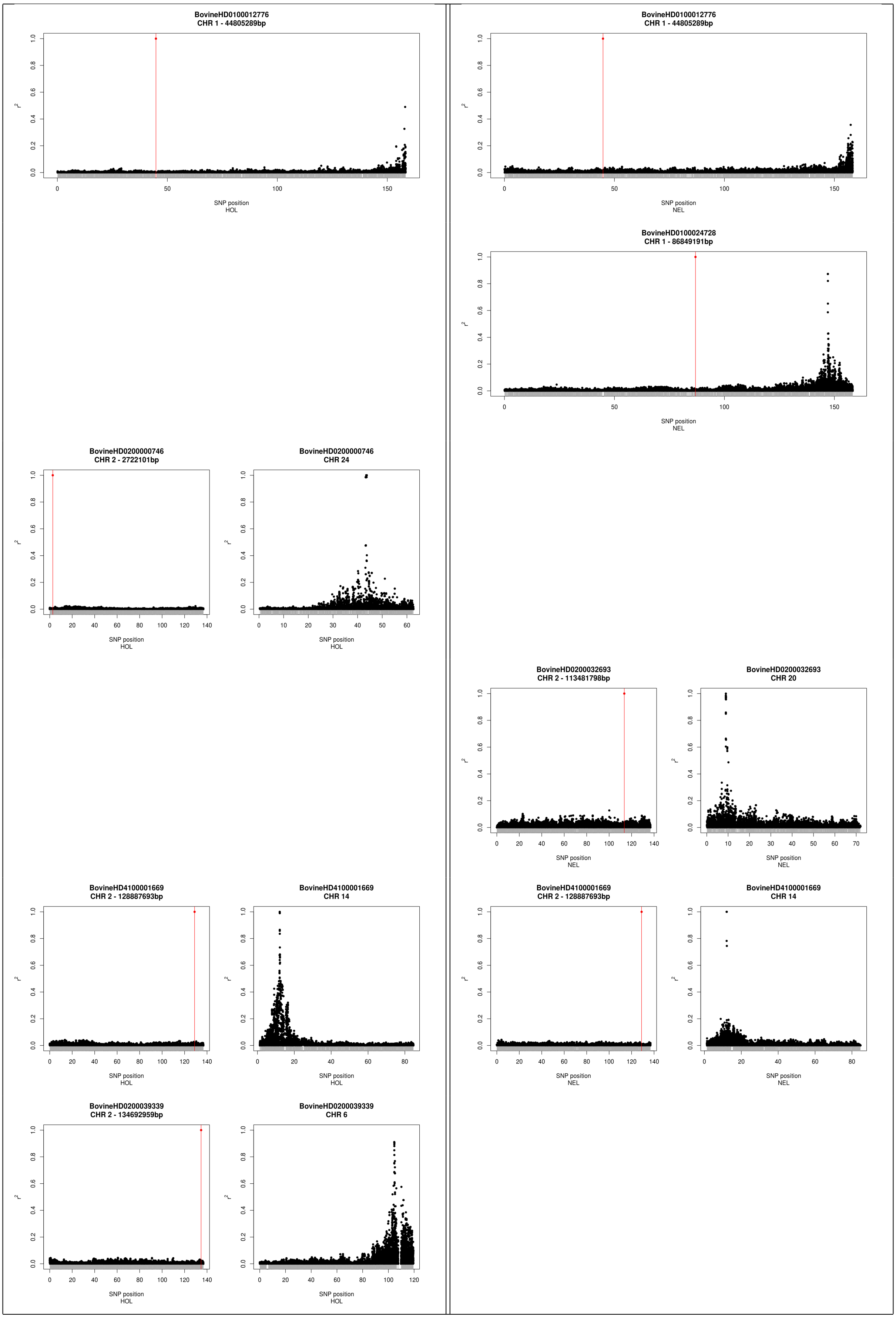

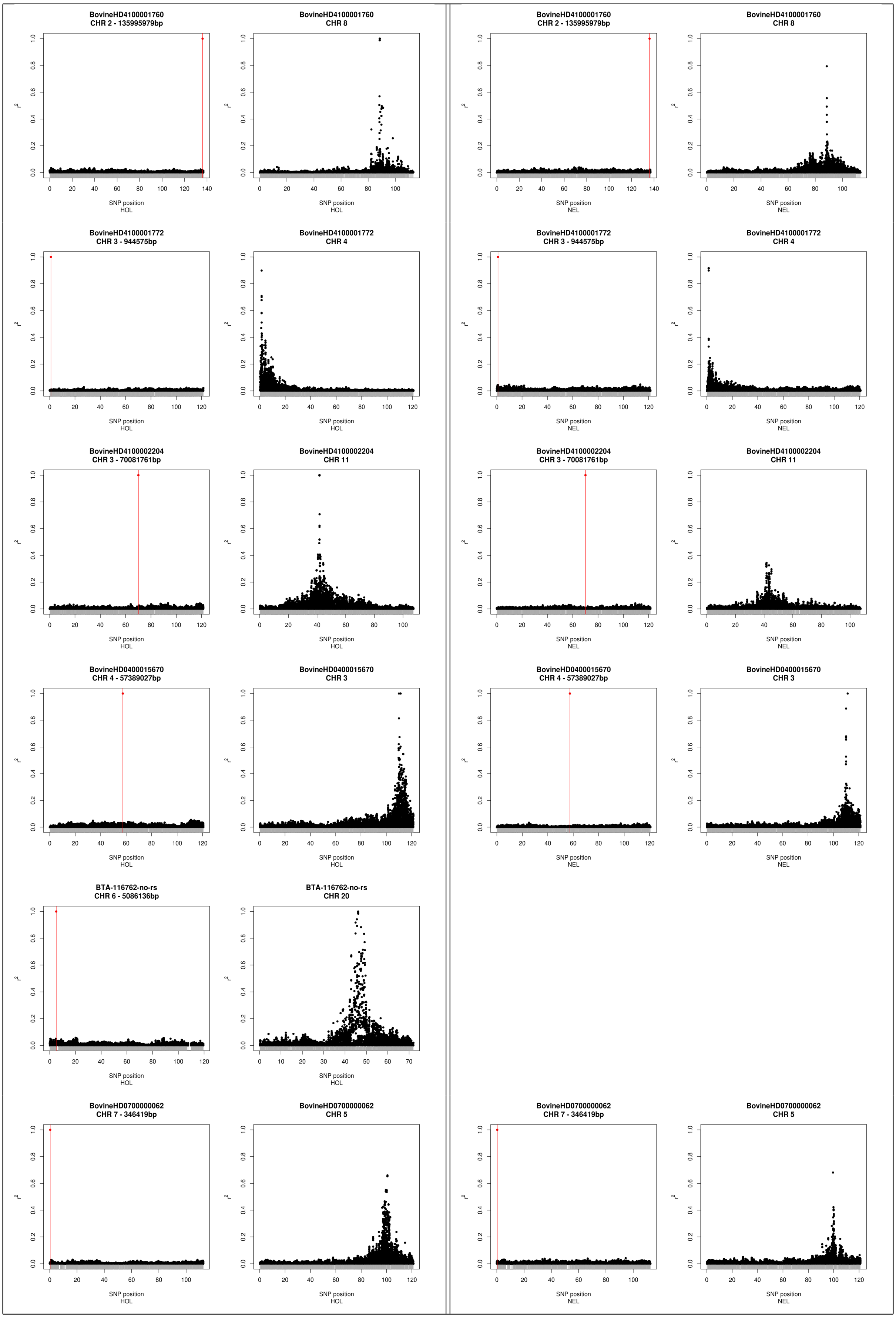
BovineHD4100001760
CHR 2 - 135995979bp
BovineHD4100001760
CHR 8
BovineHD4100001772
CHR 3 - 944575bp
BovineHD4100001772
CHR 4
BovineHD4100002204
CHR 3 - 70081761bp
BovineHD4100002204
CHR 11
BovineHD0400015670
CHR 4 - 57389027bp
BovineHD0400015670
CHR 3
BTA-116762-no-rs
CHR 6 - 5086136bp
BTA-116762-no-rs
CHR 20
BovineHD0700000062
CHR 7 - 346419bp
BovineHD0700000062
CHR 5
SNP position
HOL
SNP position
NEL

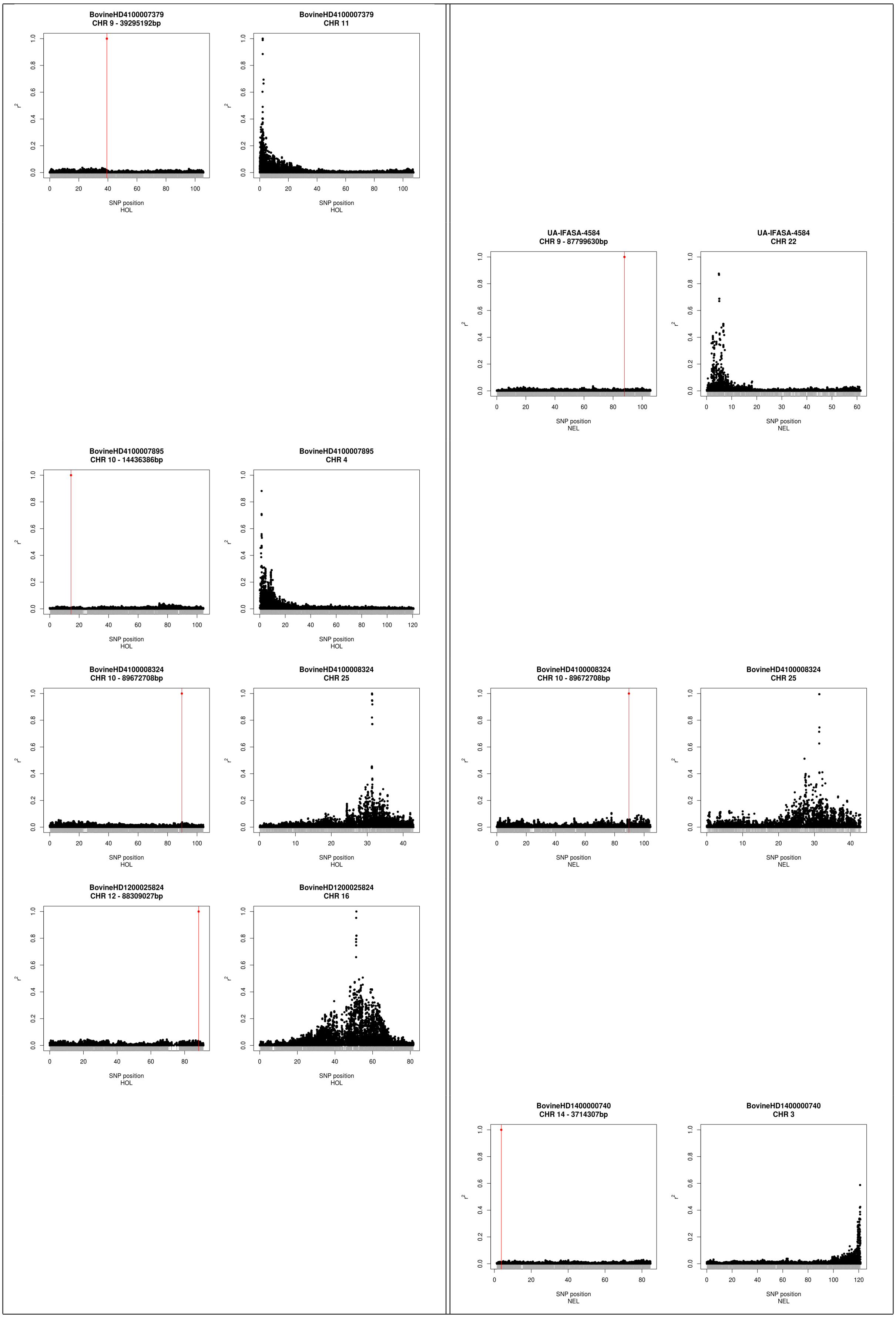
BovineHD4100007379
CHR 9 - 39295192bp
BovineHD4100007379
CHR 11
UA-IFASA-4584
CHR 9 - 87799630bp
UA-IFASA-4584
CHR 22
BovineHD4100007895
CHR 10 - 14436386bp
BovineHD4100007895
CHR 4
BovineHD4100008324
CHR 10 - 89672708bp
BovineHD4100008324
CHR 25
BovineHD4100008324
CHR 10 - 89672708bp
BovineHD4100008324
CHR 25
BovineHD1200025824
CHR 12 - 88309027bp
BovineHD1200025824
CHR 16
BovineHD1400000740
CHR 14 - 3714307bp
BovineHD1400000740
CHR 3
r^2
SNP position
HOL
NEL

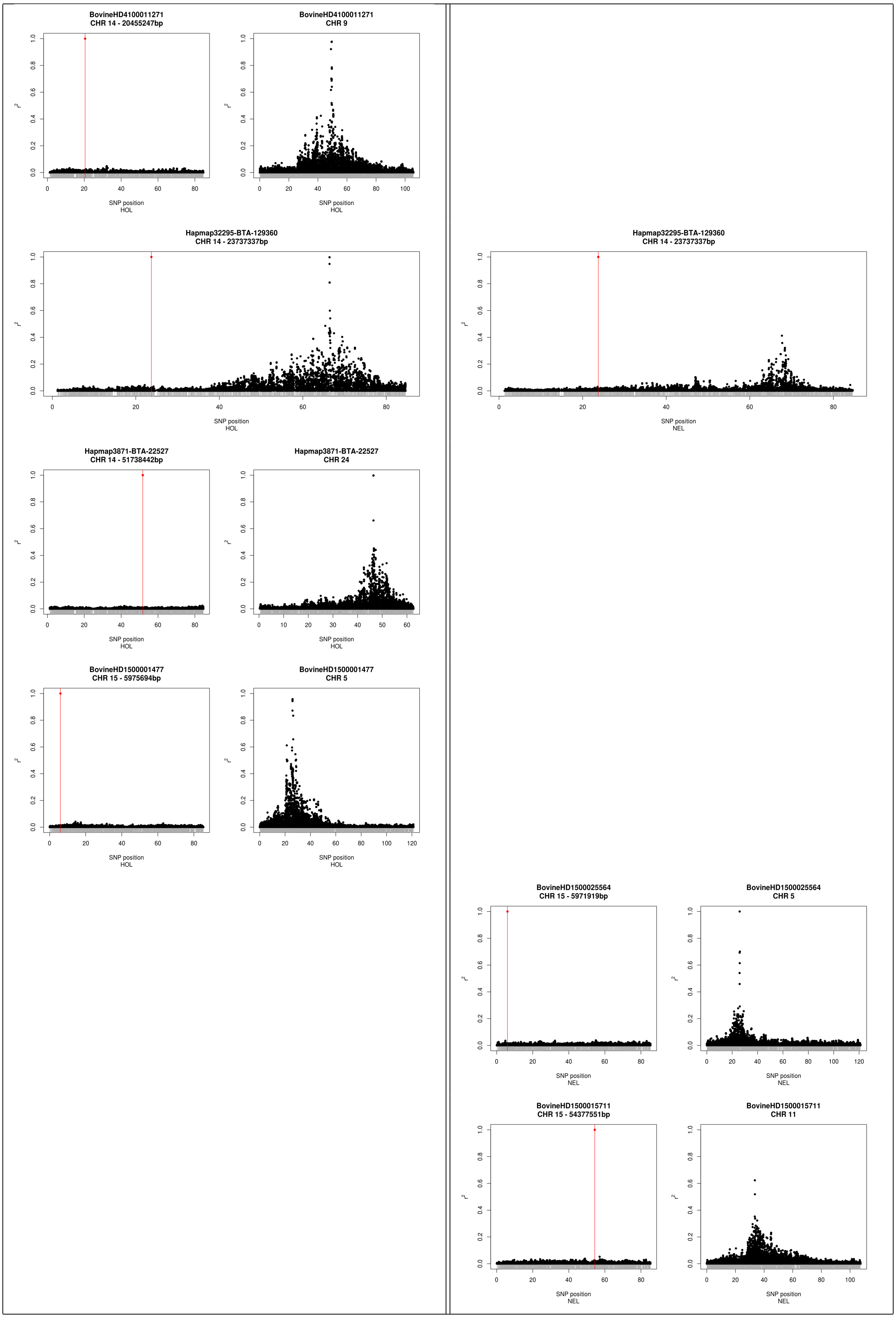
BovineHD4100011271
CHR 14 - 20455247bp
SNP position
HOL
BovineHD4100011271
CHR 9
SNP position
HOL
Hapmap32295-BTA-129360
CHR 14 - 23737337bp
SNP position
HOL
Hapmap32295-BTA-129360
CHR 14 - 23737337bp
SNP position
NEL
Hapmap3871-BTA-22527
CHR 14 - 51738442bp
SNP position
HOL
Hapmap3871-BTA-22527
CHR 24
SNP position
HOL
BovineHD1500001477
CHR 15 - 5975694bp
SNP position
HOL
BovineHD1500001477
CHR 5
SNP position
HOL
BovineHD1500025564
CHR 15 - 5971919bp
SNP position
NEL
BovineHD1500025564
CHR 5
SNP position
NEL
BovineHD1500015711
CHR 15 - 54377551bp
SNP position
NEL
BovineHD1500015711
CHR 11
SNP position
NEL

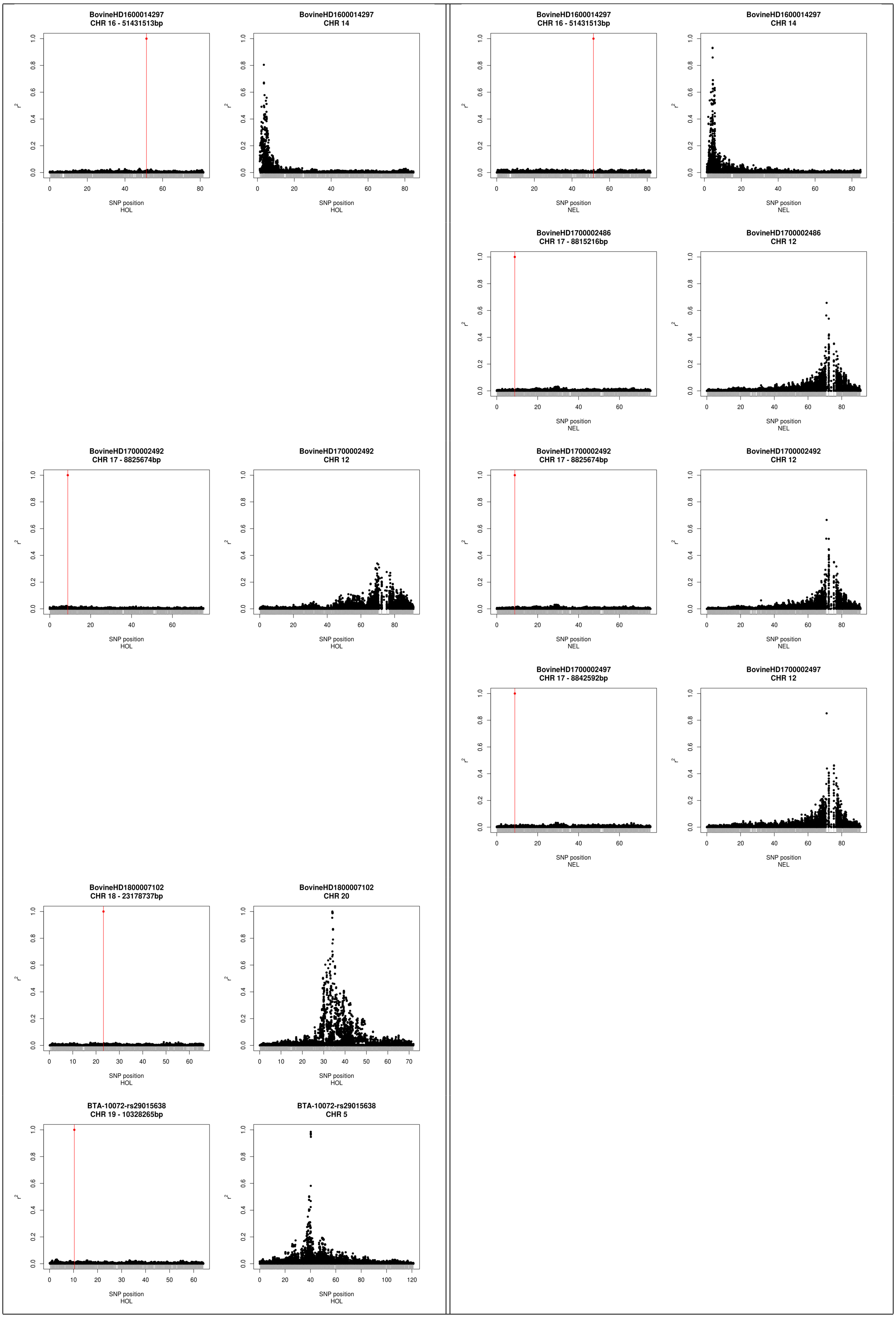
BovineHD1600014297
CHR 16 - 51431513bp
BovineHD1600014297
CHR 14
BovineHD1700002492
CHR 17 - 8825674bp
BovineHD1700002492
CHR 12
BovineHD1800007102
CHR 18 - 23178737bp
BovineHD1800007102
CHR 20
BTA-10072-rs29015638
CHR 19 - 10328265bp
BTA-10072-rs29015638
CHR 5
SNP position
HOL
BovineHD1600014297
CHR 16 - 51431513bp
BovineHD1600014297
CHR 14
BovineHD1700002486
CHR 17 - 8815216bp
BovineHD1700002486
CHR 12
BovineHD1700002492
CHR 17 - 8825674bp
BovineHD1700002492
CHR 12
BovineHD1700002497
CHR 17 - 8842592bp
BovineHD1700002497
CHR 12
SNP position
NEL
r^2

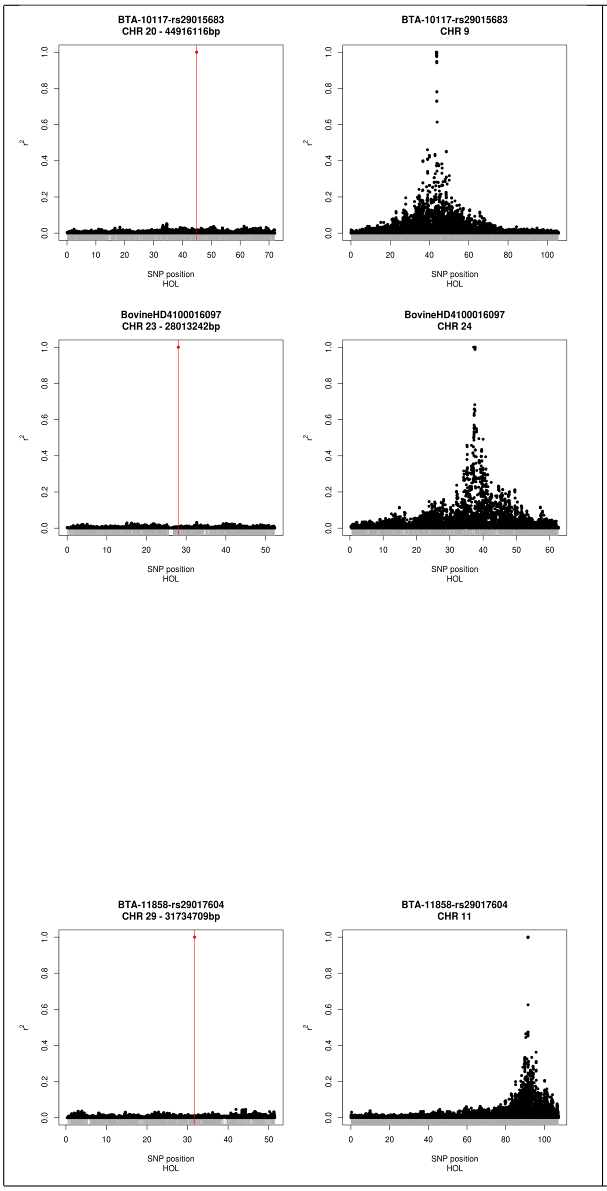
BTA-10117-rs29015683
CHR 20 - 44916116bp
BTA-10117-rs29015683
CHR 9
BovineHD4100016097
CHR 23 - 28013242bp
BovineHD4100016097
CHR 24
BTA-11858-rs29017604
CHR 29 - 31734709bp
BTA-11858-rs29017604
CHR 11
r²
SNP position
HOL

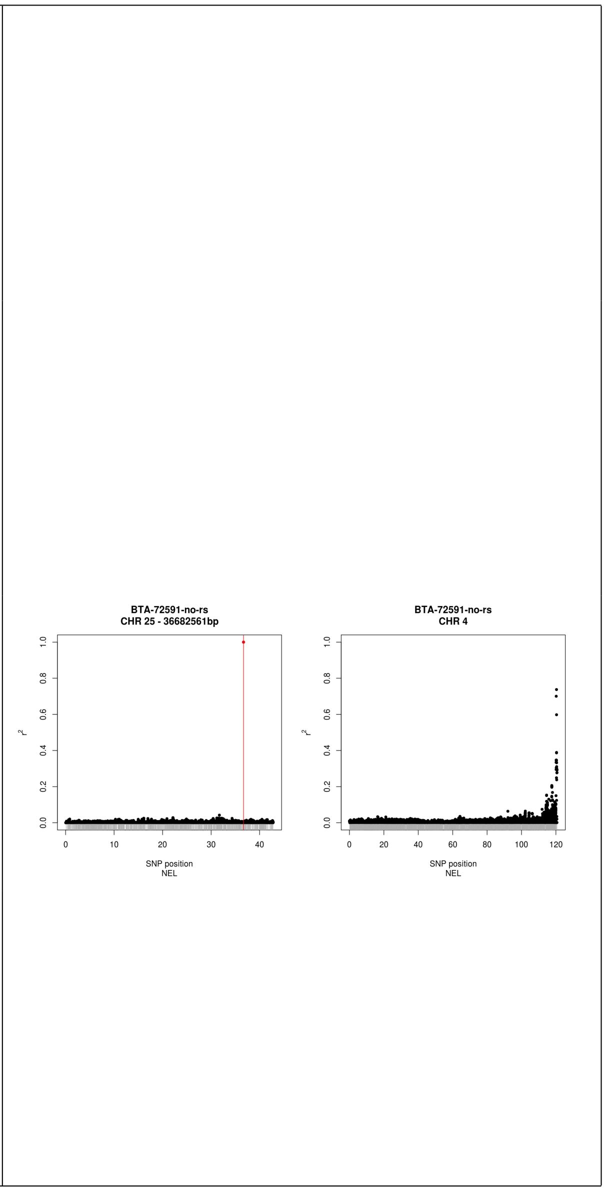
BTA-72591-no-rs
CHR 25 - 36682561bp
BTA-72591-no-rs
CHR 4
r²
SNP position
NEL
