# Supplementary material for: Revealing misassembled segments in the bovine reference genome by high resolution linkage disequilibrium scan
Source: BMC Genomics. 2016 Sep 5;17(1):705. doi: 10.1186/s12864-016-3049-8 (PMC5011828; doi:10.1186/s12864-016-3049-8)
Supplement: Additional file 6: Figure S5. — Misassembled segment (MisSeg). It presents all cases of misassembled segments of the reference genome found in this study (PDF 4176 kb) [file 12864_2016_3049_MOESM6_ESM.pdf]

Figure S5 Misassembled Segment (MisSeg). Left and right columns represent misplacements (red dots) found in Holstein and Nellore, respectively.

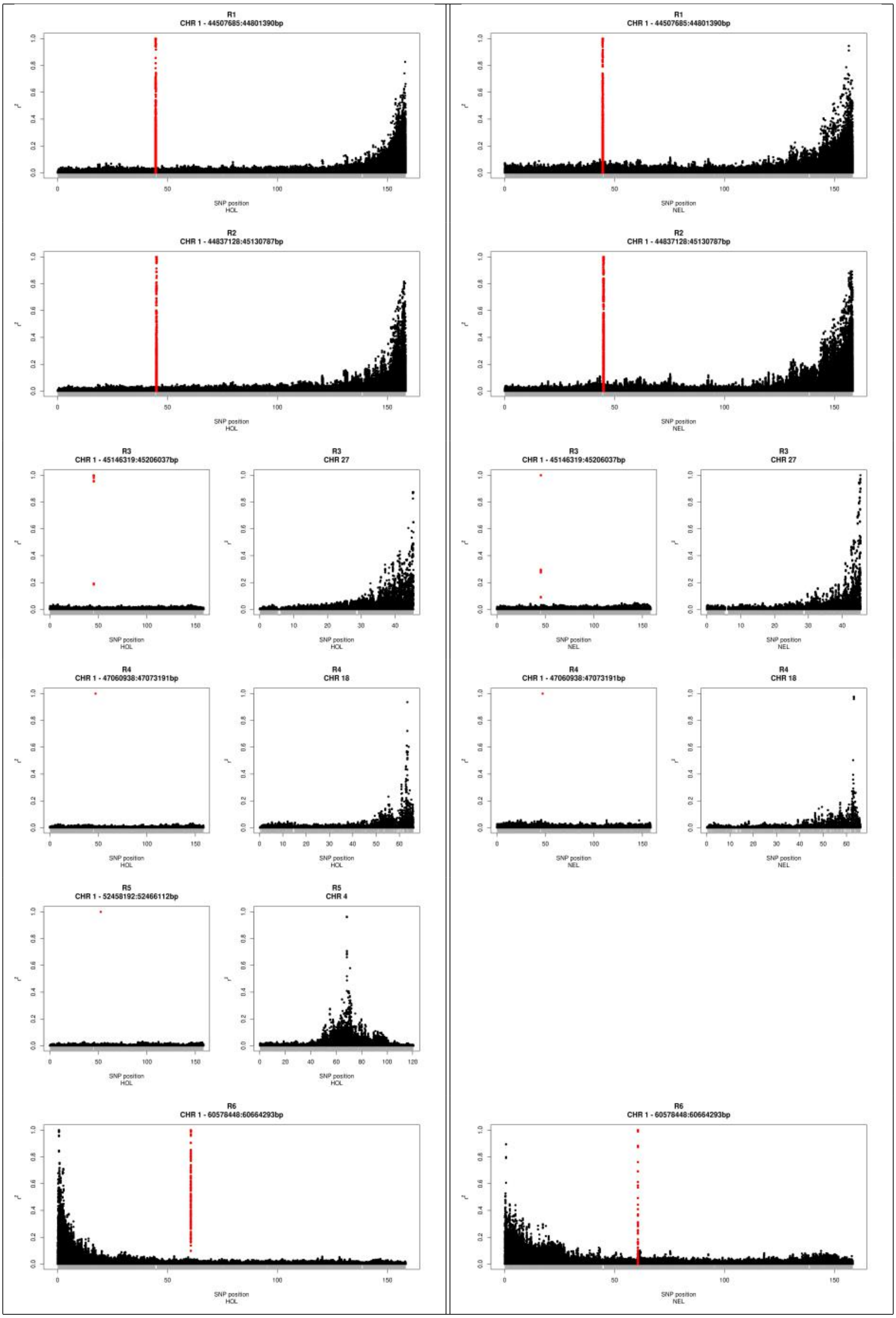

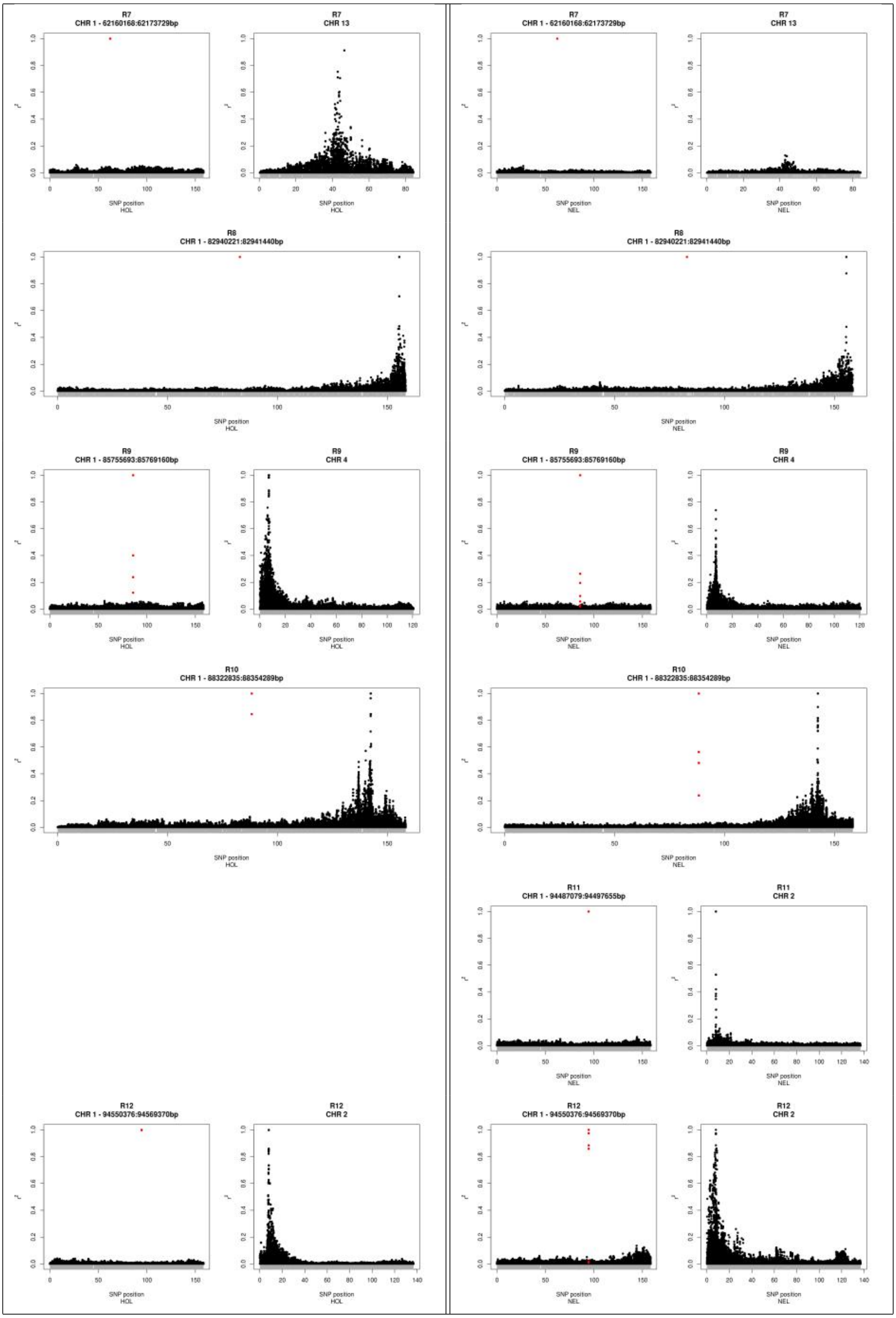
R7
CHR 1 - 62160168:62173729bp
R7
CHR 13
R8
CHR 1 - 82940221:82941440bp
R9
CHR 1 - 85755693:85769160bp
R9
CHR 4
R10
CHR 1 - 88322835:88354289bp
R11
CHR 1 - 94487079:94497655bp
R11
CHR 2
R12
CHR 1 - 94550376:94569370bp
R12
CHR 2
SNP position
HOL
NEL

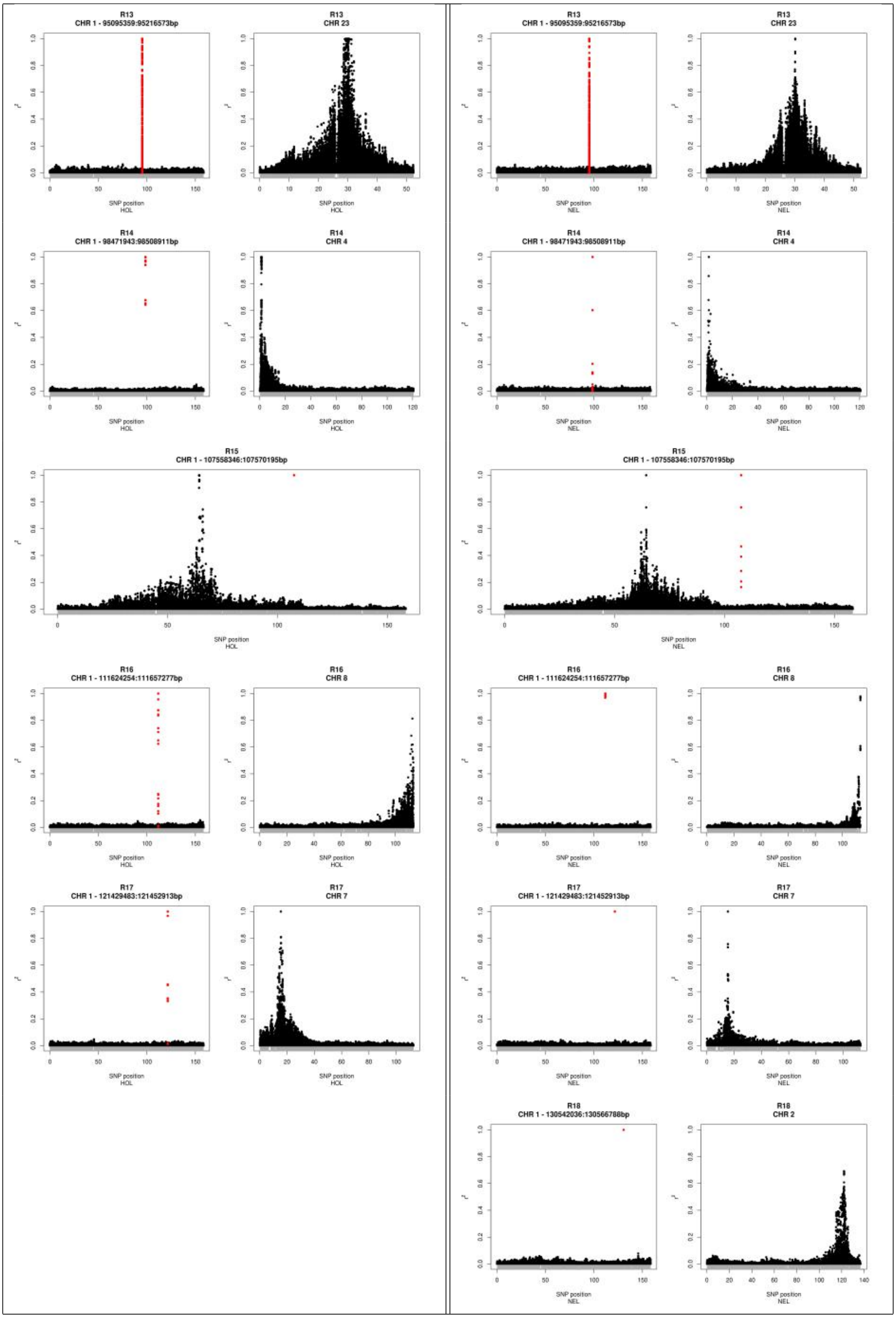
R13
CHR 1 - 95095359:95216573bp
R13
CHR 23
R14
CHR 1 - 98471943:98508911bp
R14
CHR 4
R15
CHR 1 - 107558346:107570195bp
R16
CHR 1 - 111624254:111657277bp
R16
CHR 8
R17
CHR 1 - 121429483:121452913bp
R17
CHR 7
R18
CHR 1 - 130542036:130566788bp
R18
CHR 2
SNP position
HOL
NEL
r²

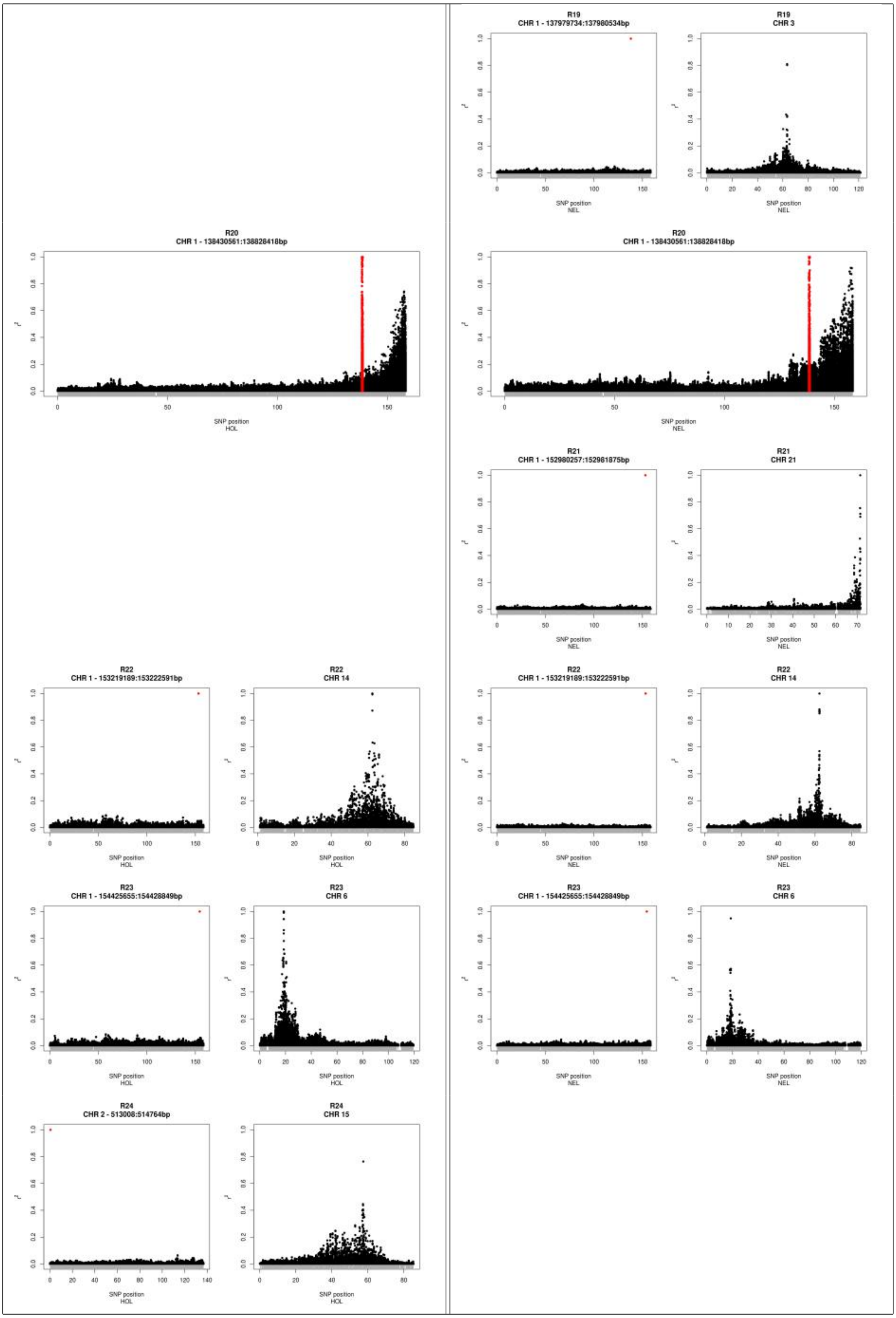
R19
CHR 1 - 137979734:137980534bp
R19
CHR 3
R20
CHR 1 - 138430561:138828418bp
R20
CHR 1 - 138430561:138828418bp
R21
CHR 1 - 152980257:152981875bp
R21
CHR 21
R22
CHR 1 - 153219189:153222591bp
R22
CHR 14
R22
CHR 1 - 153219189:153222591bp
R22
CHR 14
R23
CHR 1 - 154425655:154428849bp
R23
CHR 6
R23
CHR 1 - 154425655:154428849bp
R23
CHR 6
R24
CHR 2 - 513008:514764bp
R24
CHR 15
SNP position
HOL
NEL
r^2

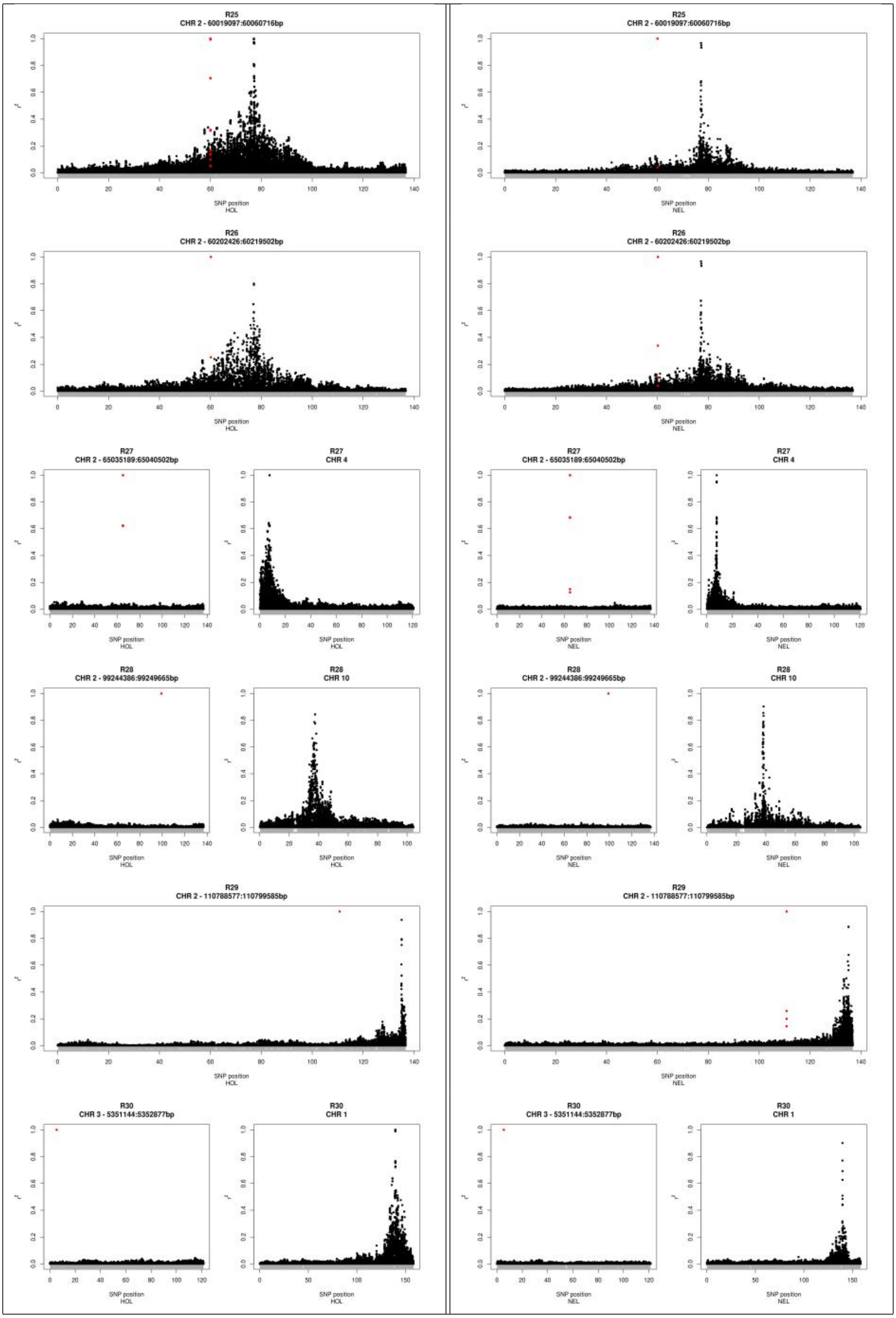
R25
CHR 2 - 60019097:60060716bp
SNP position
HOL
R25
CHR 2 - 60019097:60060716bp
SNP position
NEL
R26
CHR 2 - 60202426:60219502bp
SNP position
HOL
R26
CHR 2 - 60202426:60219502bp
SNP position
NEL
R27
CHR 2 - 65035189:65040502bp
SNP position
HOL
R27
CHR 4
SNP position
HOL
R27
CHR 2 - 65035189:65040502bp
SNP position
NEL
R27
CHR 4
SNP position
NEL
R28
CHR 2 - 99244386:99249665bp
SNP position
HOL
R28
CHR 10
SNP position
HOL
R28
CHR 2 - 99244386:99249665bp
SNP position
NEL
R28
CHR 10
SNP position
NEL
R29
CHR 2 - 110788577:110799585bp
SNP position
HOL
R29
CHR 2 - 110788577:110799585bp
SNP position
NEL
R30
CHR 3 - 5351144:5352877bp
SNP position
HOL
R30
CHR 1
SNP position
HOL
R30
CHR 3 - 5351144:5352877bp
SNP position
NEL
R30
CHR 1
SNP position
NEL

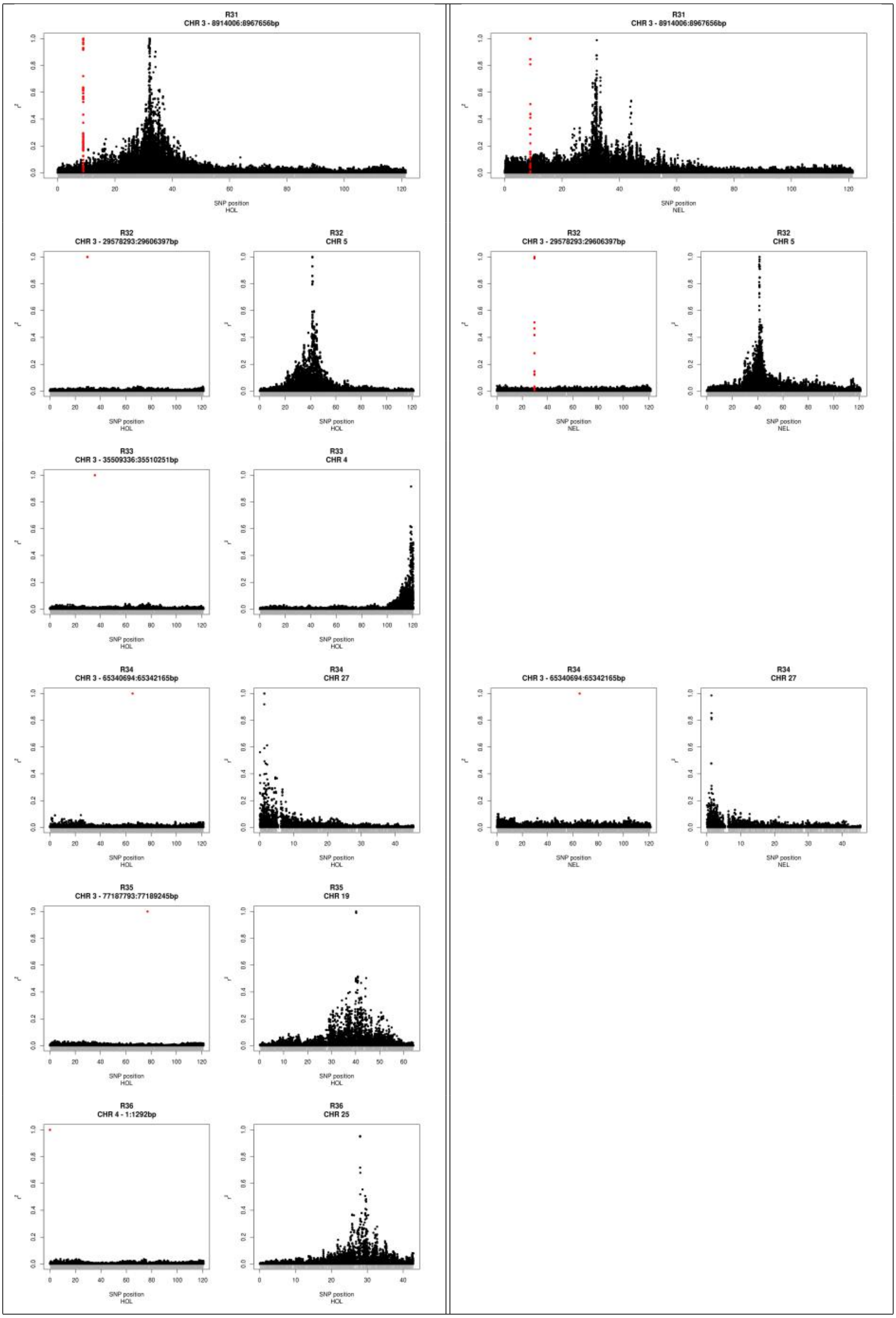
R31
CHR 3 - 8914006:8967656bp
SNP position
HOL
R31
CHR 3 - 8914006:8967656bp
SNP position
NEL
R32
CHR 3 - 29578293:29606397bp
SNP position
HOL
R32
CHR 5
SNP position
HOL
R32
CHR 3 - 29578293:29606397bp
SNP position
NEL
R32
CHR 5
SNP position
NEL
R33
CHR 3 - 35509336:35510251bp
SNP position
HOL
R33
CHR 4
SNP position
HOL
R34
CHR 3 - 65340694:65342165bp
SNP position
HOL
R34
CHR 27
SNP position
HOL
R34
CHR 3 - 65340694:65342165bp
SNP position
NEL
R34
CHR 27
SNP position
NEL
R35
CHR 3 - 77187793:77189245bp
SNP position
HOL
R35
CHR 19
SNP position
HOL
R36
CHR 4 - 1:1292bp
SNP position
HOL
R36
CHR 25
SNP position
HOL

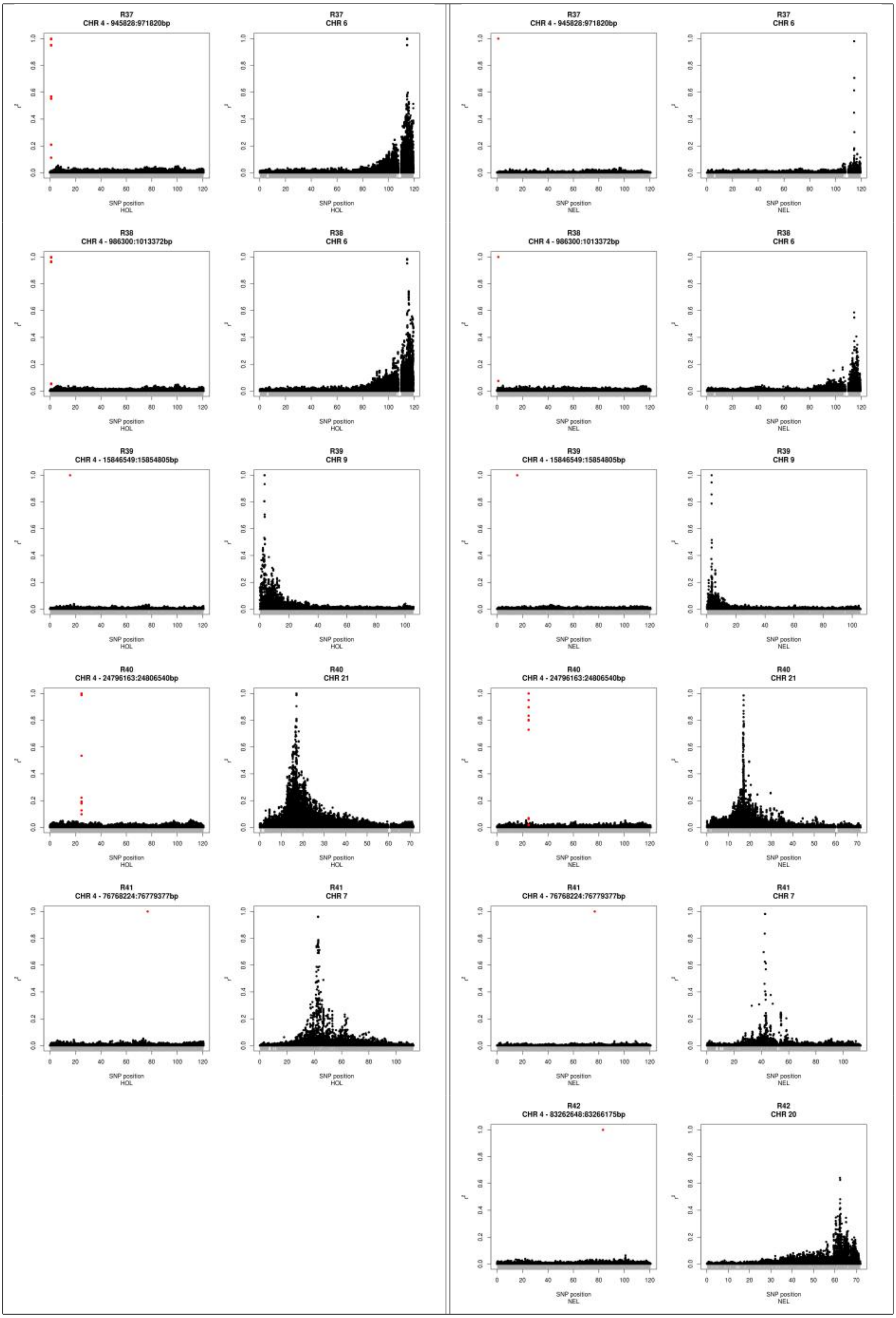
R37
CHR 4 - 945828:971820bp
R37
CHR 6
R38
CHR 4 - 986300:1013372bp
R38
CHR 6
R39
CHR 4 - 15846549:15854805bp
R39
CHR 9
R40
CHR 4 - 24796163:24806540bp
R40
CHR 21
R41
CHR 4 - 76768224:76779377bp
R41
CHR 7
R42
CHR 4 - 83262648:83266175bp
R42
CHR 20
SNP position
HOL
NEL
r²

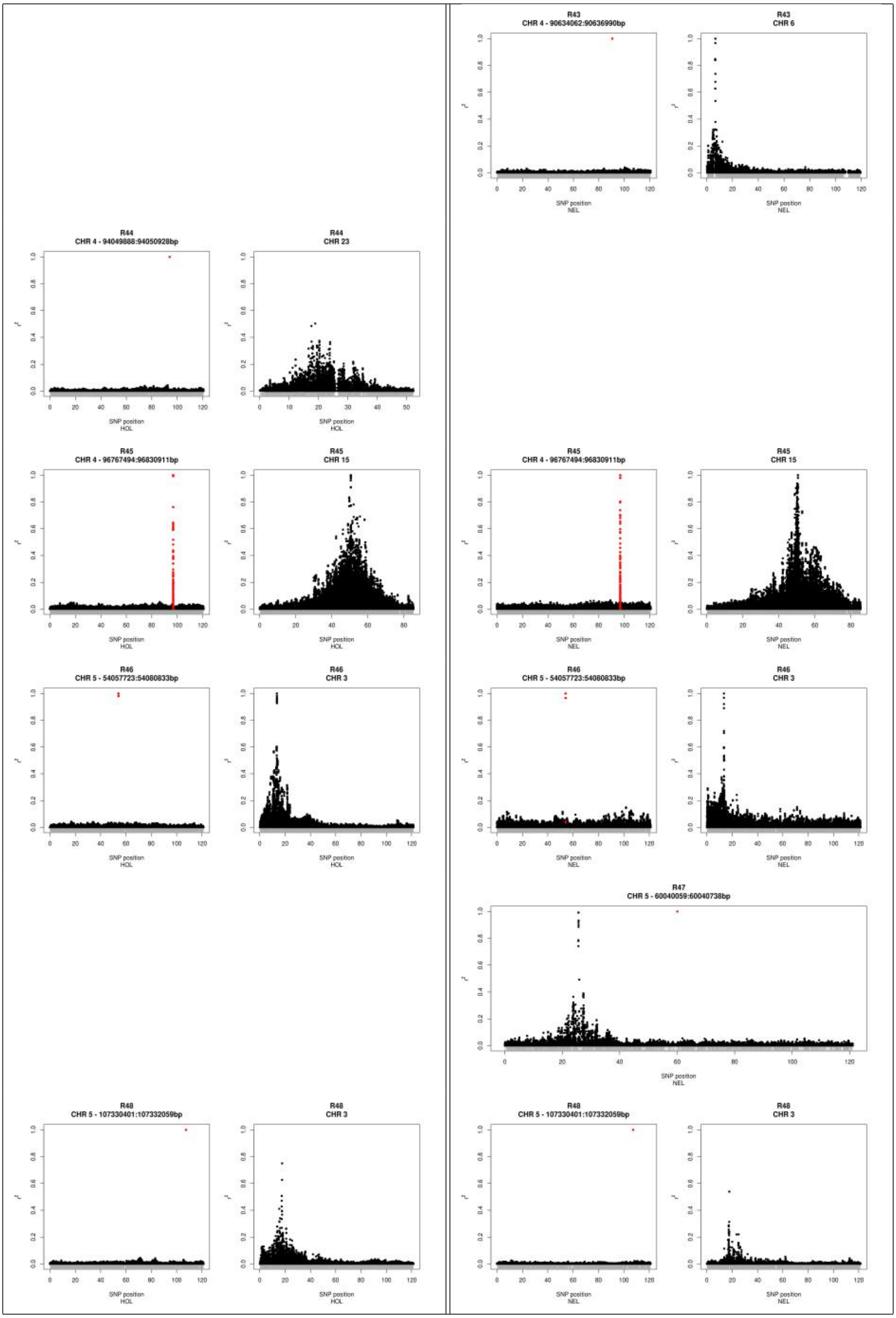
R43
CHR 4 - 90634062:90636990bp
SNP position
NEL
R43
CHR 6
SNP position
NEL
R44
CHR 4 - 94049888:94050928bp
SNP position
HOL
R44
CHR 23
SNP position
HOL
R45
CHR 4 - 96767494:96830911bp
SNP position
HOL
R45
CHR 15
SNP position
HOL
R45
CHR 4 - 96767494:96830911bp
SNP position
NEL
R45
CHR 15
SNP position
NEL
R46
CHR 5 - 54057723:54080833bp
SNP position
HOL
R46
CHR 3
SNP position
HOL
R46
CHR 5 - 54057723:54080833bp
SNP position
NEL
R46
CHR 3
SNP position
NEL
R47
CHR 5 - 60040059:60040738bp
SNP position
NEL
R48
CHR 5 - 107330401:107332059bp
SNP position
HOL
R48
CHR 3
SNP position
HOL
R48
CHR 5 - 107330401:107332059bp
SNP position
NEL
R48
CHR 3
SNP position
NEL

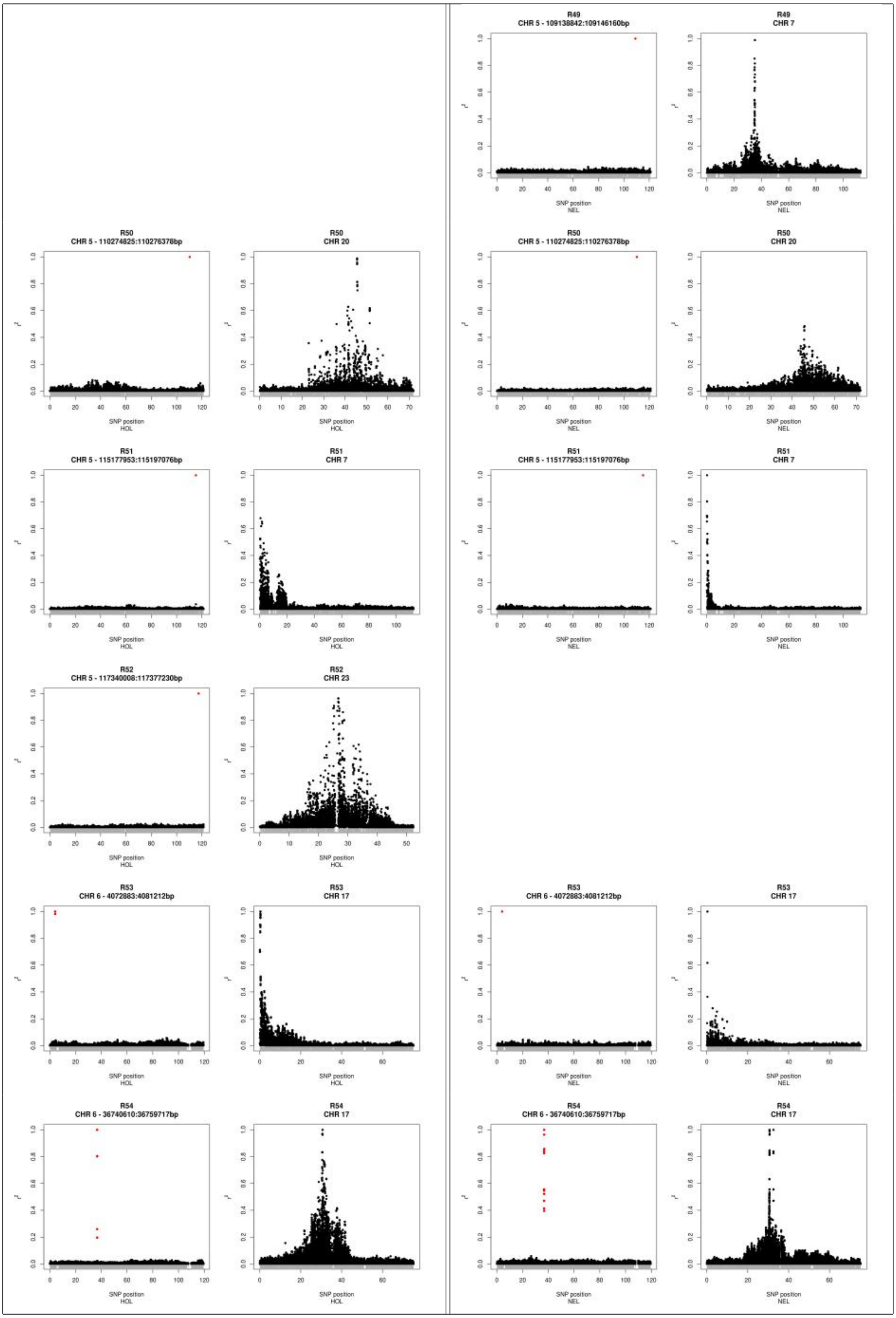
R49
CHR 5 - 109138842:109146160bp
R49
CHR 7
R50
CHR 5 - 110274825:110276378bp
R50
CHR 20
R51
CHR 5 - 115177953:115197076bp
R51
CHR 7
R52
CHR 5 - 117340008:117377230bp
R52
CHR 23
R53
CHR 6 - 4072883:4081212bp
R53
CHR 17
R54
CHR 6 - 36740610:36759717bp
R54
CHR 17
r²
SNP position
HOL
NEL

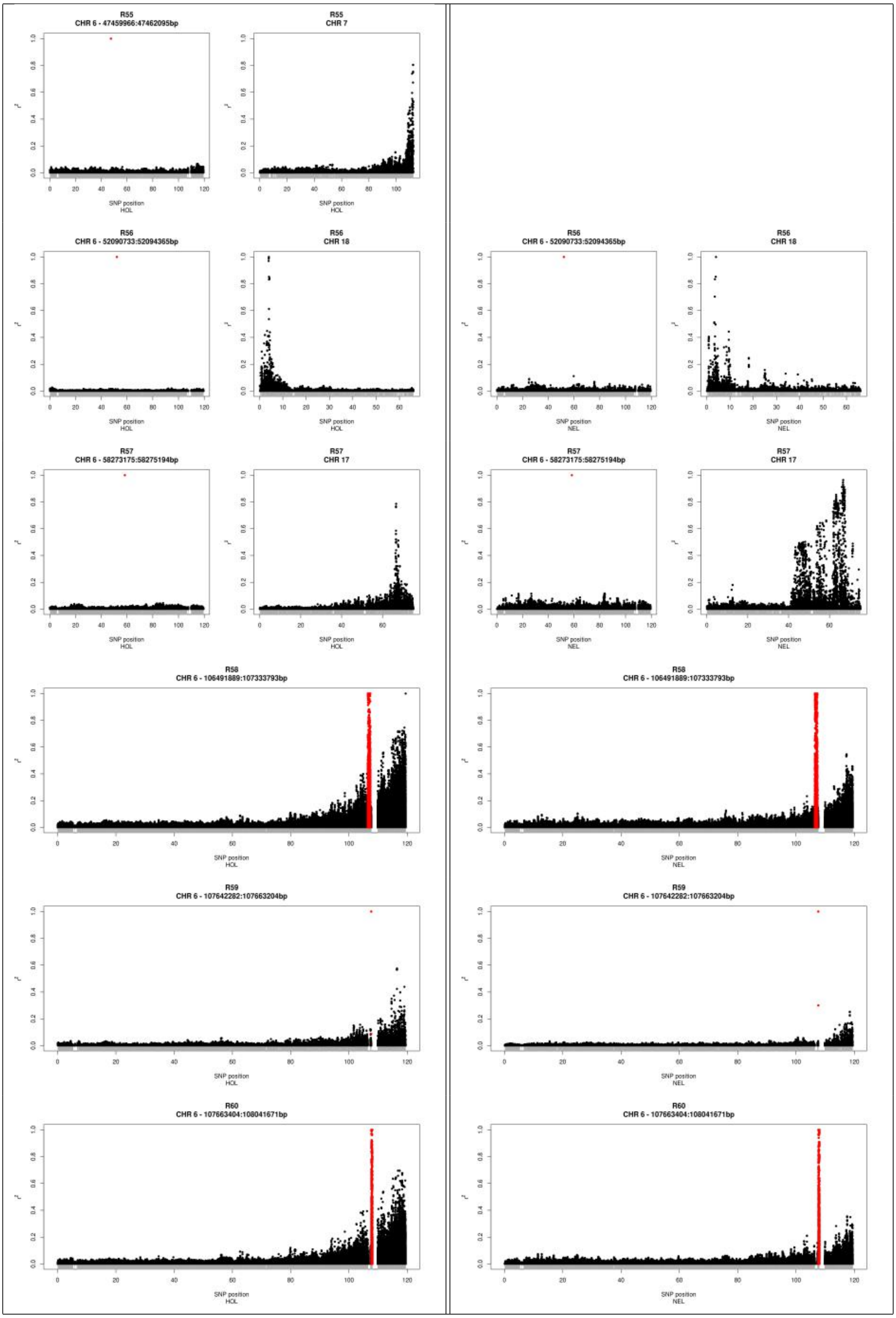
R55
CHR 6 - 47459966:47462095bp
R55
CHR 7
R56
CHR 6 - 52090733:52094365bp
R56
CHR 18
R56
CHR 6 - 52090733:52094365bp
R56
CHR 18
R57
CHR 6 - 58273175:58275194bp
R57
CHR 17
R57
CHR 6 - 58273175:58275194bp
R57
CHR 17
R58
CHR 6 - 106491889:107333793bp
R58
CHR 6 - 106491889:107333793bp
R59
CHR 6 - 107642282:107663204bp
R59
CHR 6 - 107642282:107663204bp
R60
CHR 6 - 107663404:108041671bp
R60
CHR 6 - 107663404:108041671bp
SNP position
HOL
NEL
r^2

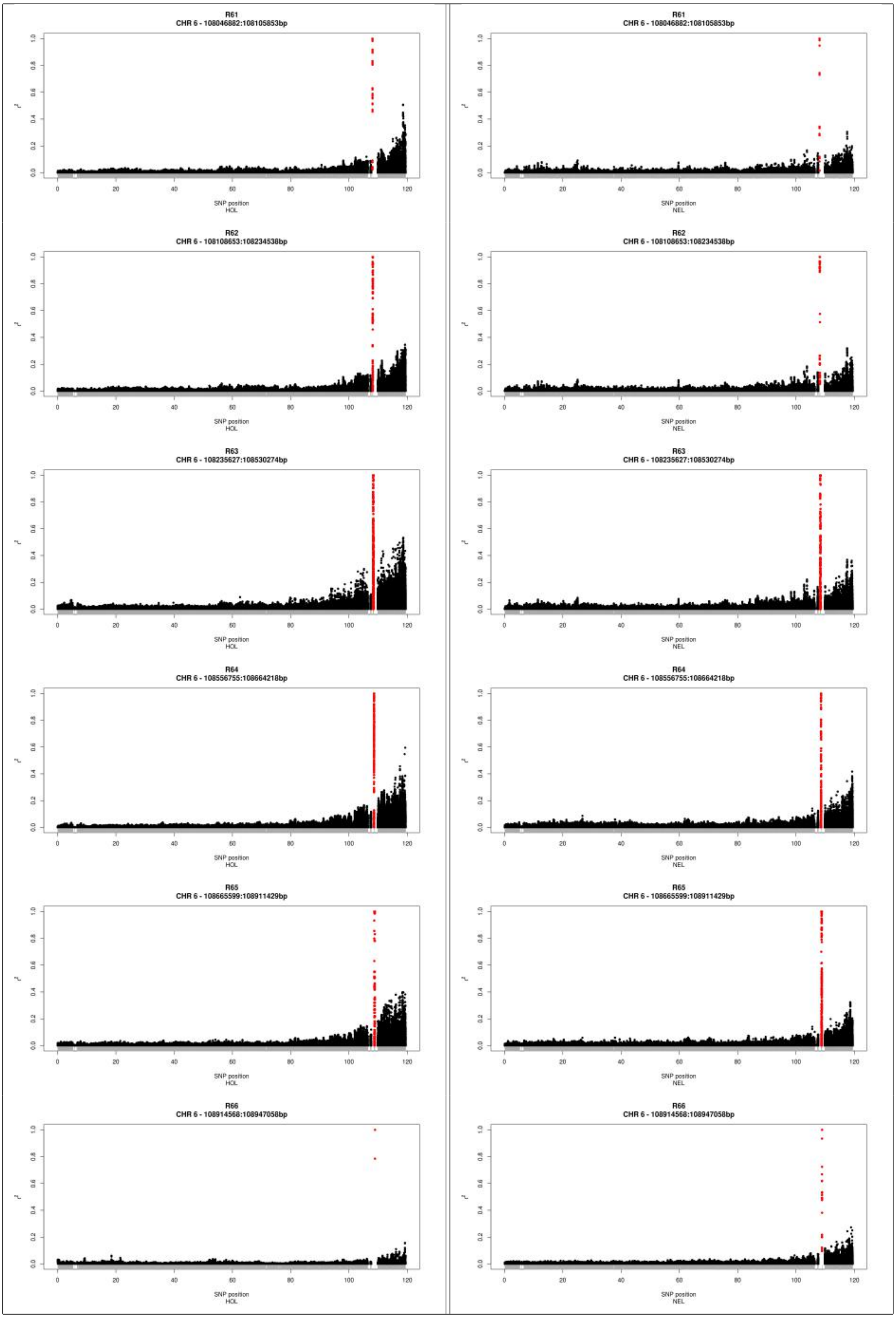
R61
CHR 6 - 108046882:108105853bp
SNP position
HOL
R61
CHR 6 - 108046882:108105853bp
SNP position
NEL
R62
CHR 6 - 108108653:108234538bp
SNP position
HOL
R62
CHR 6 - 108108653:108234538bp
SNP position
NEL
R63
CHR 6 - 108235627:108530274bp
SNP position
HOL
R63
CHR 6 - 108235627:108530274bp
SNP position
NEL
R64
CHR 6 - 108556755:108664218bp
SNP position
HOL
R64
CHR 6 - 108556755:108664218bp
SNP position
NEL
R65
CHR 6 - 108665599:108911429bp
SNP position
HOL
R65
CHR 6 - 108665599:108911429bp
SNP position
NEL
R66
CHR 6 - 108914568:108947058bp
SNP position
HOL
R66
CHR 6 - 108914568:108947058bp
SNP position
NEL

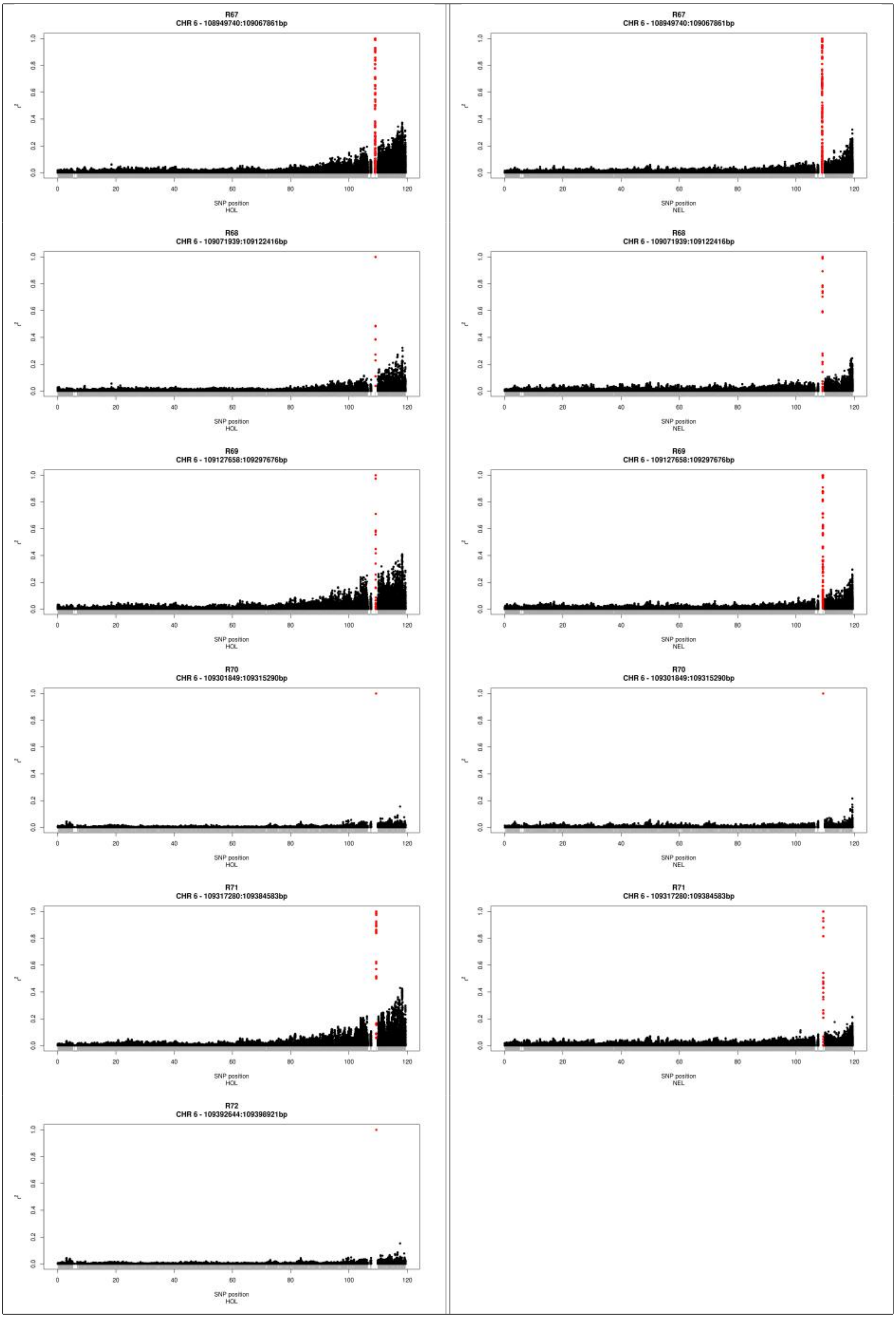
R67
CHR 6 - 108949740:109067861bp
SNP position
HOL
R67
CHR 6 - 108949740:109067861bp
SNP position
NEL
R68
CHR 6 - 109071939:109122416bp
SNP position
HOL
R68
CHR 6 - 109071939:109122416bp
SNP position
NEL
R69
CHR 6 - 109127658:109297676bp
SNP position
HOL
R69
CHR 6 - 109127658:109297676bp
SNP position
NEL
R70
CHR 6 - 109301849:109315290bp
SNP position
HOL
R70
CHR 6 - 109301849:109315290bp
SNP position
NEL
R71
CHR 6 - 109317280:109384583bp
SNP position
HOL
R71
CHR 6 - 109317280:109384583bp
SNP position
NEL
R72
CHR 6 - 109392644:109398921bp
SNP position
HOL

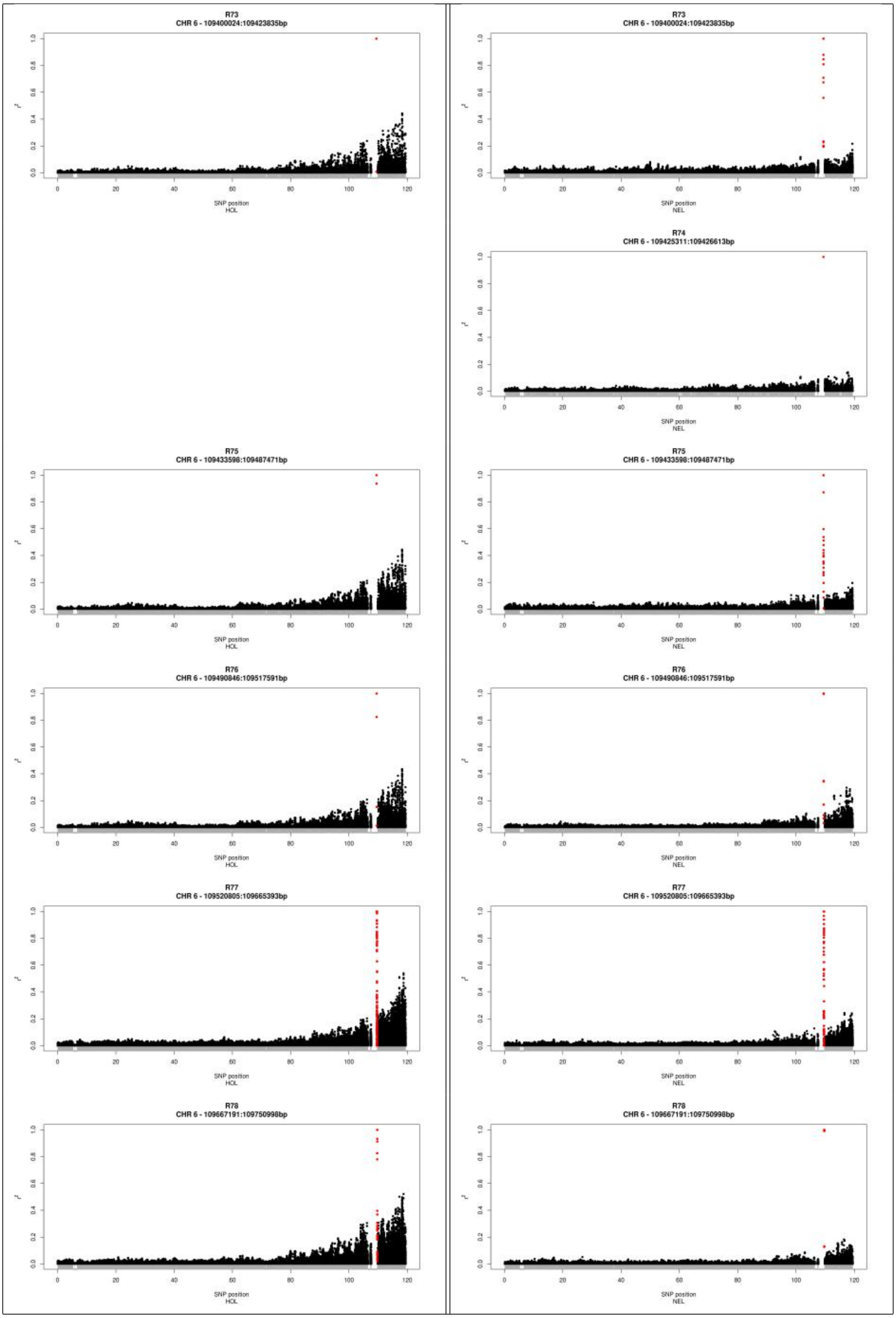
R73
CHR 6 - 109400024:109423835bp
SNP position
HOL
R73
CHR 6 - 109400024:109423835bp
SNP position
NEL
R74
CHR 6 - 109425311:109426613bp
SNP position
NEL
R75
CHR 6 - 109433598:109487471bp
SNP position
HOL
R75
CHR 6 - 109433598:109487471bp
SNP position
NEL
R76
CHR 6 - 109490846:109517591bp
SNP position
HOL
R76
CHR 6 - 109490846:109517591bp
SNP position
NEL
R77
CHR 6 - 109520805:109665393bp
SNP position
HOL
R77
CHR 6 - 109520805:109665393bp
SNP position
NEL
R78
CHR 6 - 109667191:109750998bp
SNP position
HOL
R78
CHR 6 - 109667191:109750998bp
SNP position
NEL

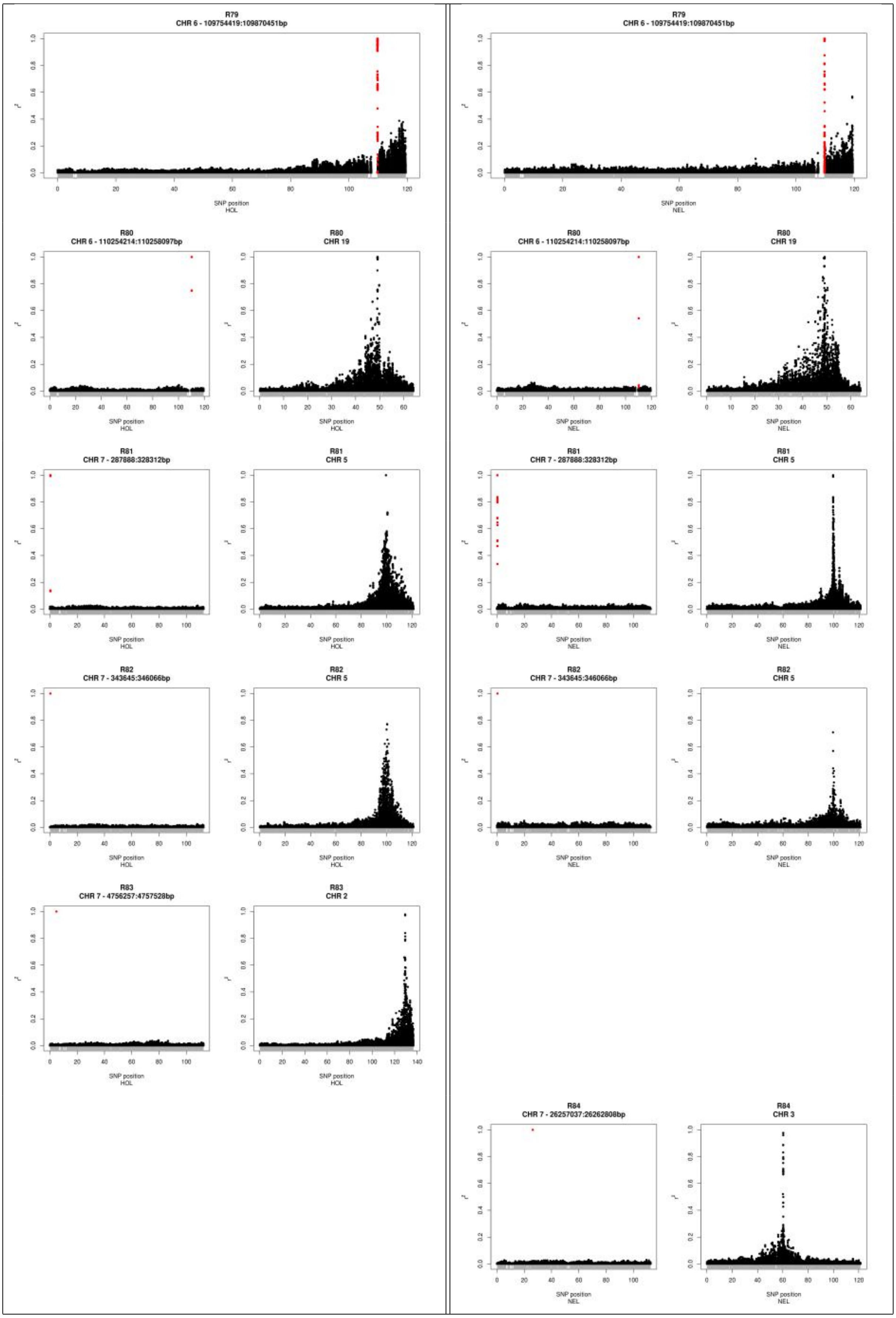
R79
CHR 6 - 109754419:109870451bp
SNP position
HOL
R79
CHR 6 - 109754419:109870451bp
SNP position
NEL
R80
CHR 6 - 110254214:110258097bp
R80
CHR 19
SNP position
HOL
R80
CHR 6 - 110254214:110258097bp
R80
CHR 19
SNP position
NEL
R81
CHR 7 - 287888:328312bp
R81
CHR 5
SNP position
HOL
R81
CHR 7 - 287888:328312bp
R81
CHR 5
SNP position
NEL
R82
CHR 7 - 343645:346066bp
R82
CHR 5
SNP position
HOL
R82
CHR 7 - 343645:346066bp
R82
CHR 5
SNP position
NEL
R83
CHR 7 - 4756257:4757528bp
R83
CHR 2
SNP position
HOL
R84
CHR 7 - 26257037:26262808bp
R84
CHR 3
SNP position
NEL

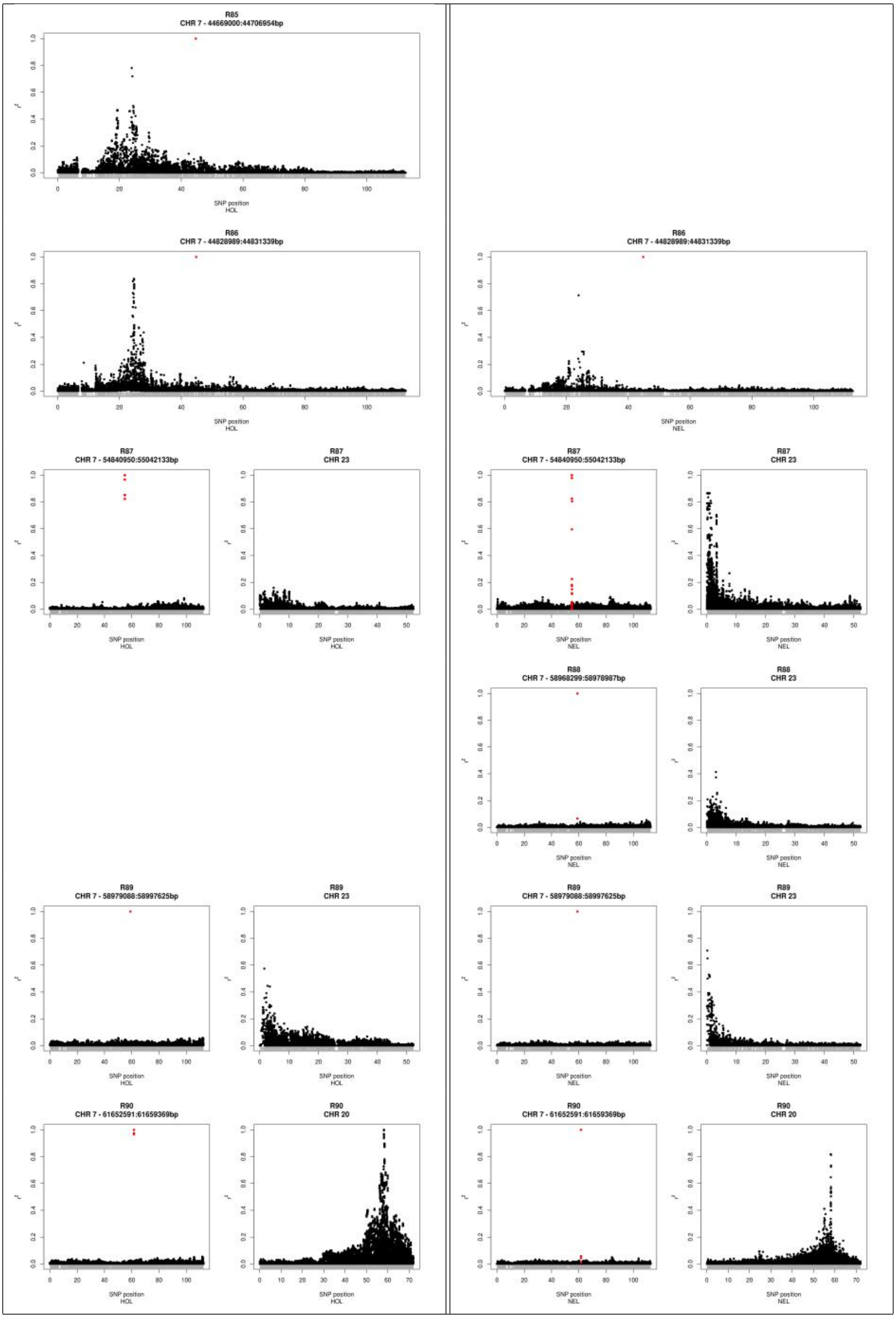
R85
CHR 7 - 44669000:44706954bp
R86
CHR 7 - 44828989:44831339bp
R87
CHR 7 - 54840950:55042133bp
R87
CHR 23
R88
CHR 7 - 58968299:58978987bp
R88
CHR 23
R89
CHR 7 - 58979088:58997625bp
R89
CHR 23
R90
CHR 7 - 61652591:61659369bp
R90
CHR 20
SNP position
HOL
NEL

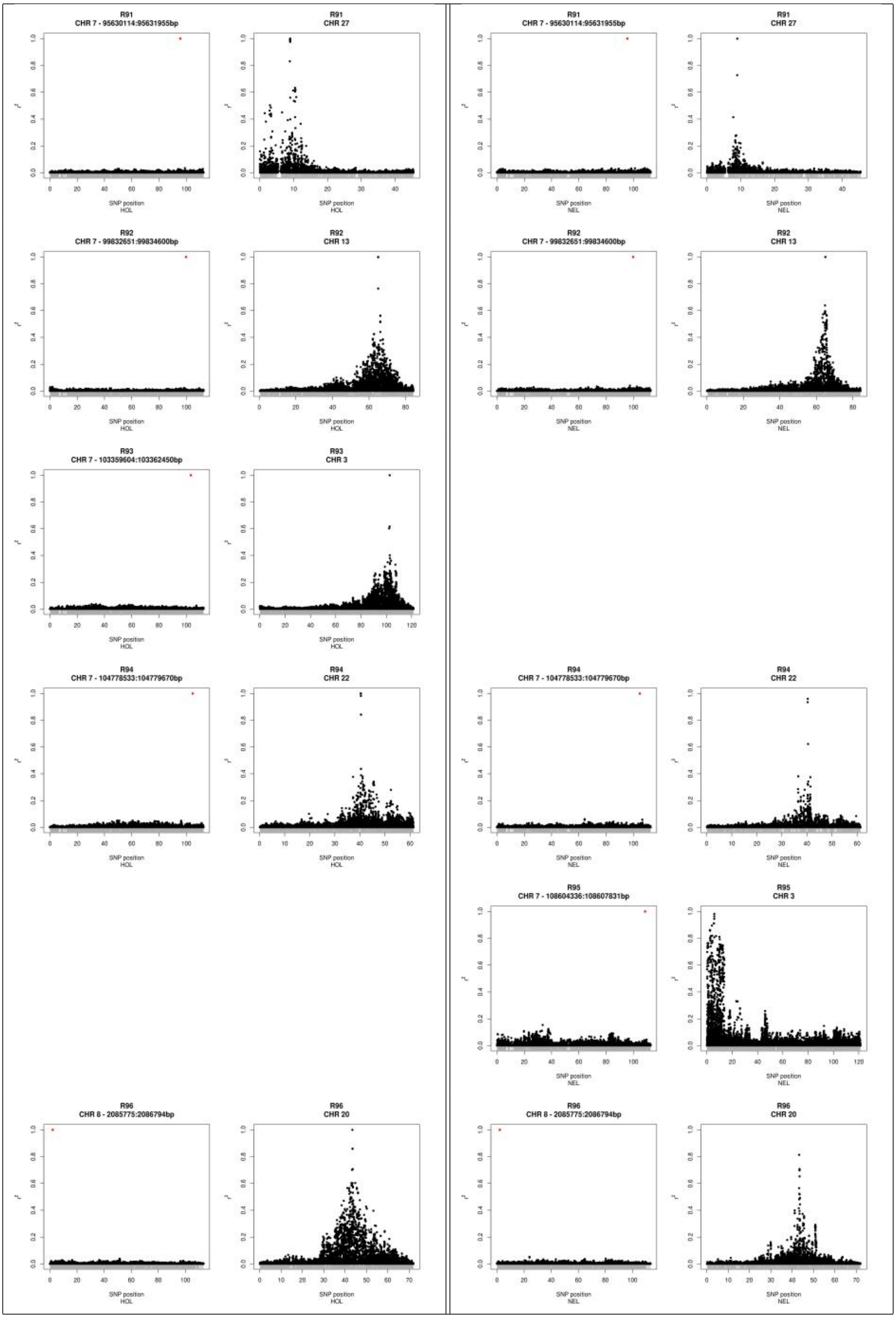
R91
CHR 7 - 95630114:95631955bp
R91
CHR 27
R91
CHR 7 - 95630114:95631955bp
R91
CHR 27
R92
CHR 7 - 99832651:99834600bp
R92
CHR 13
R92
CHR 7 - 99832651:99834600bp
R92
CHR 13
R93
CHR 7 - 103359604:103362450bp
R93
CHR 3
R94
CHR 7 - 104778533:104779670bp
R94
CHR 22
R94
CHR 7 - 104778533:104779670bp
R94
CHR 22
R95
CHR 7 - 108604336:108607831bp
R95
CHR 3
R96
CHR 8 - 2085775:2086794bp
R96
CHR 20
R96
CHR 8 - 2085775:2086794bp
R96
CHR 20
SNP position
HOL
SNP position
NEL

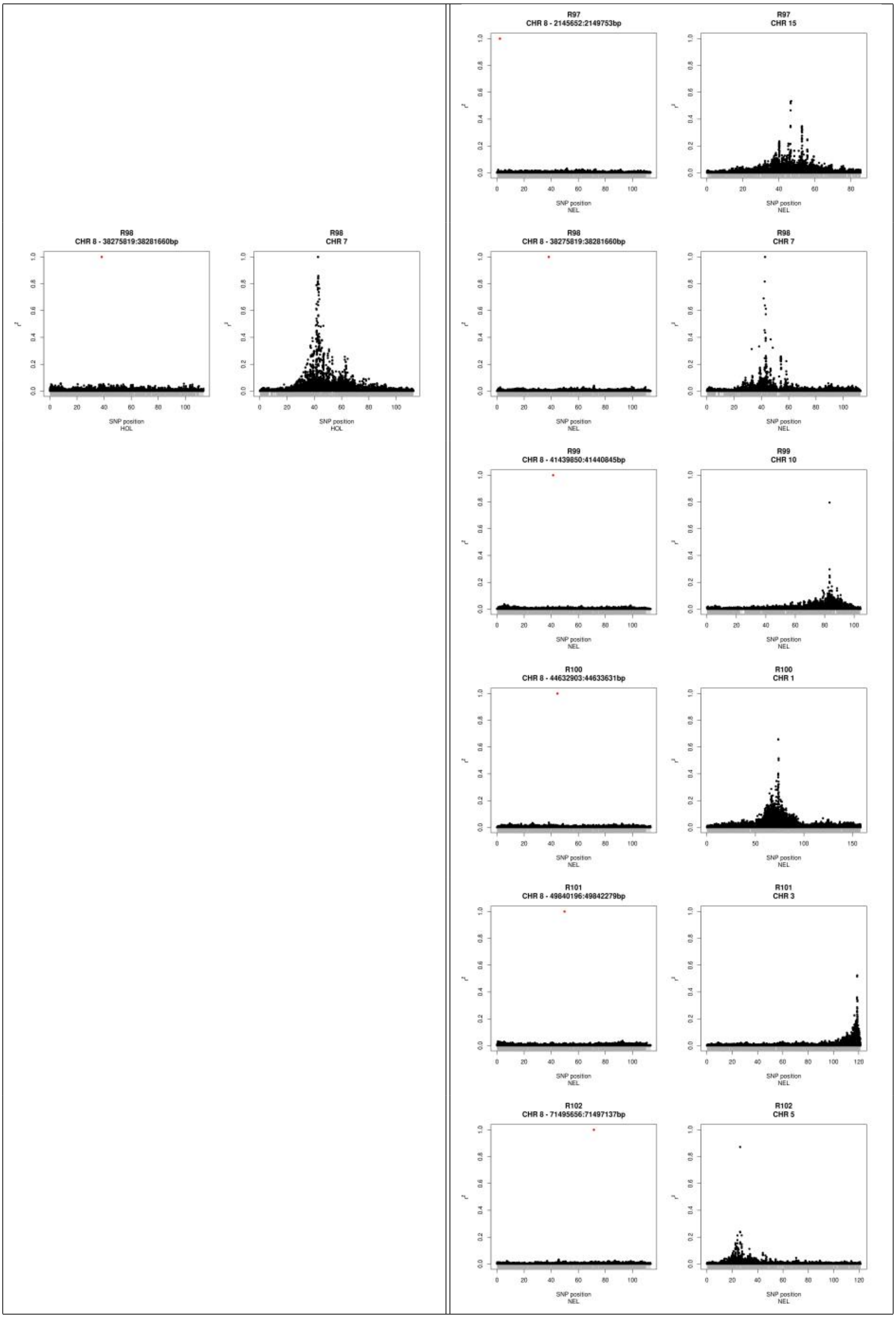
R98
CHR 8 - 38275819:38281660bp
R98
CHR 7
SNP position
HOL
R97
CHR 8 - 2145652:2149753bp
R97
CHR 15
R98
CHR 8 - 38275819:38281660bp
R98
CHR 7
R99
CHR 8 - 41439850:41440845bp
R99
CHR 10
R100
CHR 8 - 44632903:44633631bp
R100
CHR 1
R101
CHR 8 - 49840196:49842279bp
R101
CHR 3
R102
CHR 8 - 71495656:71497137bp
R102
CHR 5
SNP position
NEL
r²

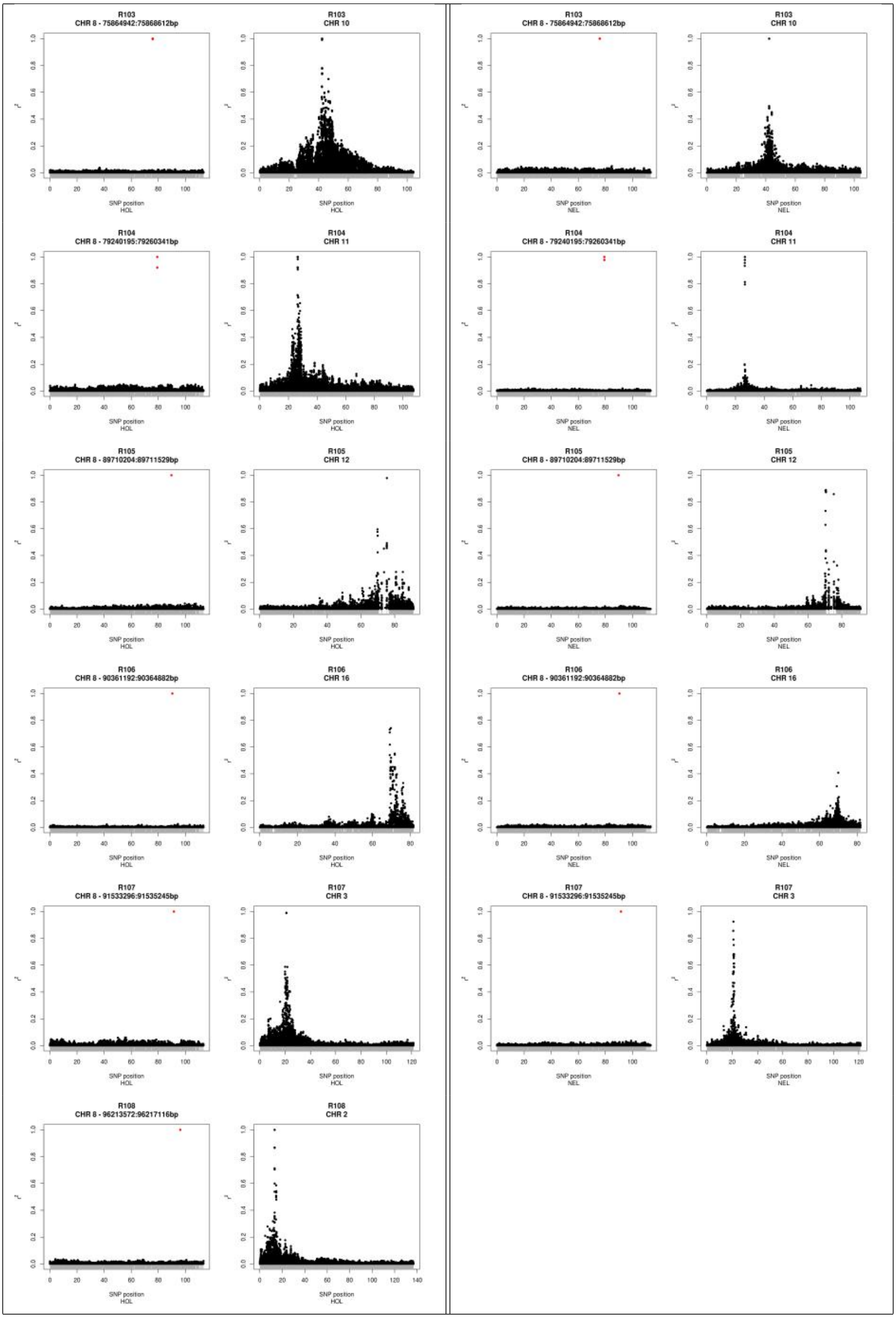
R103
CHR 8 - 75864942:75868612bp
R103
CHR 10
R103
CHR 8 - 75864942:75868612bp
R103
CHR 10
R104
CHR 8 - 79240195:79260341bp
R104
CHR 11
R104
CHR 8 - 79240195:79260341bp
R104
CHR 11
R105
CHR 8 - 89710204:89711529bp
R105
CHR 12
R105
CHR 8 - 89710204:89711529bp
R105
CHR 12
R106
CHR 8 - 90361192:90364882bp
R106
CHR 16
R106
CHR 8 - 90361192:90364882bp
R106
CHR 16
R107
CHR 8 - 91533296:91535245bp
R107
CHR 3
R107
CHR 8 - 91533296:91535245bp
R107
CHR 3
R108
CHR 8 - 96213572:96217116bp
R108
CHR 2
SNP position
HOL
SNP position
NEL

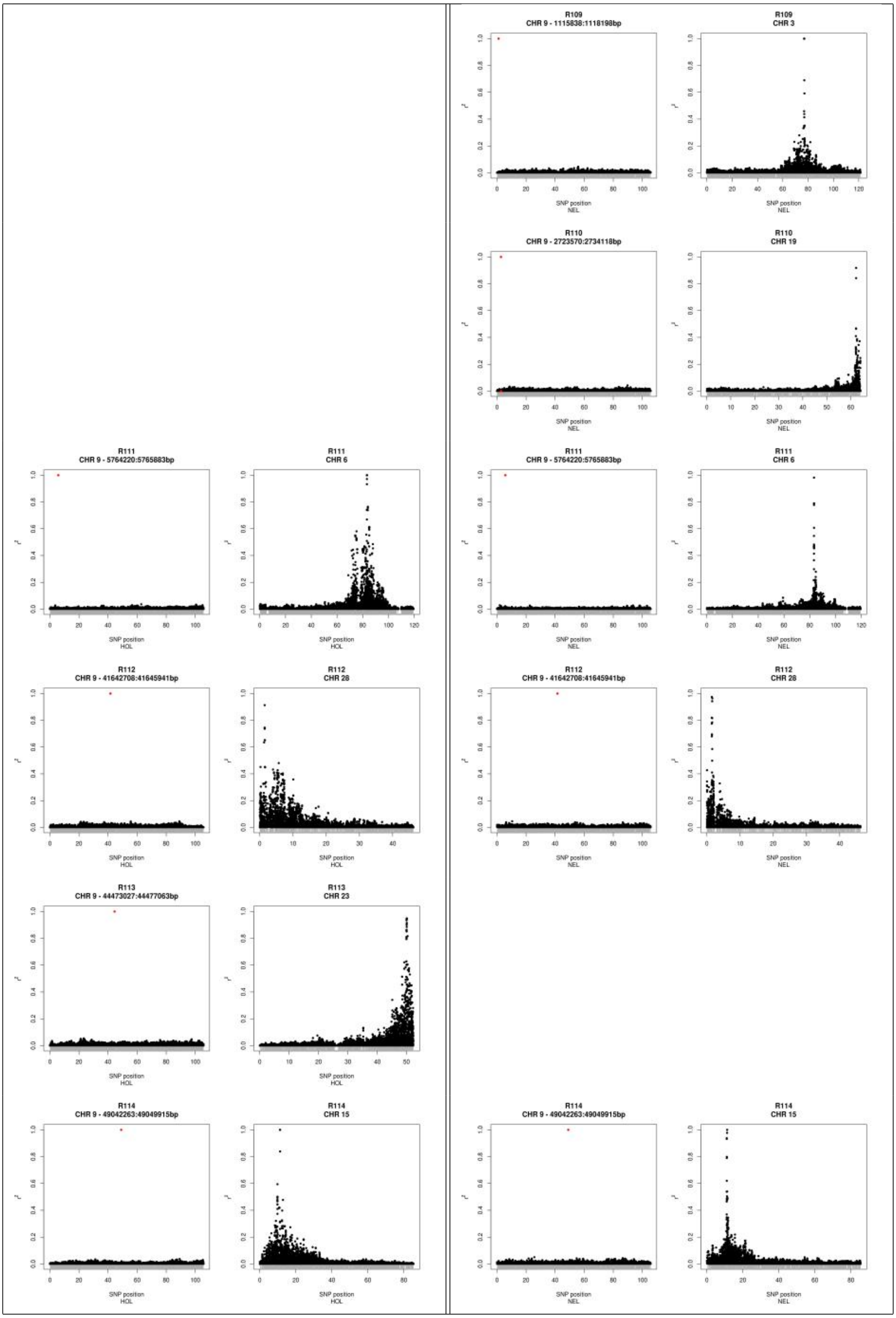
R109
CHR 9 - 1115838:1118198bp
R109
CHR 3
R110
CHR 9 - 2723570:2734118bp
R110
CHR 19
R111
CHR 9 - 5764220:5765883bp
R111
CHR 6
R112
CHR 9 - 41642708:41645941bp
R112
CHR 28
R113
CHR 9 - 44473027:44477063bp
R113
CHR 23
R114
CHR 9 - 49042263:49049915bp
R114
CHR 15
r^2
SNP position
HOL
NEL

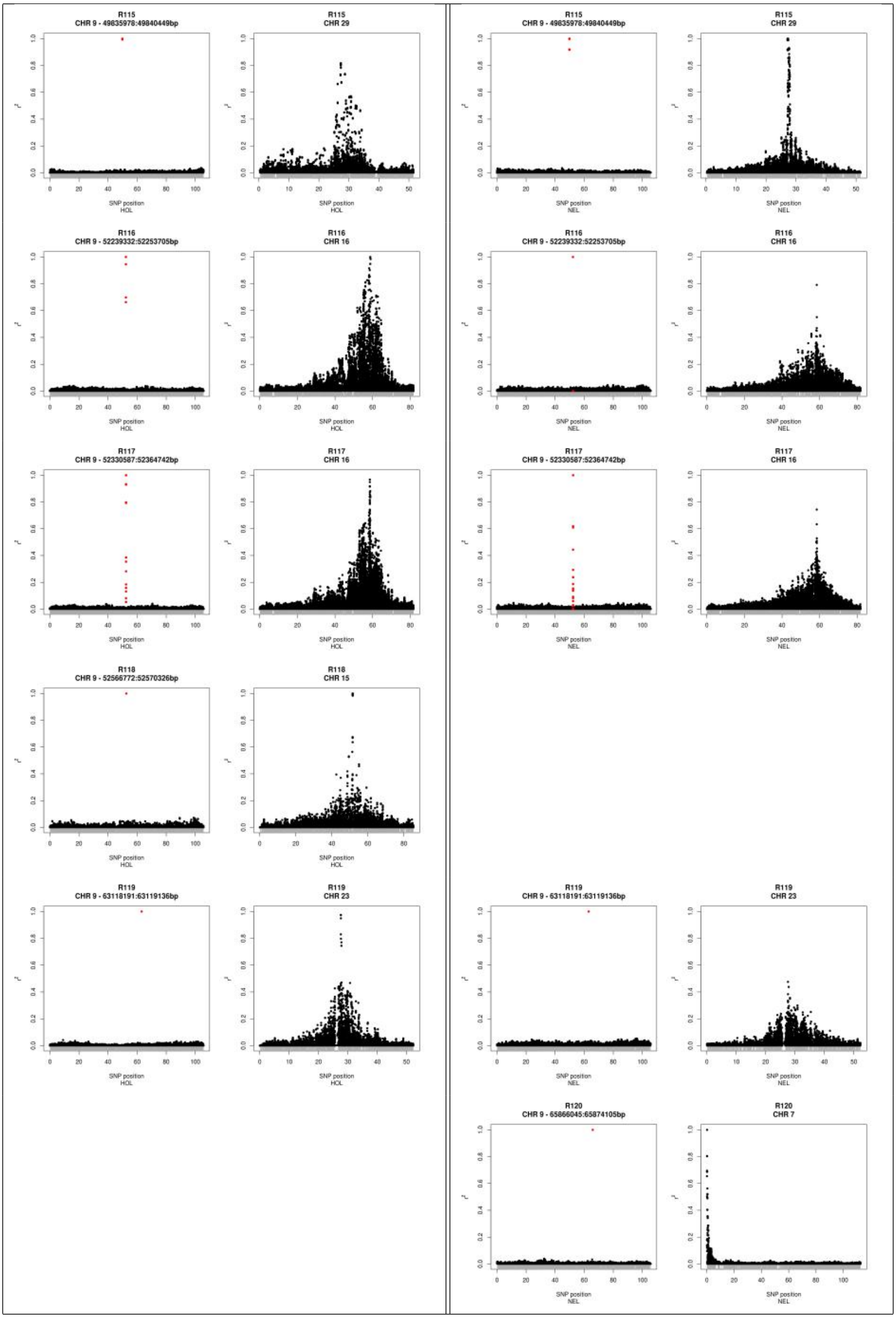
R115
CHR 9 - 49835978:49840449bp
SNP position
HOL
R115
CHR 29
SNP position
HOL
R115
CHR 9 - 49835978:49840449bp
SNP position
NEL
R115
CHR 29
SNP position
NEL
R116
CHR 9 - 52239332:52253705bp
SNP position
HOL
R116
CHR 16
SNP position
HOL
R116
CHR 9 - 52239332:52253705bp
SNP position
NEL
R116
CHR 16
SNP position
NEL
R117
CHR 9 - 52330587:52364742bp
SNP position
HOL
R117
CHR 16
SNP position
HOL
R117
CHR 9 - 52330587:52364742bp
SNP position
NEL
R117
CHR 16
SNP position
NEL
R118
CHR 9 - 52566772:52570326bp
SNP position
HOL
R118
CHR 15
SNP position
HOL
R119
CHR 9 - 63118191:63119136bp
SNP position
HOL
R119
CHR 23
SNP position
HOL
R119
CHR 9 - 63118191:63119136bp
SNP position
NEL
R119
CHR 23
SNP position
NEL
R120
CHR 9 - 65866045:65874105bp
SNP position
NEL
R120
CHR 7
SNP position
NEL

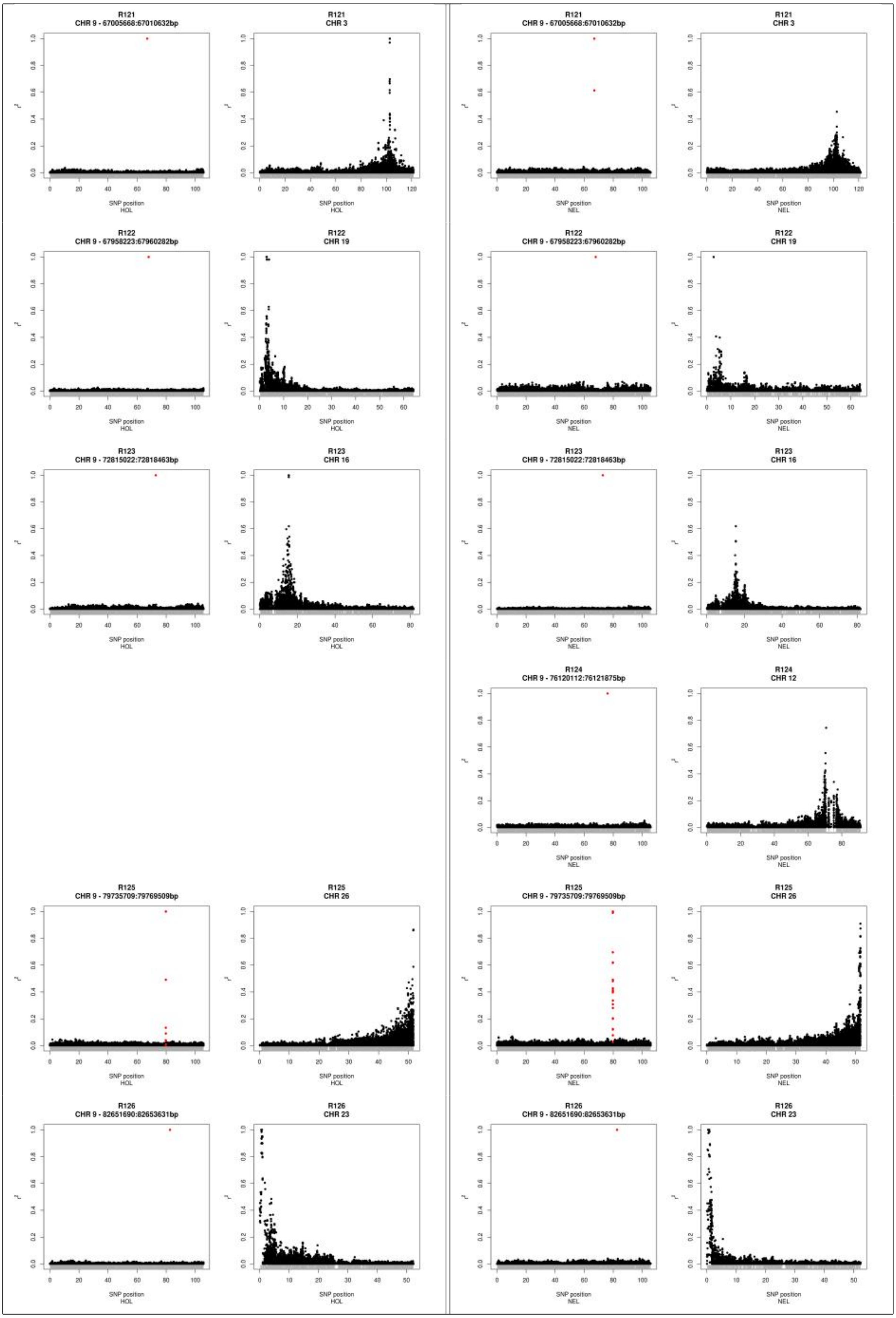
R121
CHR 9 - 67005668:67010632bp
R121
CHR 3
R121
CHR 9 - 67005668:67010632bp
R121
CHR 3
R122
CHR 9 - 67958223:67960282bp
R122
CHR 19
R122
CHR 9 - 67958223:67960282bp
R122
CHR 19
R123
CHR 9 - 72815022:72818463bp
R123
CHR 16
R123
CHR 9 - 72815022:72818463bp
R123
CHR 16
R124
CHR 9 - 76120112:76121875bp
R124
CHR 12
R125
CHR 9 - 79735709:79769509bp
R125
CHR 26
R125
CHR 9 - 79735709:79769509bp
R125
CHR 26
R126
CHR 9 - 82651690:82653631bp
R126
CHR 23
R126
CHR 9 - 82651690:82653631bp
R126
CHR 23
SNP position
HOL
SNP position
NEL
r^2

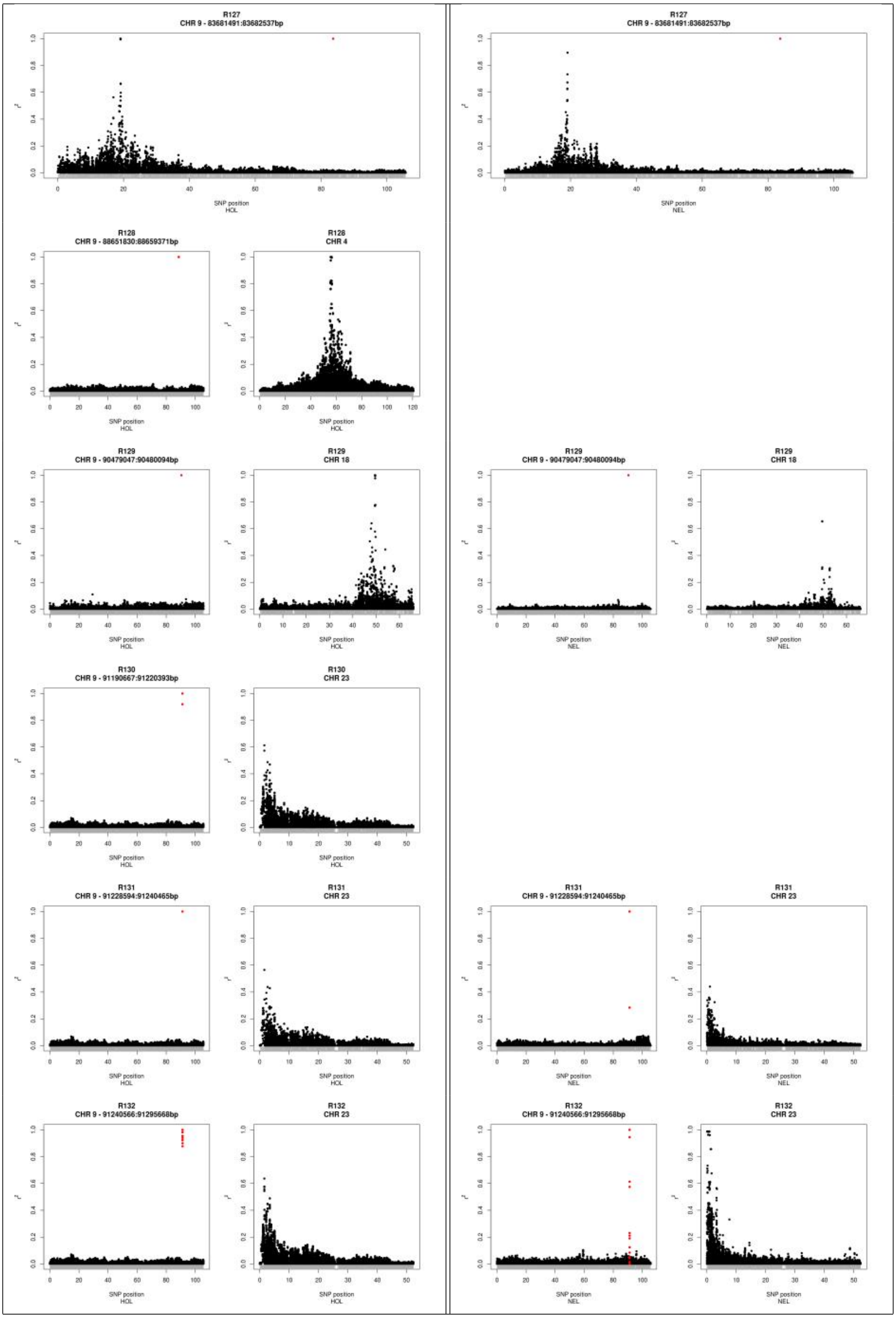
R127
CHR 9 - 83681491:83682537bp
SNP position
HOL
R127
CHR 9 - 83681491:83682537bp
SNP position
NEL
R128
CHR 9 - 88651830:88659371bp
SNP position
HOL
R128
CHR 4
SNP position
HOL
R129
CHR 9 - 90479047:90480094bp
SNP position
HOL
R129
CHR 18
SNP position
HOL
R129
CHR 9 - 90479047:90480094bp
SNP position
NEL
R129
CHR 18
SNP position
NEL
R130
CHR 9 - 91190667:91220393bp
SNP position
HOL
R130
CHR 23
SNP position
HOL
R131
CHR 9 - 91228594:91240465bp
SNP position
HOL
R131
CHR 23
SNP position
HOL
R131
CHR 9 - 91228594:91240465bp
SNP position
NEL
R131
CHR 23
SNP position
NEL
R132
CHR 9 - 91240566:91295668bp
SNP position
HOL
R132
CHR 23
SNP position
HOL
R132
CHR 9 - 91240566:91295668bp
SNP position
NEL
R132
CHR 23
SNP position
NEL

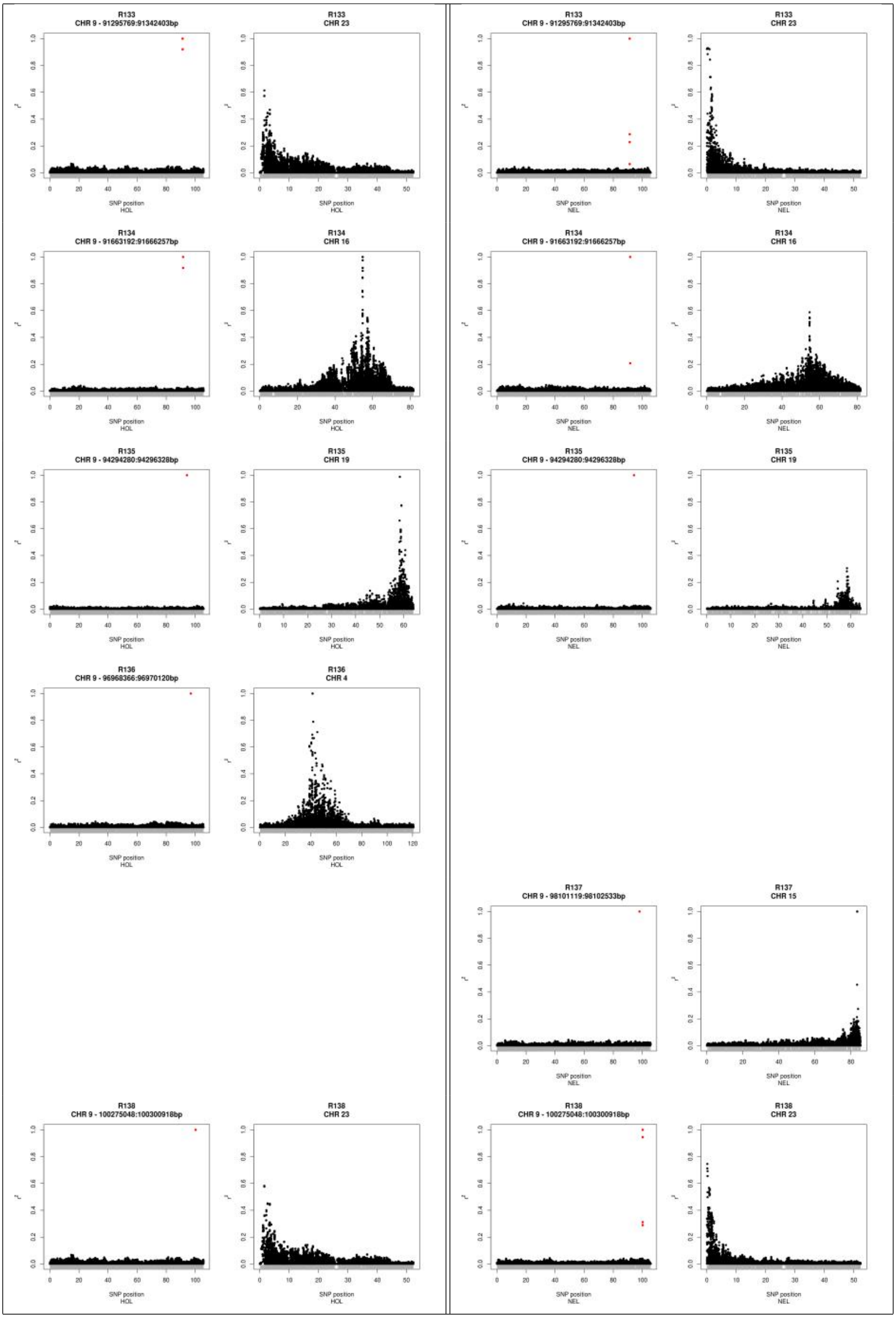
R133
CHR 9 - 91295769:91342403bp
R133
CHR 23
R133
CHR 9 - 91295769:91342403bp
R133
CHR 23
R134
CHR 9 - 91663192:91666257bp
R134
CHR 16
R134
CHR 9 - 91663192:91666257bp
R134
CHR 16
R135
CHR 9 - 94294280:94296328bp
R135
CHR 19
R135
CHR 9 - 94294280:94296328bp
R135
CHR 19
R136
CHR 9 - 96968366:96970120bp
R136
CHR 4
R137
CHR 9 - 98101119:98102533bp
R137
CHR 15
R138
CHR 9 - 100275048:100300918bp
R138
CHR 23
R138
CHR 9 - 100275048:100300918bp
R138
CHR 23
SNP position
HOL
NEL

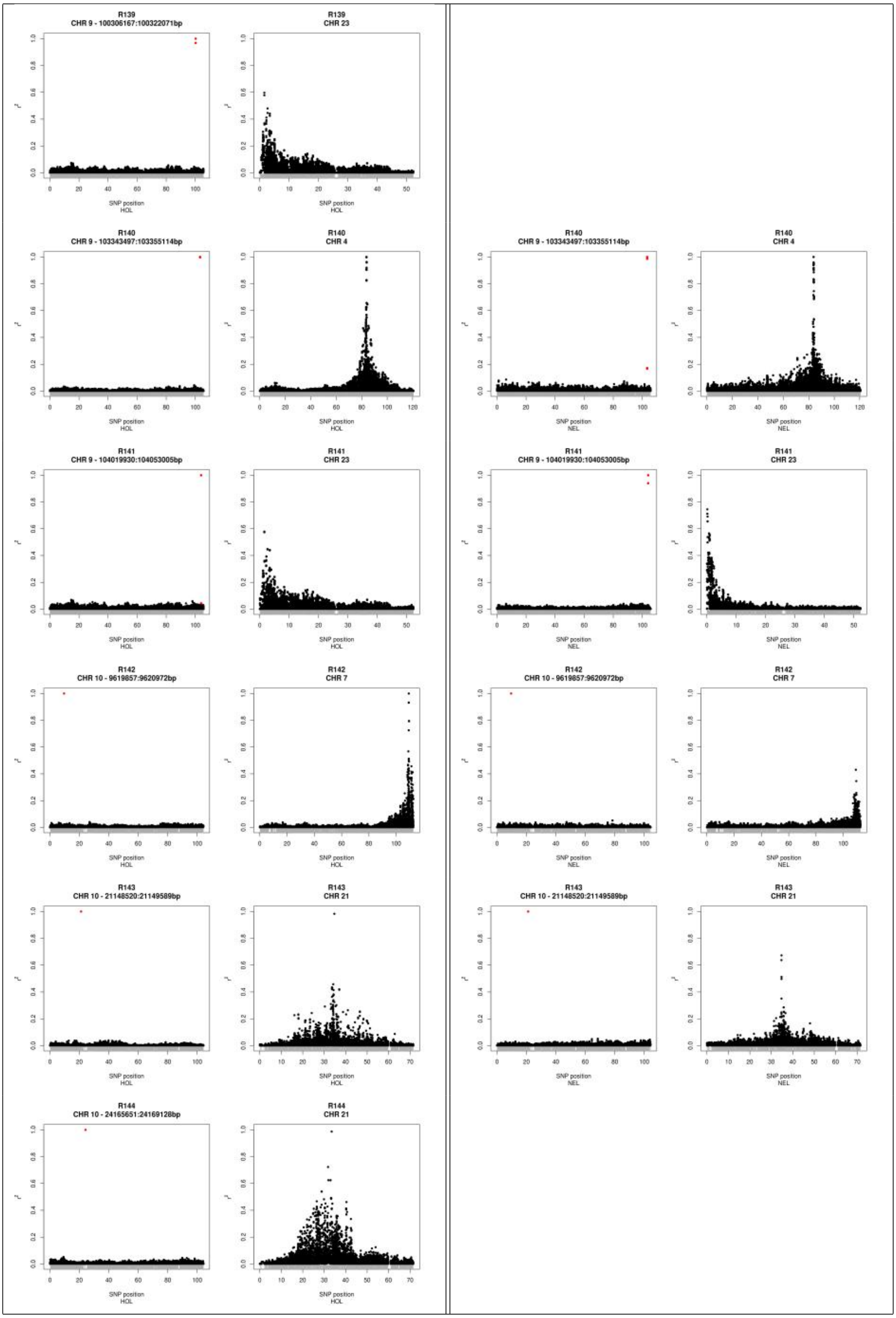
R139
CHR 9 - 100306167:100322071bp
SNP position
HOL
R139
CHR 23
SNP position
HOL
R140
CHR 9 - 103343497:103355114bp
SNP position
HOL
R140
CHR 4
SNP position
HOL
R140
CHR 9 - 103343497:103355114bp
SNP position
NEL
R140
CHR 4
SNP position
NEL
R141
CHR 9 - 104019930:104053005bp
SNP position
HOL
R141
CHR 23
SNP position
HOL
R141
CHR 9 - 104019930:104053005bp
SNP position
NEL
R141
CHR 23
SNP position
NEL
R142
CHR 10 - 9619857:9620972bp
SNP position
HOL
R142
CHR 7
SNP position
HOL
R142
CHR 10 - 9619857:9620972bp
SNP position
NEL
R142
CHR 7
SNP position
NEL
R143
CHR 10 - 21148520:21149589bp
SNP position
HOL
R143
CHR 21
SNP position
HOL
R143
CHR 10 - 21148520:21149589bp
SNP position
NEL
R143
CHR 21
SNP position
NEL
R144
CHR 10 - 24165651:24169128bp
SNP position
HOL
R144
CHR 21
SNP position
HOL

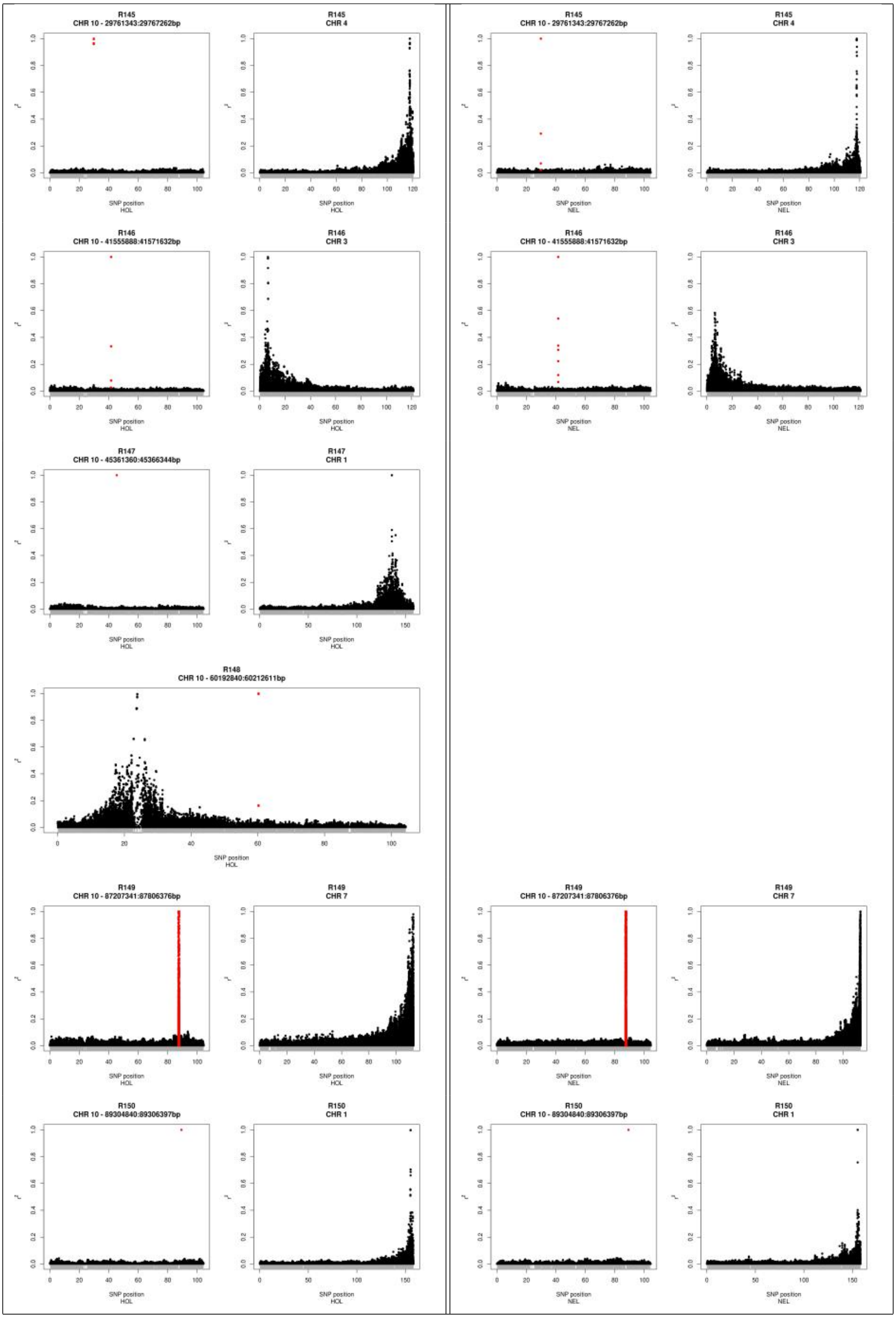
R145
CHR 10 - 29761343:29767262bp
SNP position
HOL
R145
CHR 4
SNP position
HOL
R145
CHR 10 - 29761343:29767262bp
SNP position
NEL
R145
CHR 4
SNP position
NEL
R146
CHR 10 - 41555888:41571632bp
SNP position
HOL
R146
CHR 3
SNP position
HOL
R146
CHR 10 - 41555888:41571632bp
SNP position
NEL
R146
CHR 3
SNP position
NEL
R147
CHR 10 - 45361360:45366344bp
SNP position
HOL
R147
CHR 1
SNP position
HOL
R148
CHR 10 - 60192840:60212611bp
SNP position
HOL
R149
CHR 10 - 87207341:87806376bp
SNP position
HOL
R149
CHR 7
SNP position
HOL
R149
CHR 10 - 87207341:87806376bp
SNP position
NEL
R149
CHR 7
SNP position
NEL
R150
CHR 10 - 89304840:89306397bp
SNP position
HOL
R150
CHR 1
SNP position
HOL
R150
CHR 10 - 89304840:89306397bp
SNP position
NEL
R150
CHR 1
SNP position
NEL

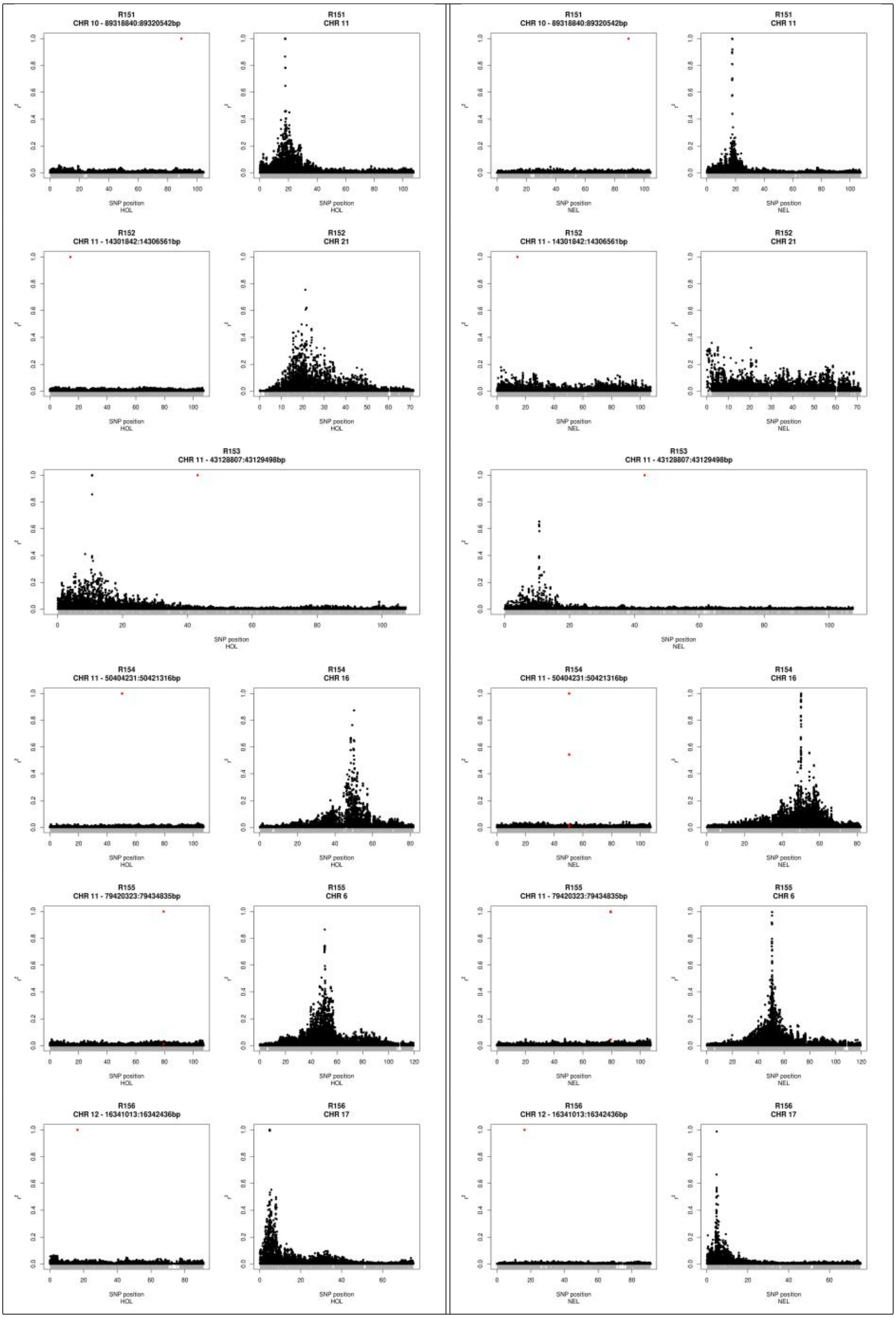
R151
CHR 10 - 89318840:89320542bp
SNP position
HOL
R151
CHR 11
SNP position
HOL
R152
CHR 11 - 14301842:14306561bp
SNP position
HOL
R152
CHR 21
SNP position
HOL
R153
CHR 11 - 43128807:43129498bp
SNP position
HOL
R154
CHR 11 - 50404231:50421316bp
SNP position
HOL
R154
CHR 16
SNP position
HOL
R155
CHR 11 - 79420323:79434835bp
SNP position
HOL
R155
CHR 6
SNP position
HOL
R156
CHR 12 - 16341013:16342436bp
SNP position
HOL
R156
CHR 17
SNP position
HOL
R151
CHR 10 - 89318840:89320542bp
SNP position
NEL
R151
CHR 11
SNP position
NEL
R152
CHR 11 - 14301842:14306561bp
SNP position
NEL
R152
CHR 21
SNP position
NEL
R153
CHR 11 - 43128807:43129498bp
SNP position
NEL
R154
CHR 11 - 50404231:50421316bp
SNP position
NEL
R154
CHR 16
SNP position
NEL
R155
CHR 11 - 79420323:79434835bp
SNP position
NEL
R155
CHR 6
SNP position
NEL
R156
CHR 12 - 16341013:16342436bp
SNP position
NEL
R156
CHR 17
SNP position
NEL

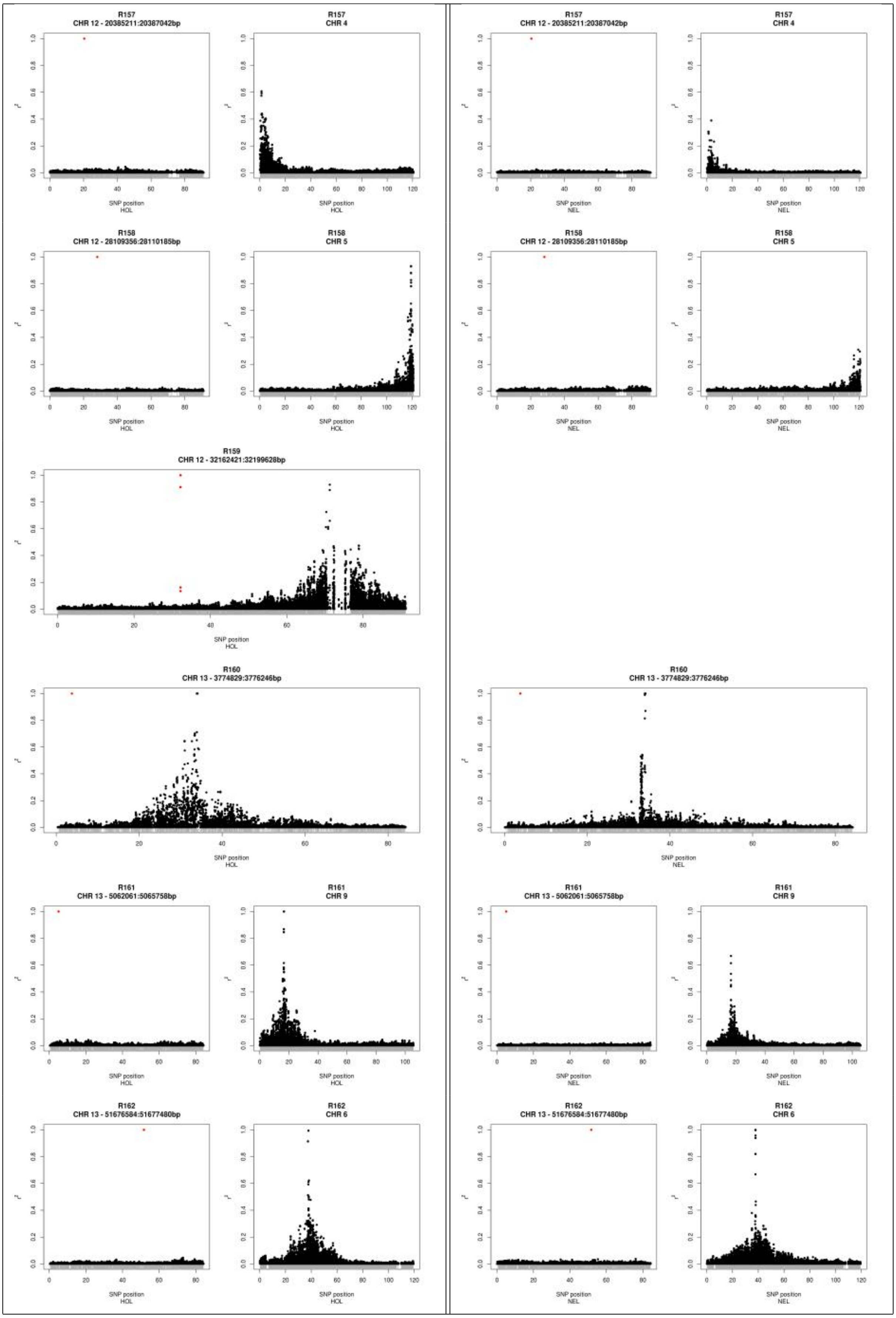
R157
CHR 12 - 20385211:20387042bp
R157
CHR 4
R158
CHR 12 - 28109356:28110185bp
R158
CHR 5
R159
CHR 12 - 32162421:32199628bp
R160
CHR 13 - 3774829:3776246bp
R161
CHR 13 - 5062061:5065758bp
R161
CHR 9
R162
CHR 13 - 51676584:51677480bp
R162
CHR 6
SNP position
HOL
NEL
r^2

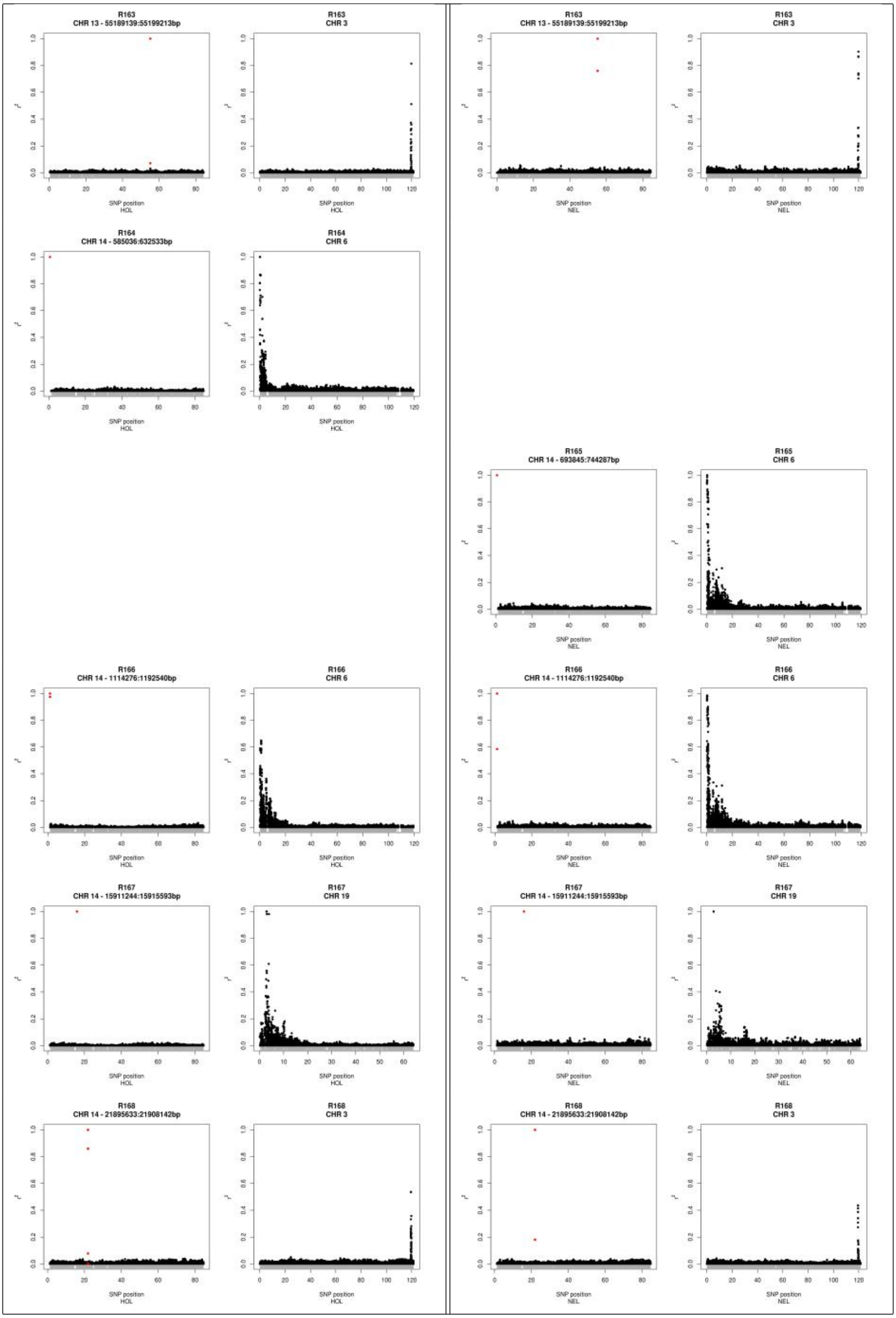
R163
CHR 13 - 55189139:55199213bp
R163
CHR 3
R164
CHR 14 - 585036:632533bp
R164
CHR 6
R165
CHR 14 - 693845:744287bp
R165
CHR 6
R166
CHR 14 - 1114276:1192540bp
R166
CHR 6
R167
CHR 14 - 15911244:15915593bp
R167
CHR 19
R168
CHR 14 - 21895633:21908142bp
R168
CHR 3
SNP position
HOL
NEL
r²

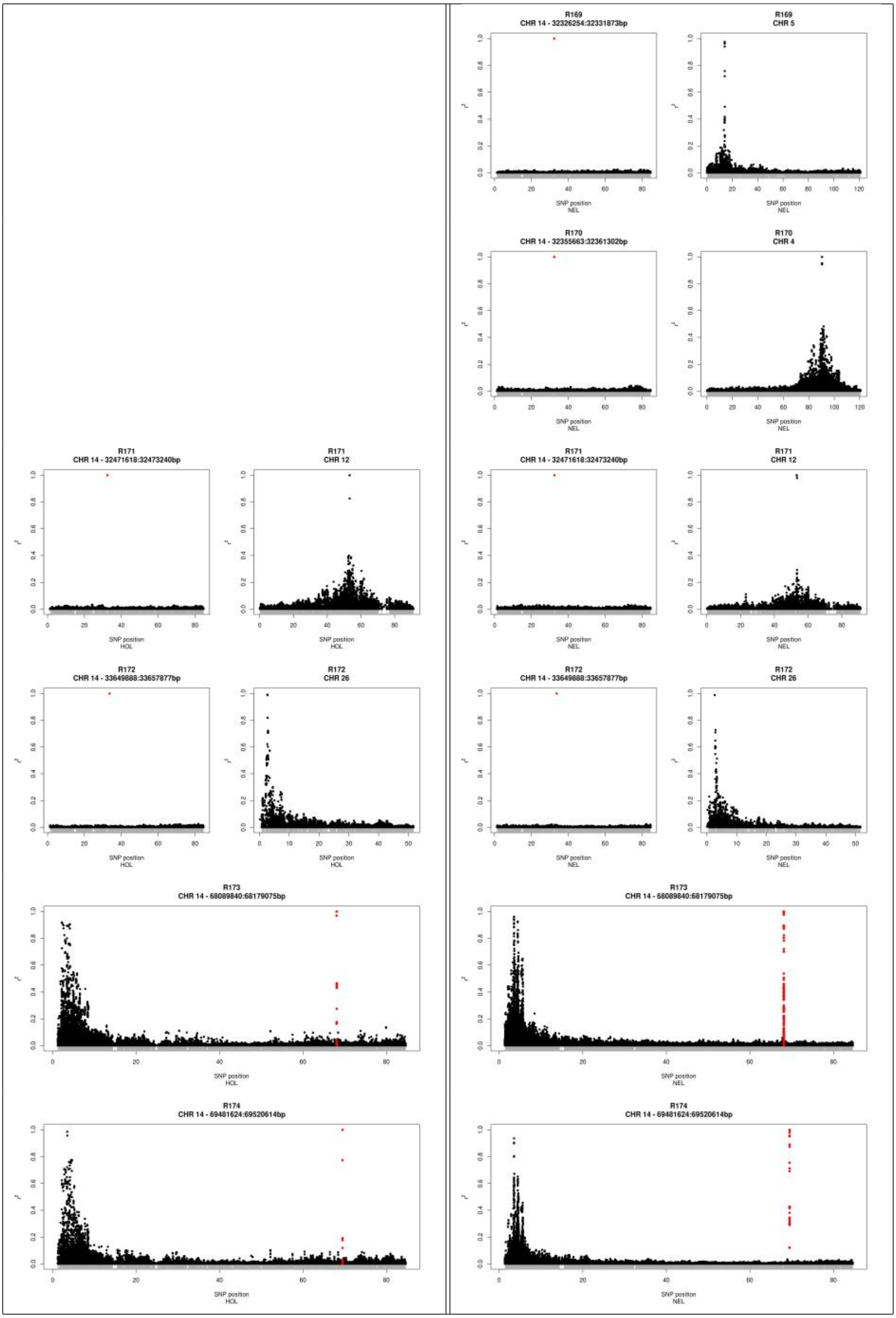
R169
CHR 14 - 32326254:32331873bp
R169
CHR 5
R170
CHR 14 - 32355663:32361302bp
R170
CHR 4
R171
CHR 14 - 32471618:32473240bp
R171
CHR 12
R172
CHR 14 - 33649888:33657877bp
R172
CHR 26
R173
CHR 14 - 68089840:68179075bp
R174
CHR 14 - 69481624:69520614bp
r²
SNP position
HOL
NEL

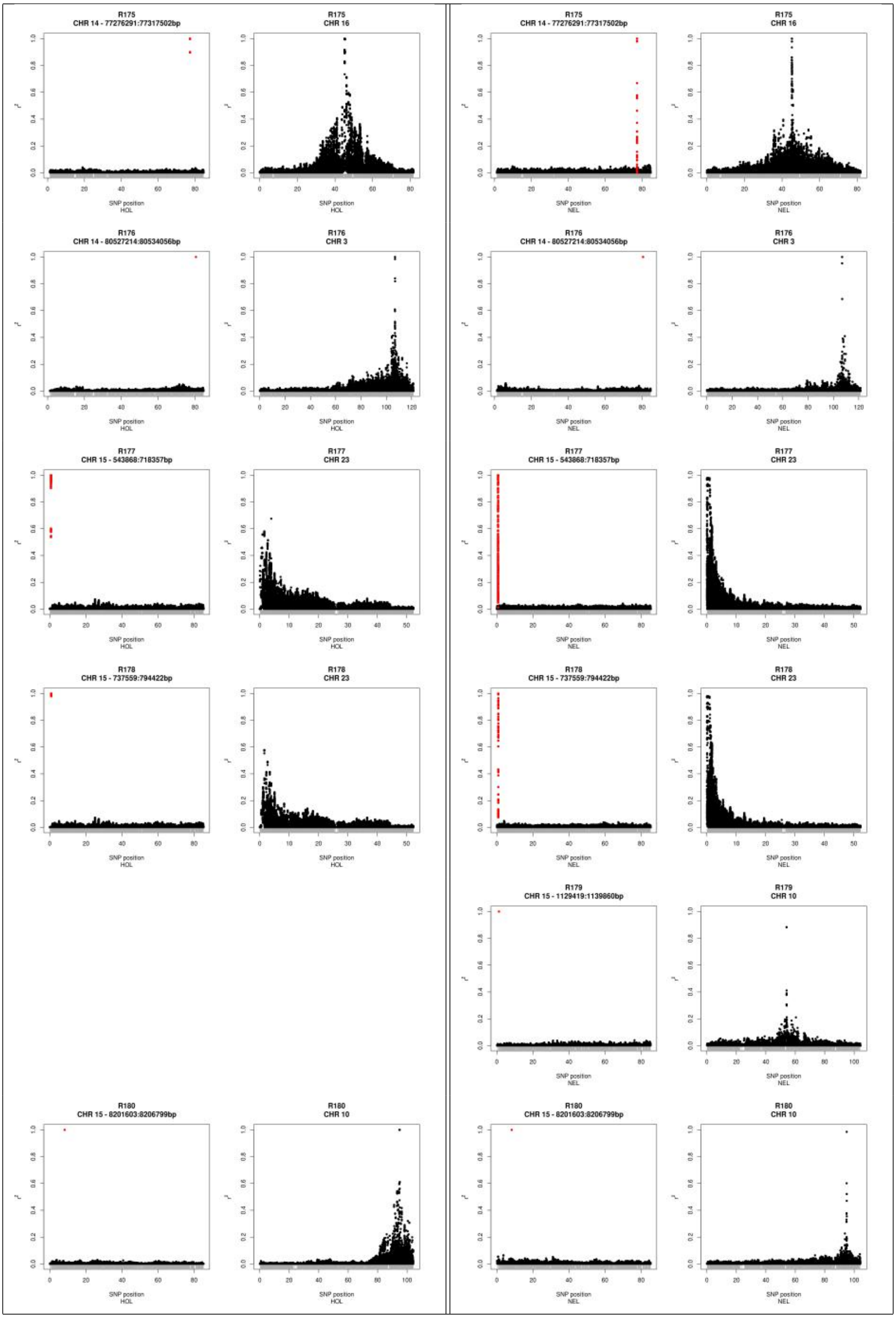
R175
CHR 14 - 77276291:77317502bp
R175
CHR 16
R175
CHR 14 - 77276291:77317502bp
R175
CHR 16
R176
CHR 14 - 80527214:80534056bp
R176
CHR 3
R176
CHR 14 - 80527214:80534056bp
R176
CHR 3
R177
CHR 15 - 543868:718357bp
R177
CHR 23
R177
CHR 15 - 543868:718357bp
R177
CHR 23
R178
CHR 15 - 737559:794422bp
R178
CHR 23
R178
CHR 15 - 737559:794422bp
R178
CHR 23
R179
CHR 15 - 1129419:1139860bp
R179
CHR 10
R180
CHR 15 - 8201603:8206799bp
R180
CHR 10
R180
CHR 15 - 8201603:8206799bp
R180
CHR 10
SNP position
HOL
SNP position
NEL

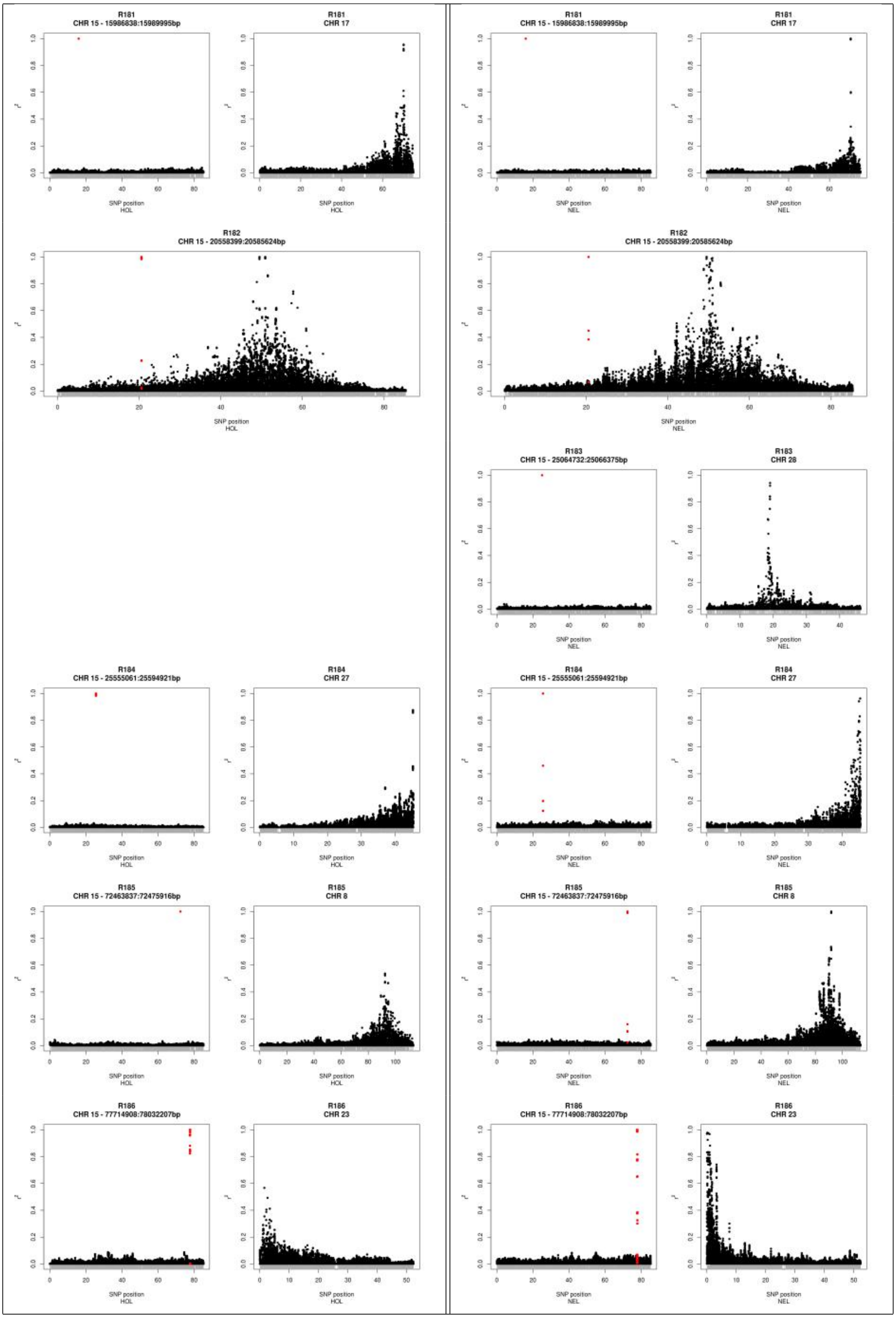
R181
CHR 15 - 15986838:15989995bp
R181
CHR 17
R182
CHR 15 - 20558399:20585624bp
R183
CHR 15 - 25064732:25066375bp
R183
CHR 28
R184
CHR 15 - 25555061:25594921bp
R184
CHR 27
R185
CHR 15 - 72463837:72475916bp
R185
CHR 8
R186
CHR 15 - 77714908:78032207bp
R186
CHR 23
SNP position
HOL
NEL

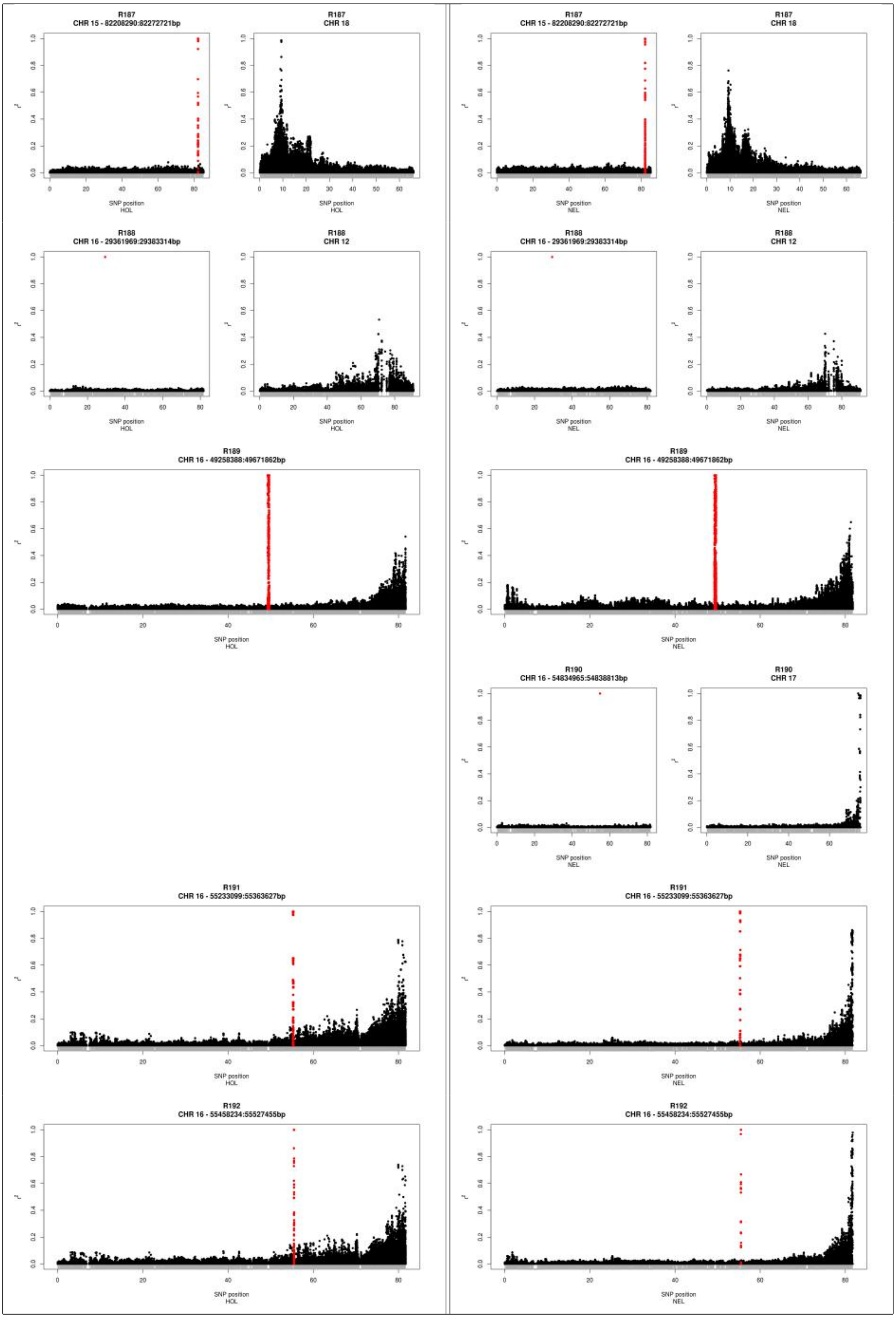
R187
CHR 15 - 82208290:82272721bp
R187
CHR 18
R188
CHR 16 - 29361969:29383314bp
R188
CHR 12
R189
CHR 16 - 49258388:49671862bp
R190
CHR 16 - 54834965:54838813bp
R190
CHR 17
R191
CHR 16 - 55233099:55363627bp
R192
CHR 16 - 55458234:55527455bp
SNP position
HOL
NEL

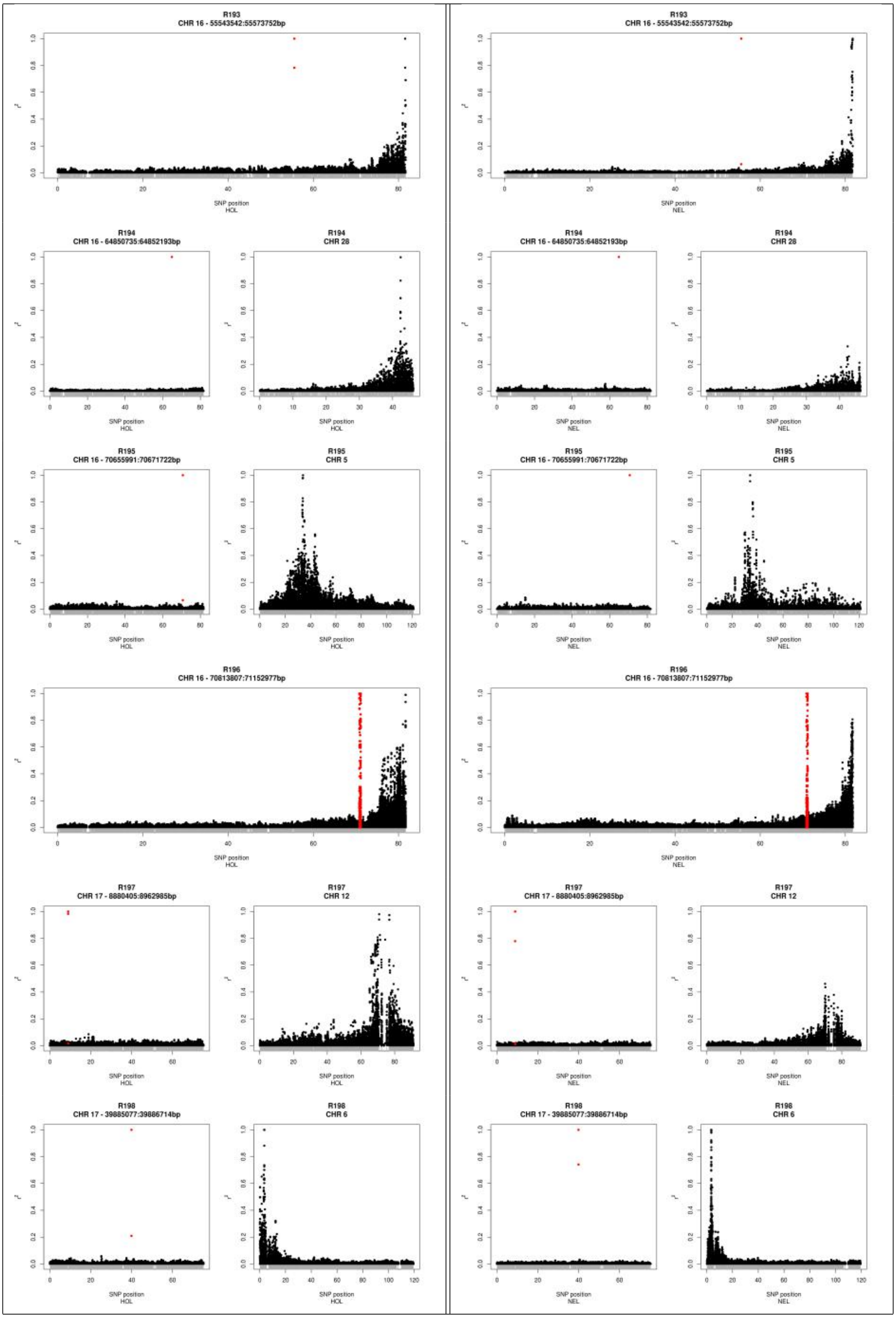
R193
CHR 16 - 55543542:55573752bp
SNP position
HOL
R193
CHR 16 - 55543542:55573752bp
SNP position
NEL
R194
CHR 16 - 64850735:64852193bp
SNP position
HOL
R194
CHR 28
SNP position
HOL
R194
CHR 16 - 64850735:64852193bp
SNP position
NEL
R194
CHR 28
SNP position
NEL
R195
CHR 16 - 70655991:70671722bp
SNP position
HOL
R195
CHR 5
SNP position
HOL
R195
CHR 16 - 70655991:70671722bp
SNP position
NEL
R195
CHR 5
SNP position
NEL
R196
CHR 16 - 70813807:71152977bp
SNP position
HOL
R196
CHR 16 - 70813807:71152977bp
SNP position
NEL
R197
CHR 17 - 8880405:8962985bp
SNP position
HOL
R197
CHR 12
SNP position
HOL
R197
CHR 17 - 8880405:8962985bp
SNP position
NEL
R197
CHR 12
SNP position
NEL
R198
CHR 17 - 39885077:39886714bp
SNP position
HOL
R198
CHR 6
SNP position
HOL
R198
CHR 17 - 39885077:39886714bp
SNP position
NEL
R198
CHR 6
SNP position
NEL

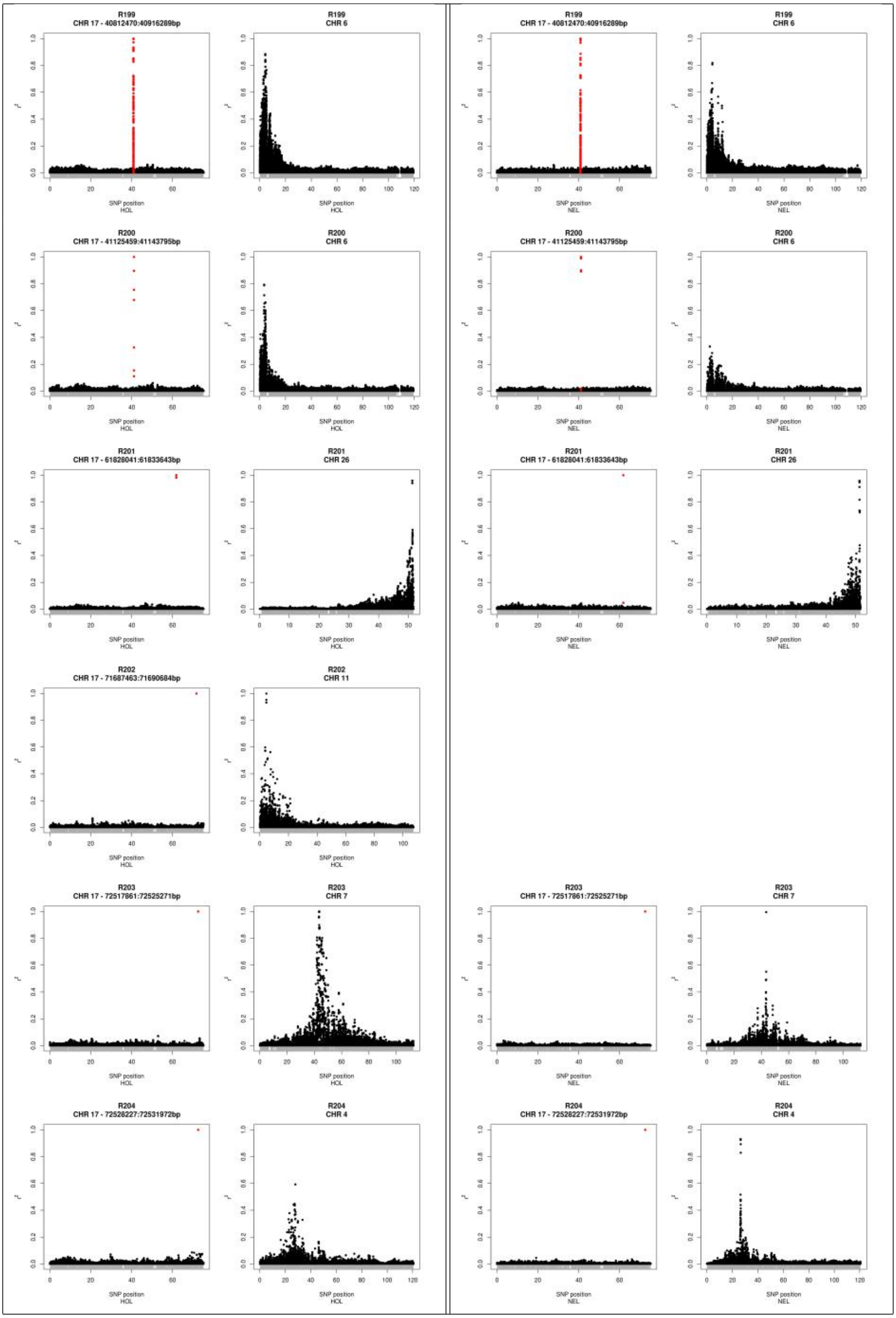
R199
CHR 17 - 40812470:40916289bp
R199
CHR 6
R199
CHR 17 - 40812470:40916289bp
R199
CHR 6
R200
CHR 17 - 41125459:41143795bp
R200
CHR 6
R200
CHR 17 - 41125459:41143795bp
R200
CHR 6
R201
CHR 17 - 61828041:61833643bp
R201
CHR 26
R201
CHR 17 - 61828041:61833643bp
R201
CHR 26
R202
CHR 17 - 71687463:71690684bp
R202
CHR 11
R203
CHR 17 - 72517861:72525271bp
R203
CHR 7
R203
CHR 17 - 72517861:72525271bp
R203
CHR 7
R204
CHR 17 - 72528227:72531972bp
R204
CHR 4
R204
CHR 17 - 72528227:72531972bp
R204
CHR 4
r^2
SNP position
HOL
NEL

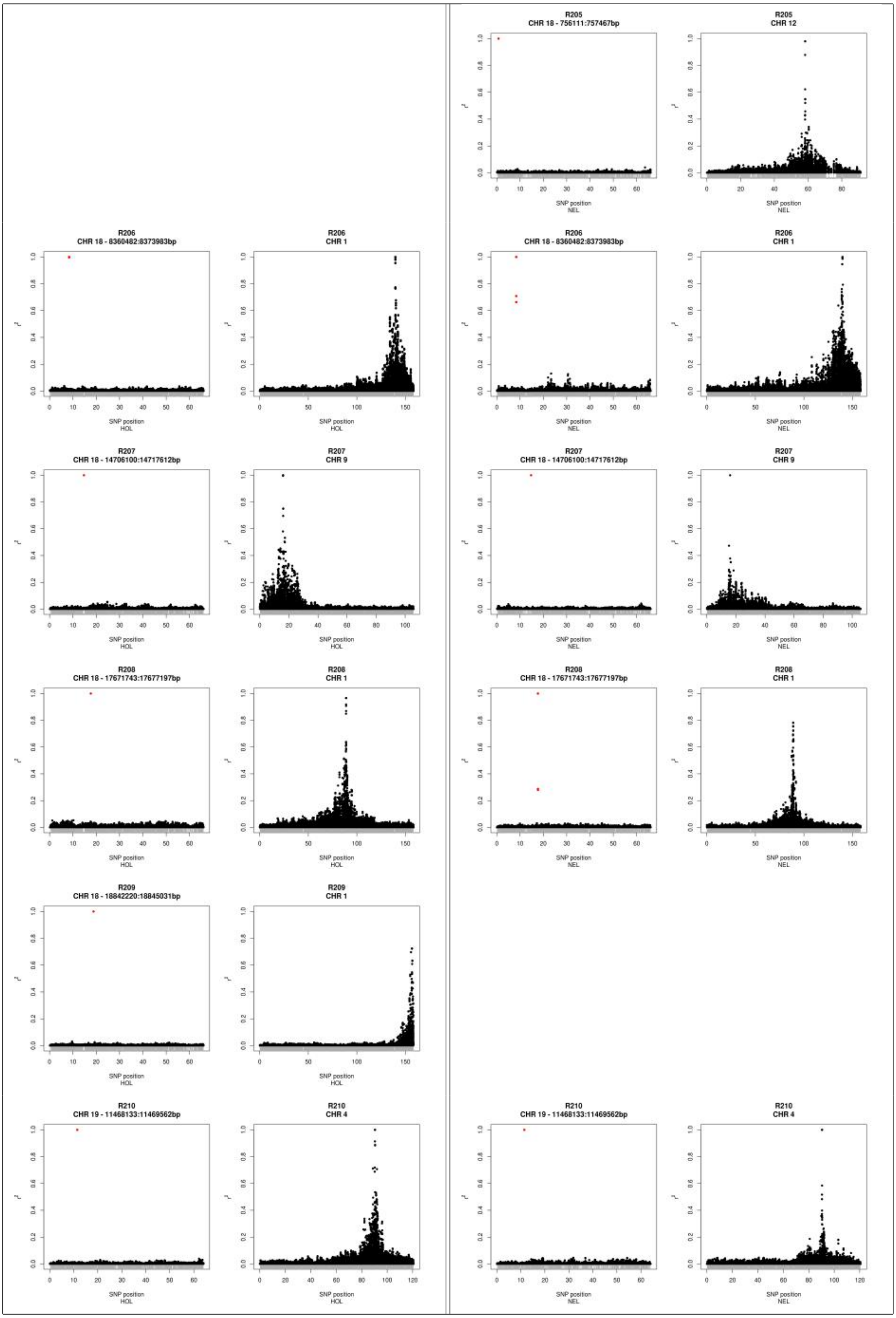
R205
CHR 18 - 756111:757467bp
SNP position
NEL
R205
CHR 12
SNP position
NEL
R206
CHR 18 - 8360482:8373983bp
SNP position
HOL
R206
CHR 1
SNP position
HOL
R206
CHR 18 - 8360482:8373983bp
SNP position
NEL
R206
CHR 1
SNP position
NEL
R207
CHR 18 - 14706100:14717612bp
SNP position
HOL
R207
CHR 9
SNP position
HOL
R207
CHR 18 - 14706100:14717612bp
SNP position
NEL
R207
CHR 9
SNP position
NEL
R208
CHR 18 - 17671743:17677197bp
SNP position
HOL
R208
CHR 1
SNP position
HOL
R208
CHR 18 - 17671743:17677197bp
SNP position
NEL
R208
CHR 1
SNP position
NEL
R209
CHR 18 - 18842220:18845031bp
SNP position
HOL
R209
CHR 1
SNP position
HOL
R210
CHR 19 - 11468133:11469562bp
SNP position
HOL
R210
CHR 4
SNP position
HOL
R210
CHR 19 - 11468133:11469562bp
SNP position
NEL
R210
CHR 4
SNP position
NEL

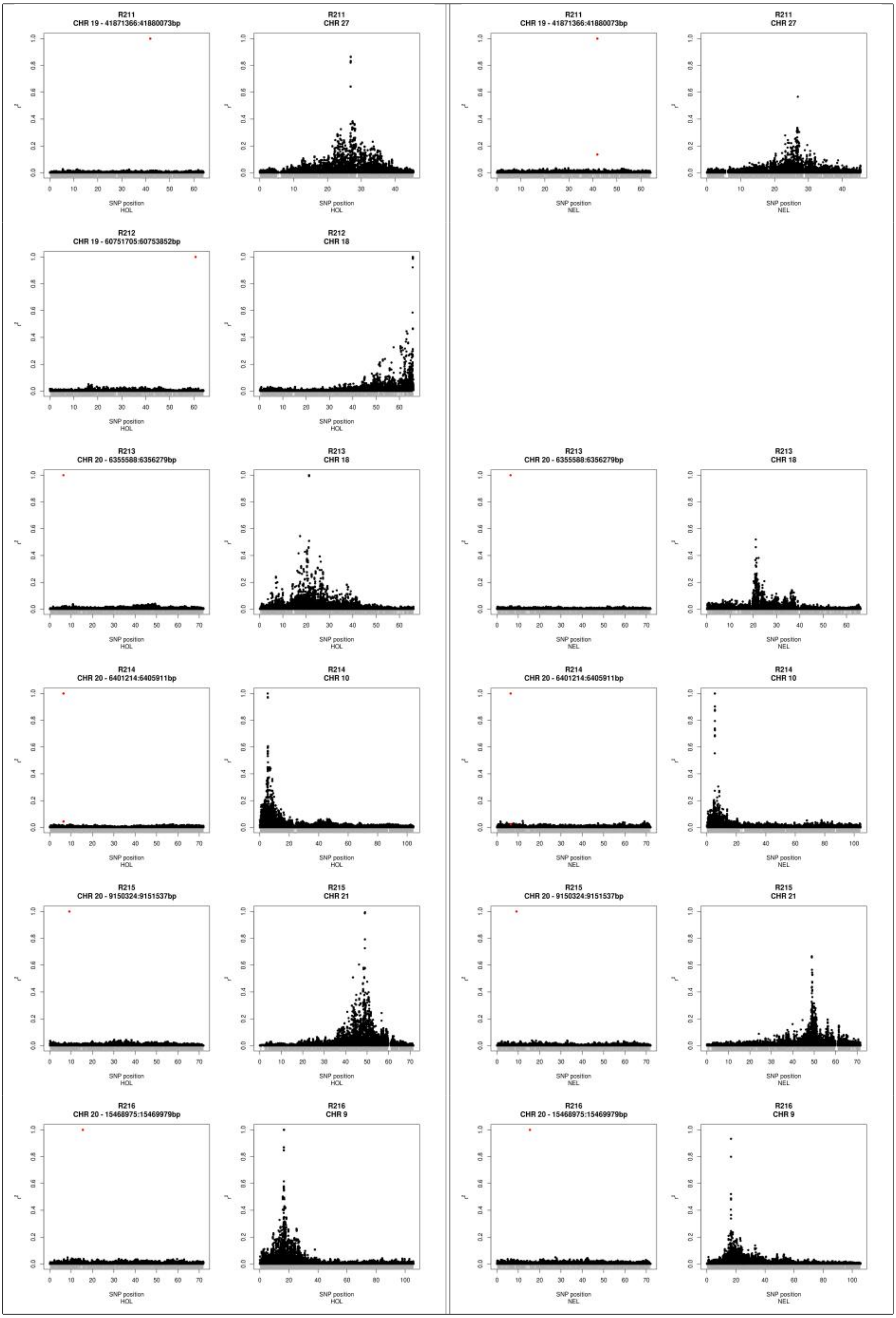
R211
CHR 19 - 41871366:41880073bp
R211
CHR 27
R212
CHR 19 - 60751705:60753852bp
R212
CHR 18
R213
CHR 20 - 6355588:6356279bp
R213
CHR 18
R214
CHR 20 - 6401214:6405911bp
R214
CHR 10
R215
CHR 20 - 9150324:9151537bp
R215
CHR 21
R216
CHR 20 - 15468975:15469979bp
R216
CHR 9
SNP position
HOL
NEL
r^2

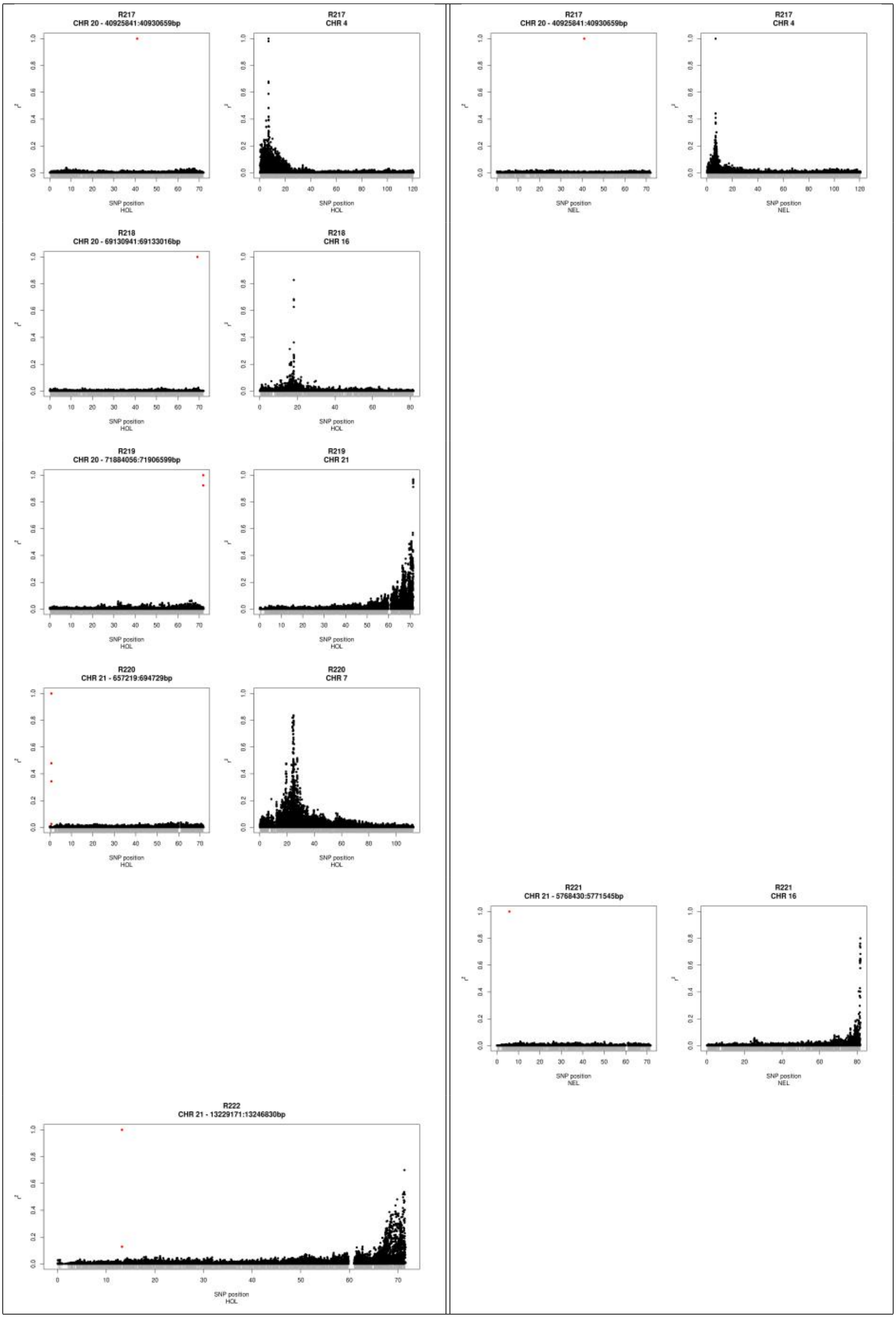
R217
CHR 20 - 40925841:40930659bp
R217
CHR 4
R218
CHR 20 - 69130941:69133016bp
R218
CHR 16
R219
CHR 20 - 71884056:71906599bp
R219
CHR 21
R220
CHR 21 - 657219:694729bp
R220
CHR 7
R222
CHR 21 - 13229171:13246830bp
R217
CHR 20 - 40925841:40930659bp
R217
CHR 4
R221
CHR 21 - 5768430:5771545bp
R221
CHR 16
SNP position
HOL
SNP position
NEL

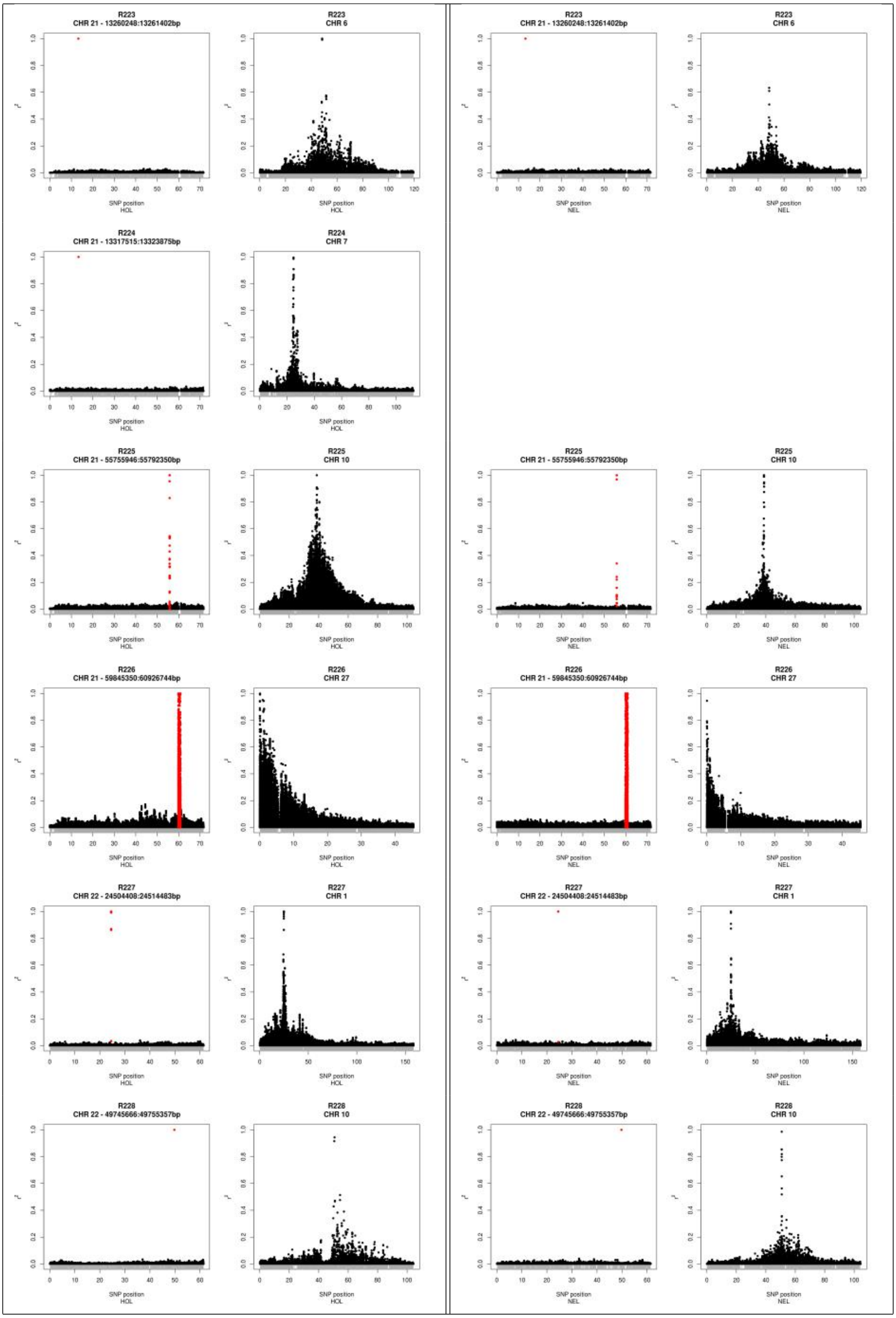
R223
CHR 21 - 13260248:13261402bp
SNP position
HOL
R223
CHR 6
SNP position
HOL
R223
CHR 21 - 13260248:13261402bp
SNP position
NEL
R223
CHR 6
SNP position
NEL
R224
CHR 21 - 13317515:13323875bp
SNP position
HOL
R224
CHR 7
SNP position
HOL
R225
CHR 21 - 55755946:55792350bp
SNP position
HOL
R225
CHR 10
SNP position
HOL
R225
CHR 21 - 55755946:55792350bp
SNP position
NEL
R225
CHR 10
SNP position
NEL
R226
CHR 21 - 59845350:60926744bp
SNP position
HOL
R226
CHR 27
SNP position
HOL
R226
CHR 21 - 59845350:60926744bp
SNP position
NEL
R226
CHR 27
SNP position
NEL
R227
CHR 22 - 24504408:24514483bp
SNP position
HOL
R227
CHR 1
SNP position
HOL
R227
CHR 22 - 24504408:24514483bp
SNP position
NEL
R227
CHR 1
SNP position
NEL
R228
CHR 22 - 49745666:49755357bp
SNP position
HOL
R228
CHR 10
SNP position
HOL
R228
CHR 22 - 49745666:49755357bp
SNP position
NEL
R228
CHR 10
SNP position
NEL

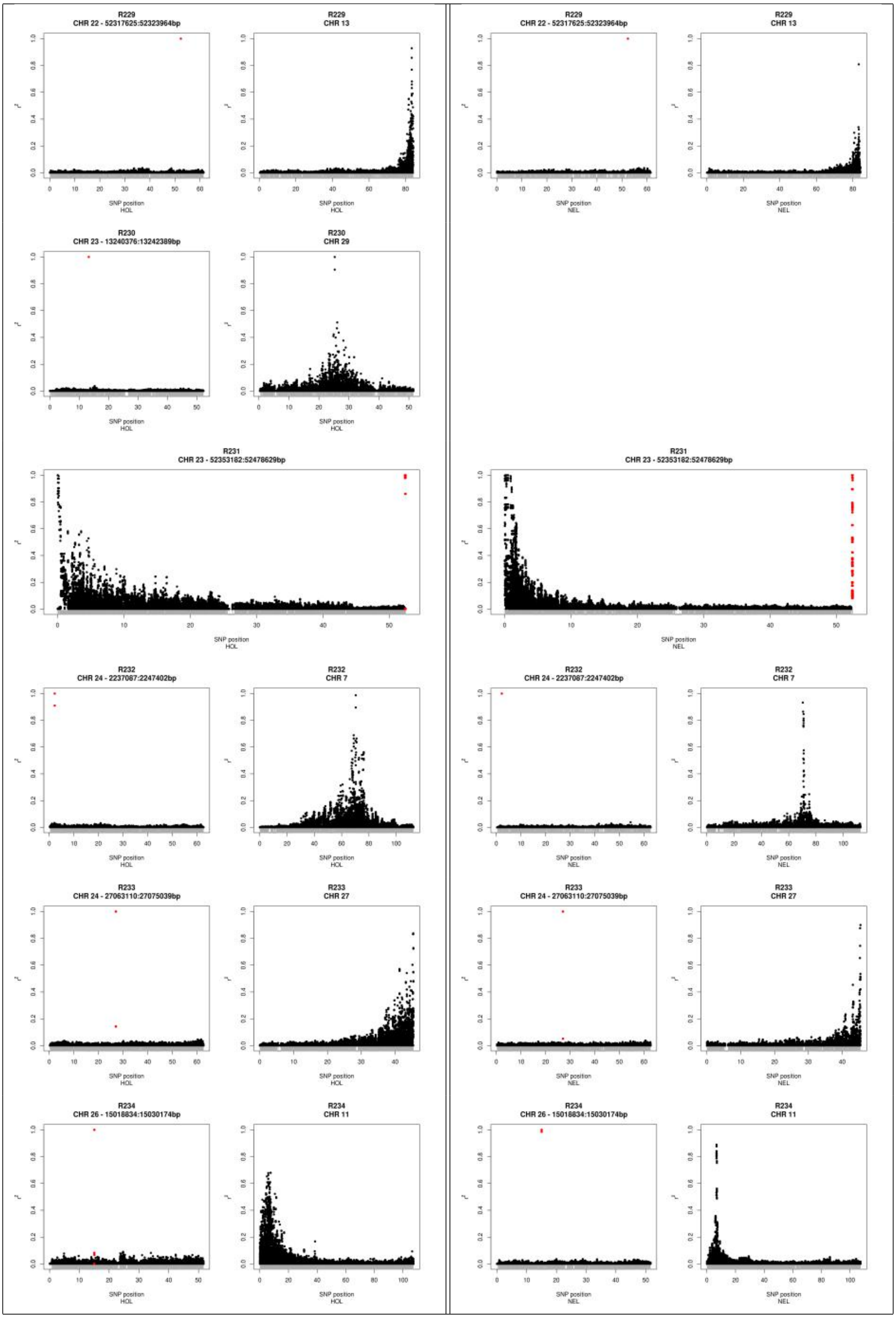
R229
CHR 22 - 52317625:52323964bp
SNP position
HOL
R229
CHR 13
SNP position
HOL
R229
CHR 22 - 52317625:52323964bp
SNP position
NEL
R229
CHR 13
SNP position
NEL
R230
CHR 23 - 13240376:13242389bp
SNP position
HOL
R230
CHR 29
SNP position
HOL
R231
CHR 23 - 52353182:52478629bp
SNP position
HOL
R231
CHR 23 - 52353182:52478629bp
SNP position
NEL
R232
CHR 24 - 2237087:2247402bp
SNP position
HOL
R232
CHR 7
SNP position
HOL
R232
CHR 24 - 2237087:2247402bp
SNP position
NEL
R232
CHR 7
SNP position
NEL
R233
CHR 24 - 27063110:27075039bp
SNP position
HOL
R233
CHR 27
SNP position
HOL
R233
CHR 24 - 27063110:27075039bp
SNP position
NEL
R233
CHR 27
SNP position
NEL
R234
CHR 26 - 15018834:15030174bp
SNP position
HOL
R234
CHR 11
SNP position
HOL
R234
CHR 26 - 15018834:15030174bp
SNP position
NEL
R234
CHR 11
SNP position
NEL

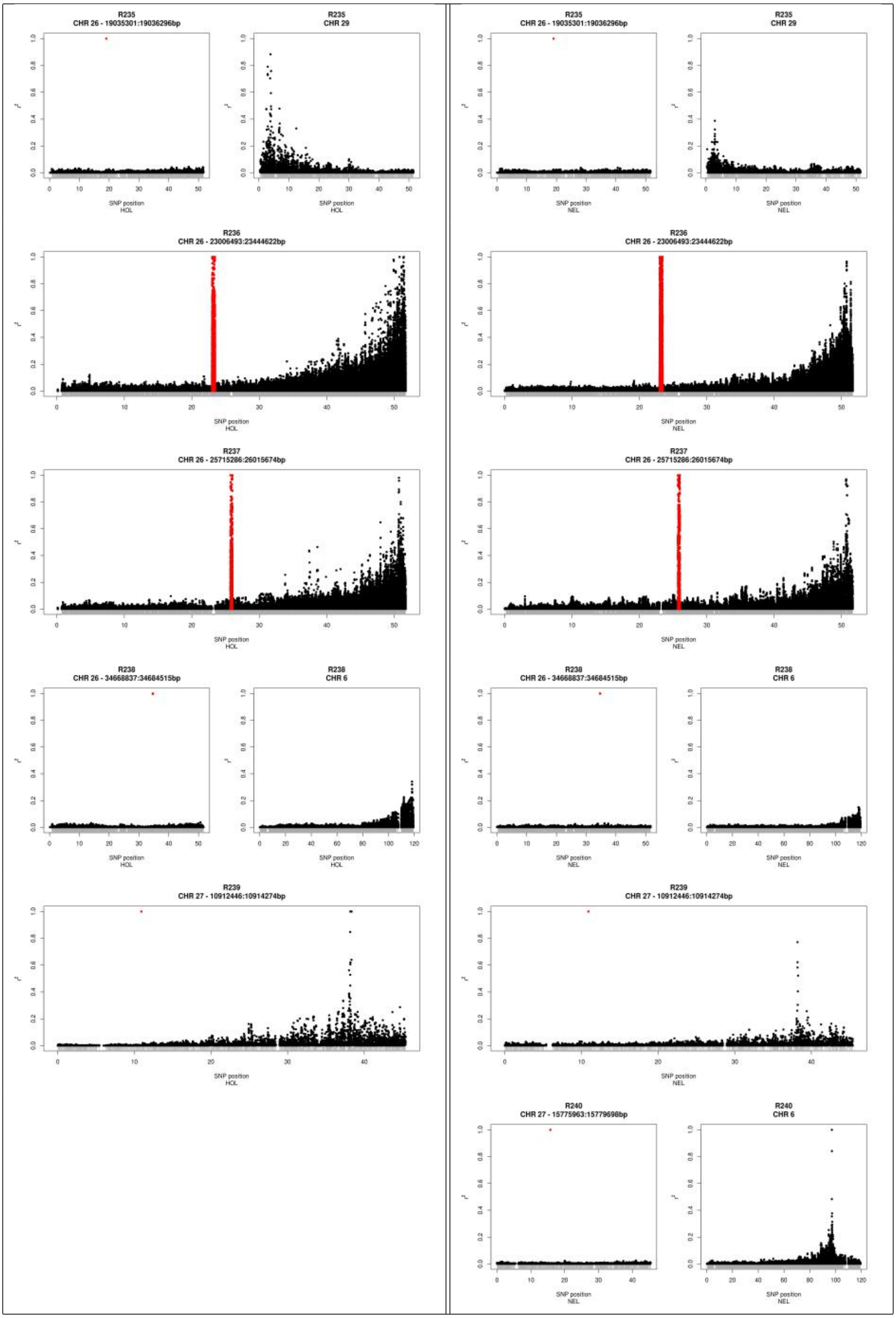
R235
CHR 26 - 19035301:19036296bp
SNP position
HOL
R235
CHR 29
SNP position
HOL
R236
CHR 26 - 23006493:23444622bp
SNP position
HOL
R237
CHR 26 - 25715286:26015674bp
SNP position
HOL
R238
CHR 26 - 34668837:34684515bp
SNP position
HOL
R238
CHR 6
SNP position
HOL
R239
CHR 27 - 10912446:10914274bp
SNP position
HOL
R235
CHR 26 - 19035301:19036296bp
SNP position
NEL
R235
CHR 29
SNP position
NEL
R236
CHR 26 - 23006493:23444622bp
SNP position
NEL
R237
CHR 26 - 25715286:26015674bp
SNP position
NEL
R238
CHR 26 - 34668837:34684515bp
SNP position
NEL
R238
CHR 6
SNP position
NEL
R239
CHR 27 - 10912446:10914274bp
SNP position
NEL
R240
CHR 27 - 15775963:15779698bp
SNP position
NEL
R240
CHR 6
SNP position
NEL

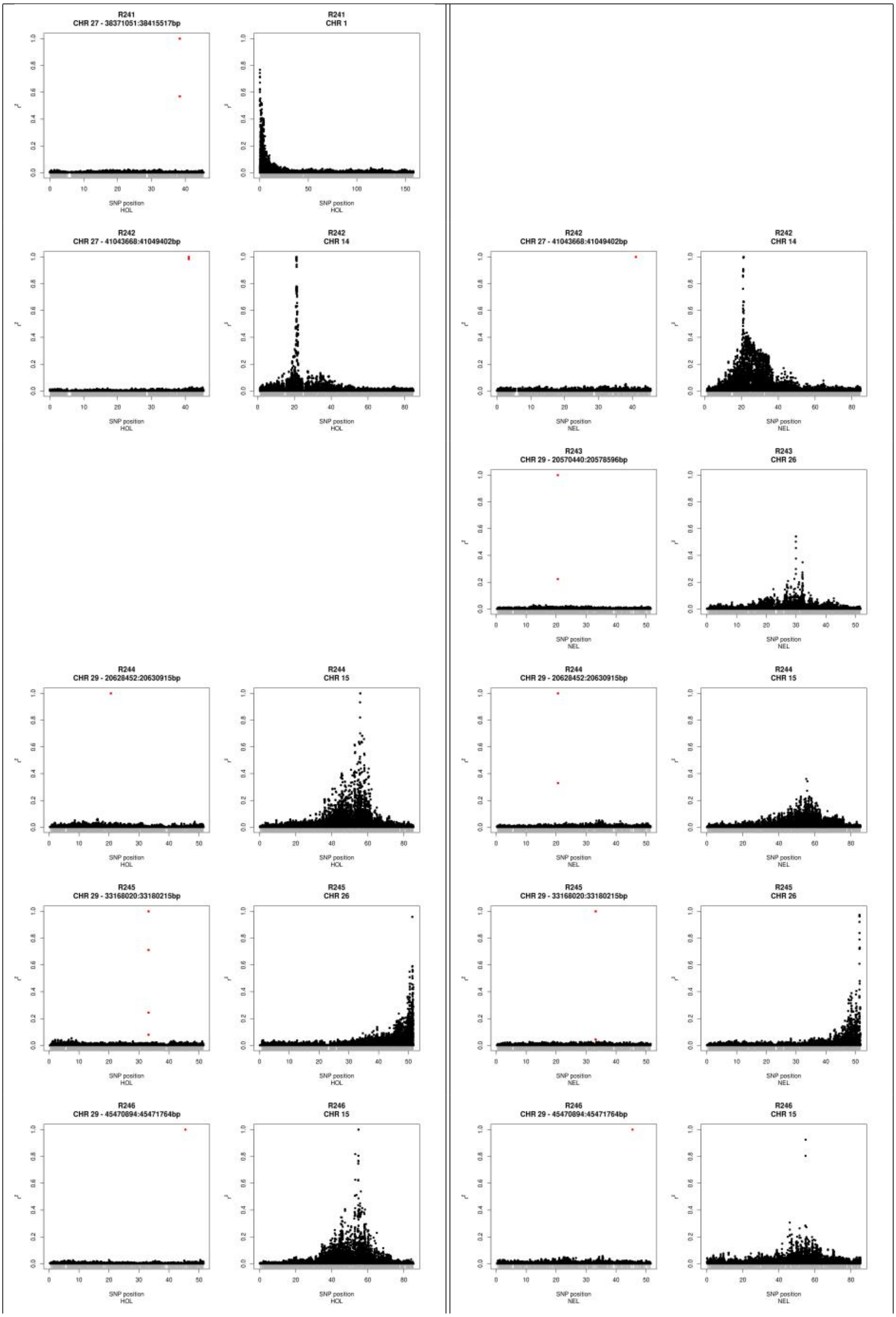
R241
CHR 27 - 38371051:38415517bp
R241
CHR 1
R242
CHR 27 - 41043668:41049402bp
R242
CHR 14
R242
CHR 27 - 41043668:41049402bp
R242
CHR 14
R243
CHR 29 - 20570440:20578596bp
R243
CHR 26
R244
CHR 29 - 20628452:20630915bp
R244
CHR 15
R244
CHR 29 - 20628452:20630915bp
R244
CHR 15
R245
CHR 29 - 33168020:33180215bp
R245
CHR 26
R245
CHR 29 - 33168020:33180215bp
R245
CHR 26
R246
CHR 29 - 45470894:45471764bp
R246
CHR 15
R246
CHR 29 - 45470894:45471764bp
R246
CHR 15
r²
SNP position
HOL
NEL
