# Supplementary material for: Revealing misassembled segments in the bovine reference genome by high resolution linkage disequilibrium scan
Source: BMC Genomics. 2016 Sep 5;17(1):705. doi: 10.1186/s12864-016-3049-8 (PMC5011828; doi:10.1186/s12864-016-3049-8)
Supplement: Additional file 7: Figure S6. — Partially Misassembled Contig (PmisCon). It presents all cases of partially misassembled contigs of the reference genome found in this study (PDF 7832 kb) [file 12864_2016_3049_MOESM7_ESM.pdf]

Figure S6 Partially Misassembled Contig (PMisCon). Left and right columns represent misplacements (red dots) found in Holstein and Nellore, respectively.

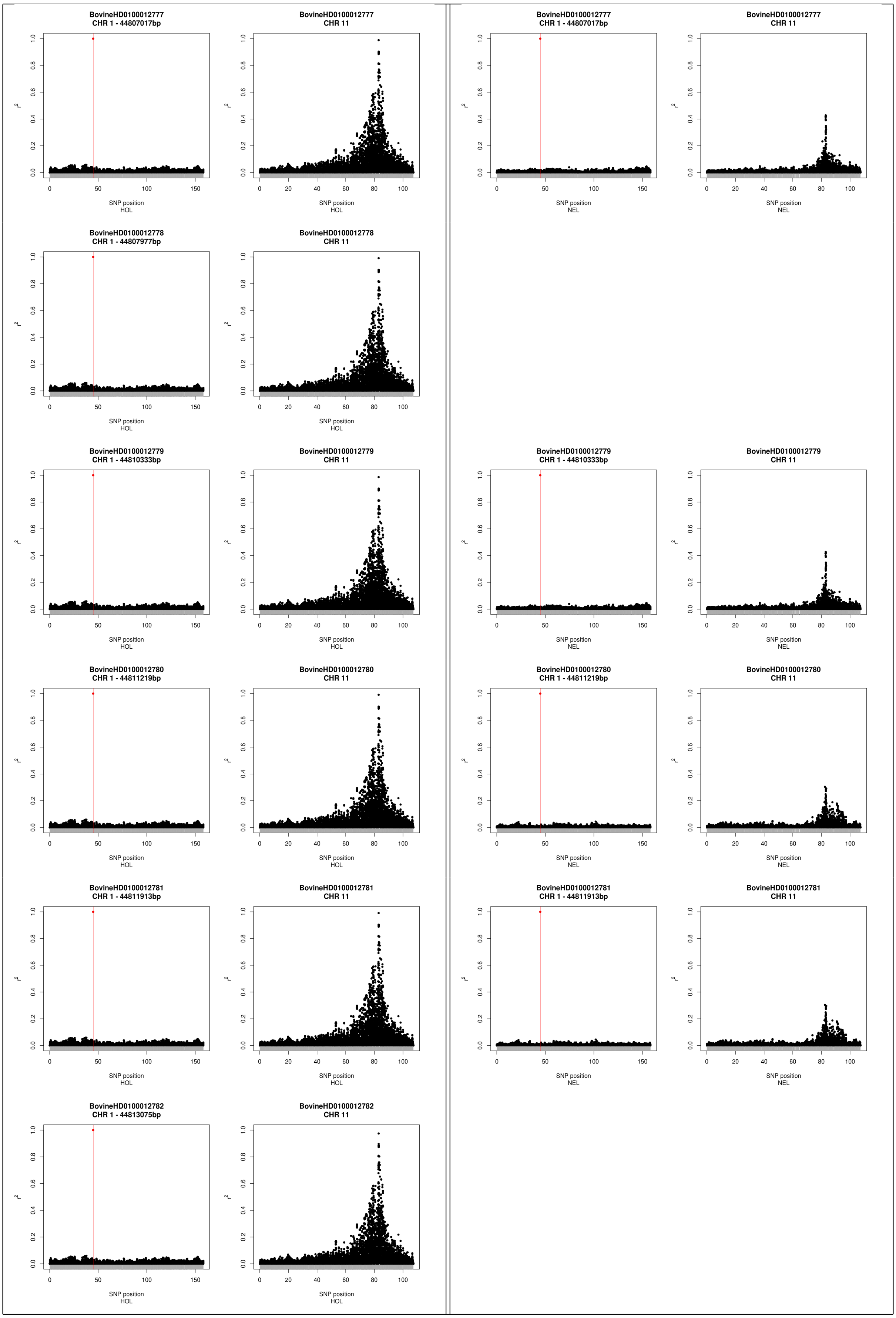

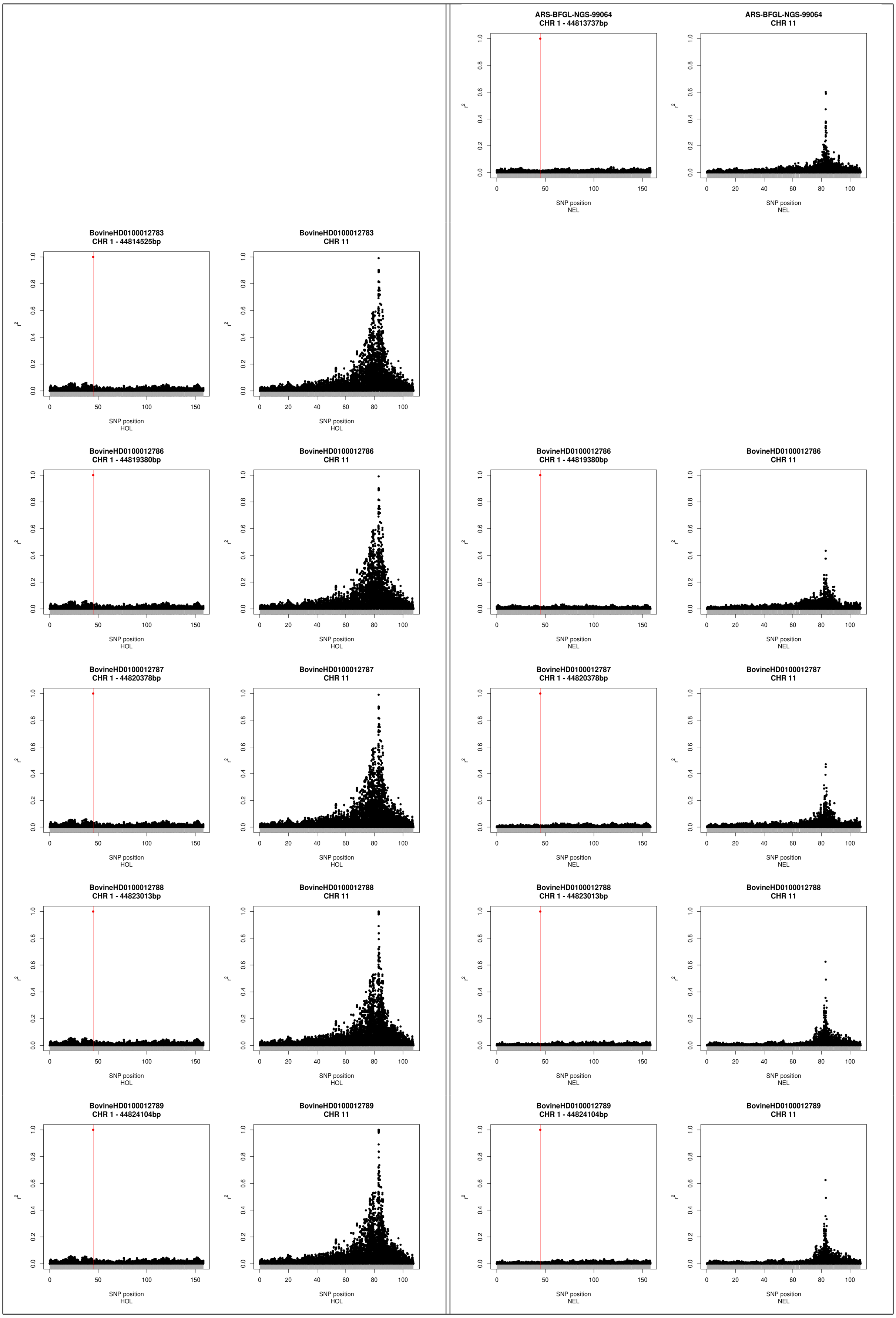
ARS-BFGL-NGS-99064
CHR 1 - 44813737bp
ARS-BFGL-NGS-99064
CHR 11
BovineHD0100012783
CHR 1 - 44814525bp
BovineHD0100012783
CHR 11
BovineHD0100012786
CHR 1 - 44819380bp
BovineHD0100012786
CHR 11
BovineHD0100012787
CHR 1 - 44820378bp
BovineHD0100012787
CHR 11
BovineHD0100012788
CHR 1 - 44823013bp
BovineHD0100012788
CHR 11
BovineHD0100012789
CHR 1 - 44824104bp
BovineHD0100012789
CHR 11
r²
SNP position
HOL
NEL

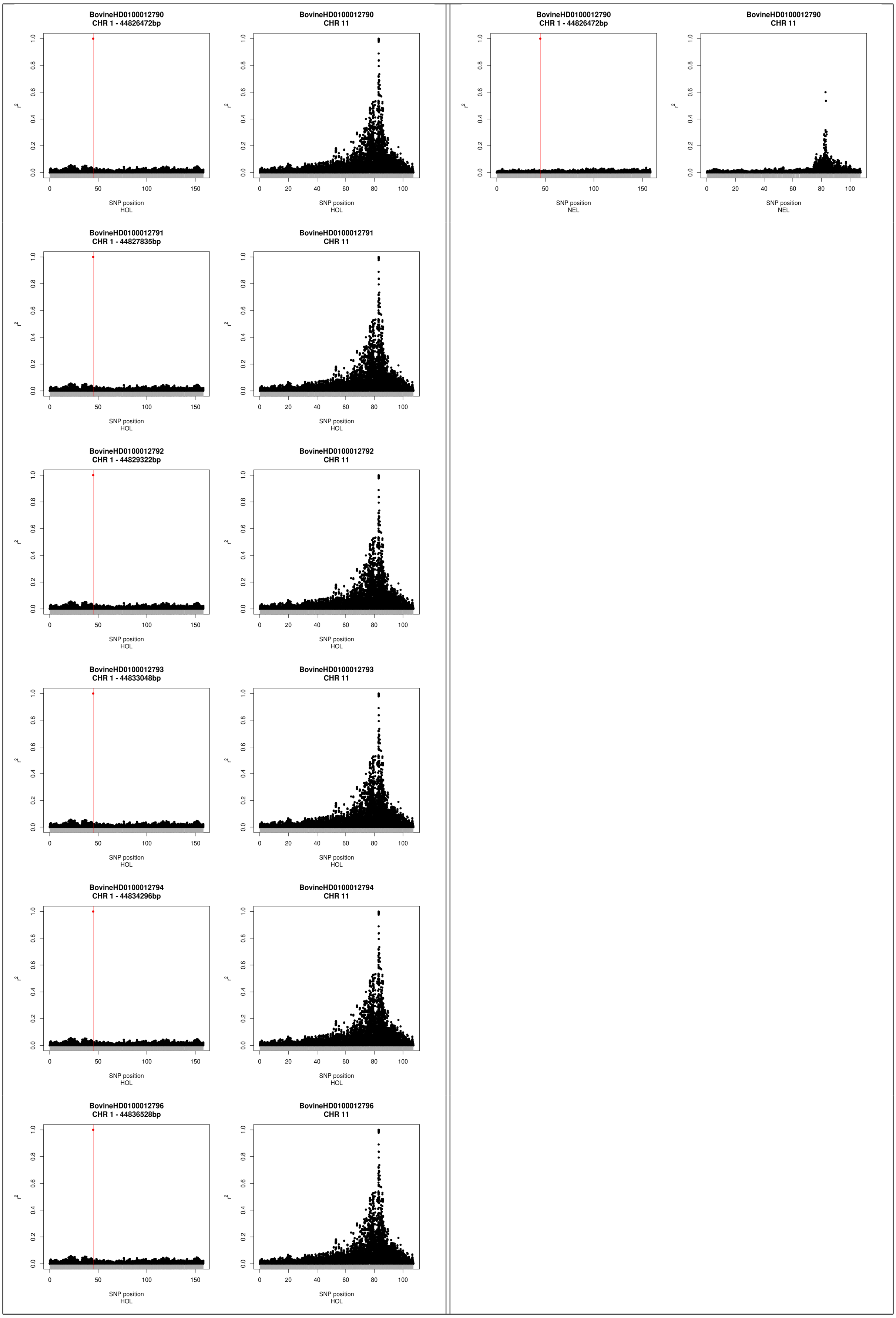
BovineHD0100012790
CHR 1 - 44826472bp
BovineHD0100012790
CHR 11
BovineHD0100012790
CHR 1 - 44826472bp
BovineHD0100012790
CHR 11
SNP position
HOL
SNP position
NEL
BovineHD0100012791
CHR 1 - 44827835bp
BovineHD0100012791
CHR 11
BovineHD0100012792
CHR 1 - 44829322bp
BovineHD0100012792
CHR 11
BovineHD0100012793
CHR 1 - 44833048bp
BovineHD0100012793
CHR 11
BovineHD0100012794
CHR 1 - 44834296bp
BovineHD0100012794
CHR 11
BovineHD0100012796
CHR 1 - 44836528bp
BovineHD0100012796
CHR 11
r^2

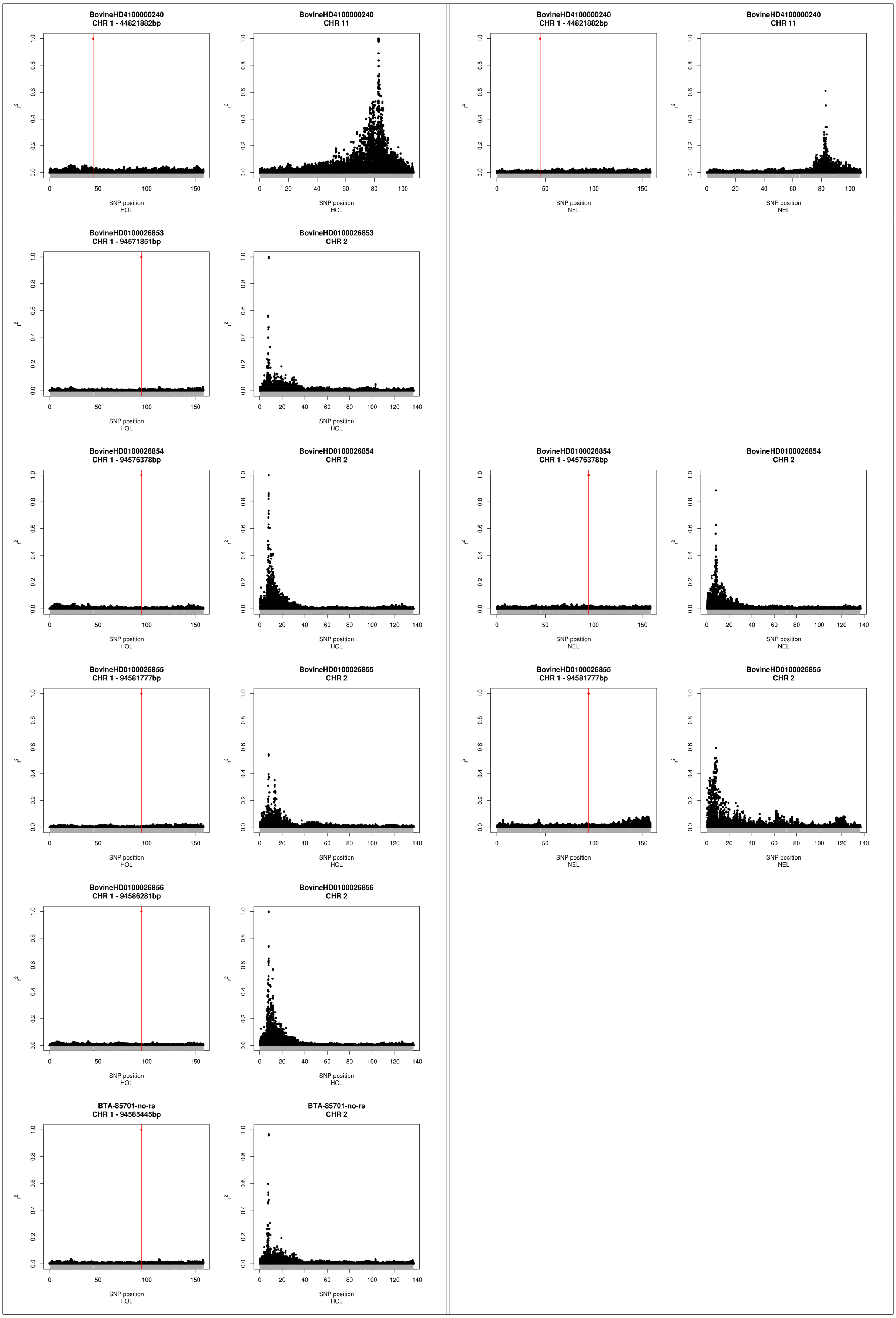
BovineHD4100000240
CHR 1 - 44821882bp
BovineHD4100000240
CHR 11
BovineHD4100000240
CHR 1 - 44821882bp
BovineHD4100000240
CHR 11
BovineHD0100026853
CHR 1 - 94571851bp
BovineHD0100026853
CHR 2
BovineHD0100026854
CHR 1 - 94576378bp
BovineHD0100026854
CHR 2
BovineHD0100026854
CHR 1 - 94576378bp
BovineHD0100026854
CHR 2
BovineHD0100026855
CHR 1 - 94581777bp
BovineHD0100026855
CHR 2
BovineHD0100026855
CHR 1 - 94581777bp
BovineHD0100026855
CHR 2
BovineHD0100026856
CHR 1 - 94586281bp
BovineHD0100026856
CHR 2
BTA-85701-no-rs
CHR 1 - 94585445bp
BTA-85701-no-rs
CHR 2
r^2
SNP position
HOL
NEL

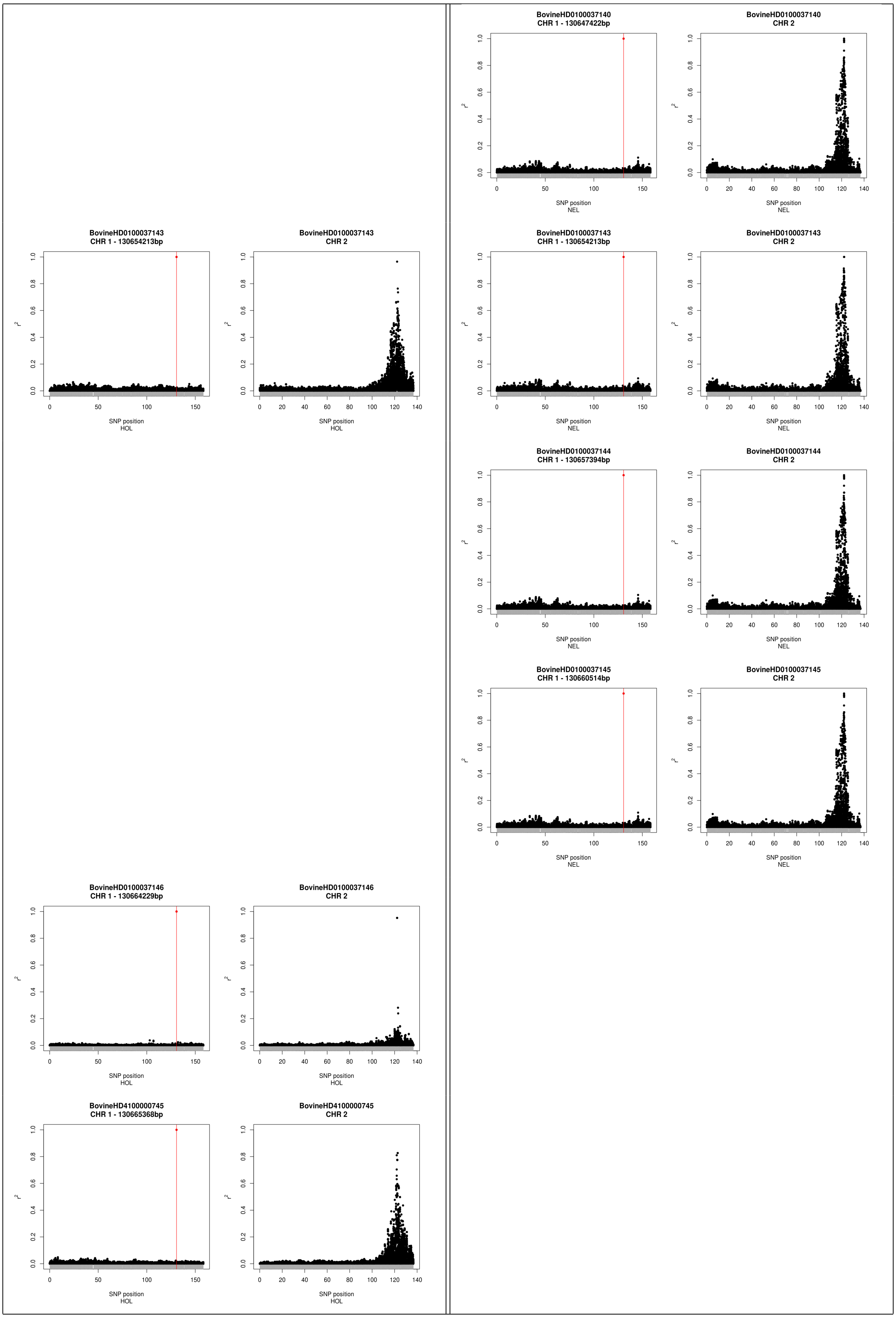
BovineHD0100037140
CHR 1 - 130647422bp
BovineHD0100037140
CHR 2
BovineHD0100037143
CHR 1 - 130654213bp
BovineHD0100037143
CHR 2
BovineHD0100037143
CHR 1 - 130654213bp
BovineHD0100037143
CHR 2
BovineHD0100037144
CHR 1 - 130657394bp
BovineHD0100037144
CHR 2
BovineHD0100037145
CHR 1 - 130660514bp
BovineHD0100037145
CHR 2
BovineHD0100037146
CHR 1 - 130664229bp
BovineHD0100037146
CHR 2
BovineHD4100000745
CHR 1 - 130665368bp
BovineHD4100000745
CHR 2
r^2
SNP position
HOL
NEL

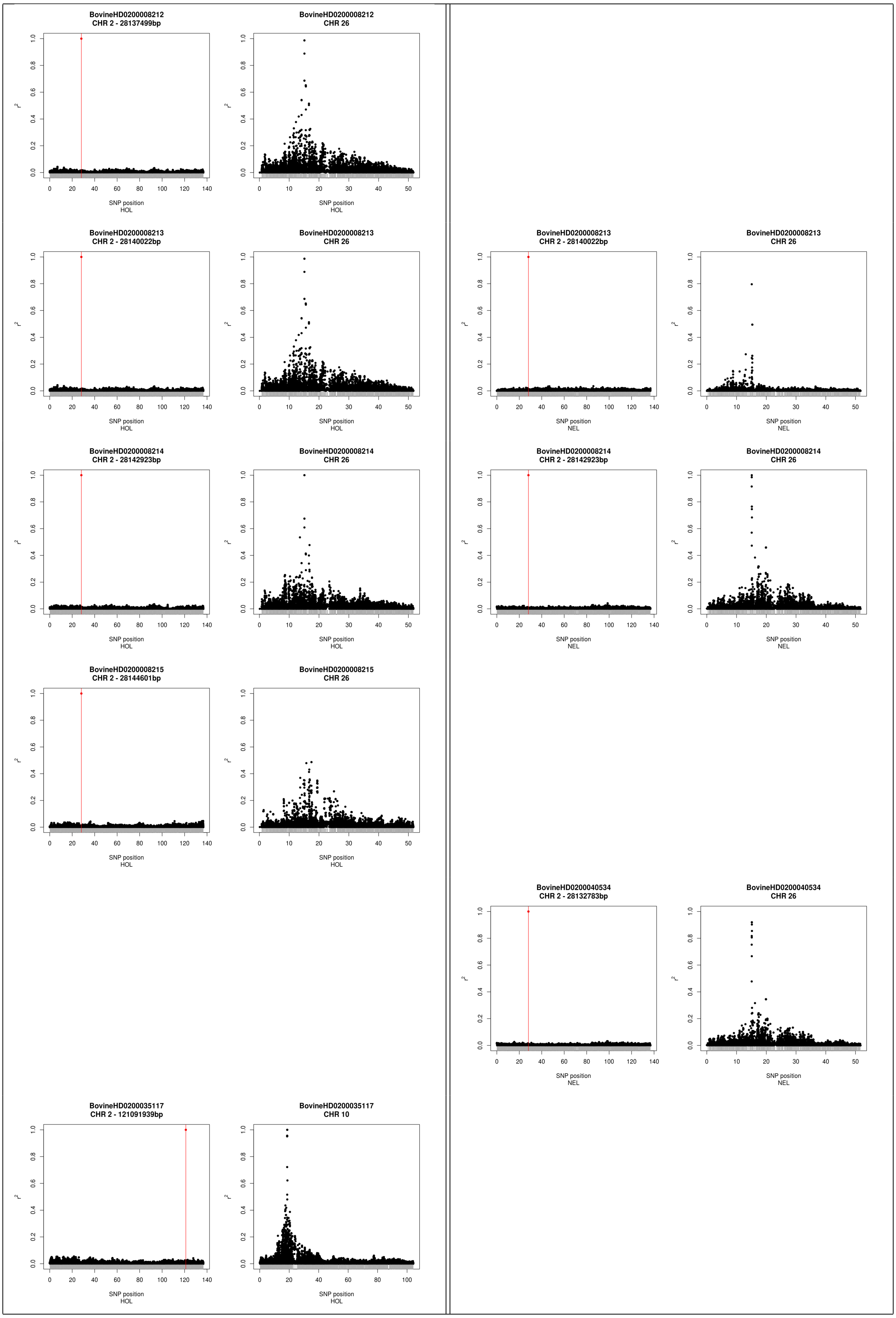
BovineHD0200008212
CHR 2 - 28137499bp
BovineHD0200008212
CHR 26
BovineHD0200008213
CHR 2 - 28140022bp
BovineHD0200008213
CHR 26
BovineHD0200008213
CHR 2 - 28140022bp
BovineHD0200008213
CHR 26
BovineHD0200008214
CHR 2 - 28142923bp
BovineHD0200008214
CHR 26
BovineHD0200008214
CHR 2 - 28142923bp
BovineHD0200008214
CHR 26
BovineHD0200008215
CHR 2 - 28144601bp
BovineHD0200008215
CHR 26
BovineHD0200040534
CHR 2 - 28132783bp
BovineHD0200040534
CHR 26
BovineHD0200035117
CHR 2 - 121091939bp
BovineHD0200035117
CHR 10
r²
SNP position
HOL
NEL

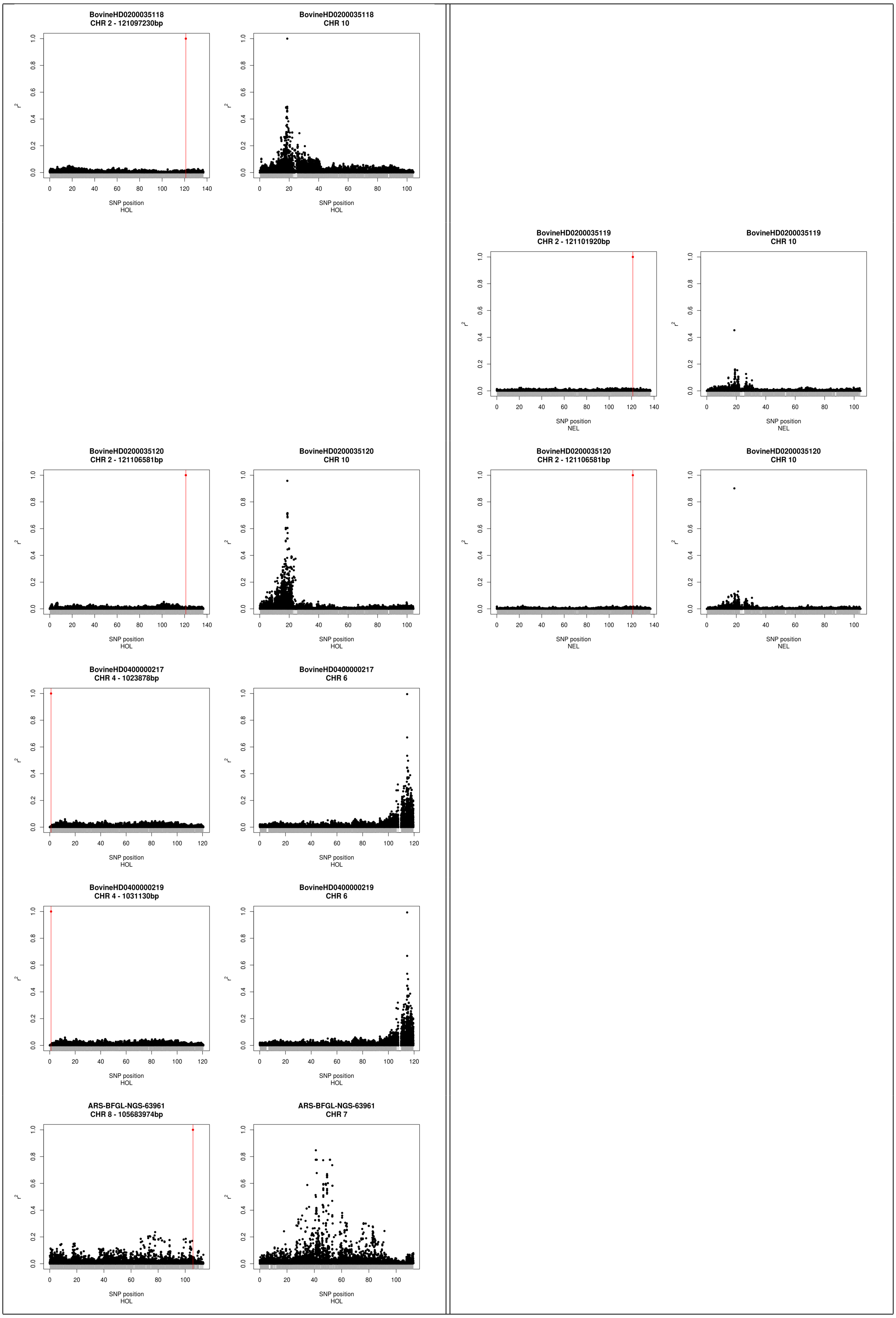
BovineHD0200035118
CHR 2 - 121097230bp
BovineHD0200035118
CHR 10
BovineHD0200035119
CHR 2 - 121101920bp
BovineHD0200035119
CHR 10
BovineHD0200035120
CHR 2 - 121106581bp
BovineHD0200035120
CHR 10
BovineHD0200035120
CHR 2 - 121106581bp
BovineHD0200035120
CHR 10
BovineHD0400000217
CHR 4 - 1023878bp
BovineHD0400000217
CHR 6
BovineHD0400000219
CHR 4 - 1031130bp
BovineHD0400000219
CHR 6
ARS-BFGL-NGS-63961
CHR 8 - 105683974bp
ARS-BFGL-NGS-63961
CHR 7
r²
SNP position
HOL
NEL

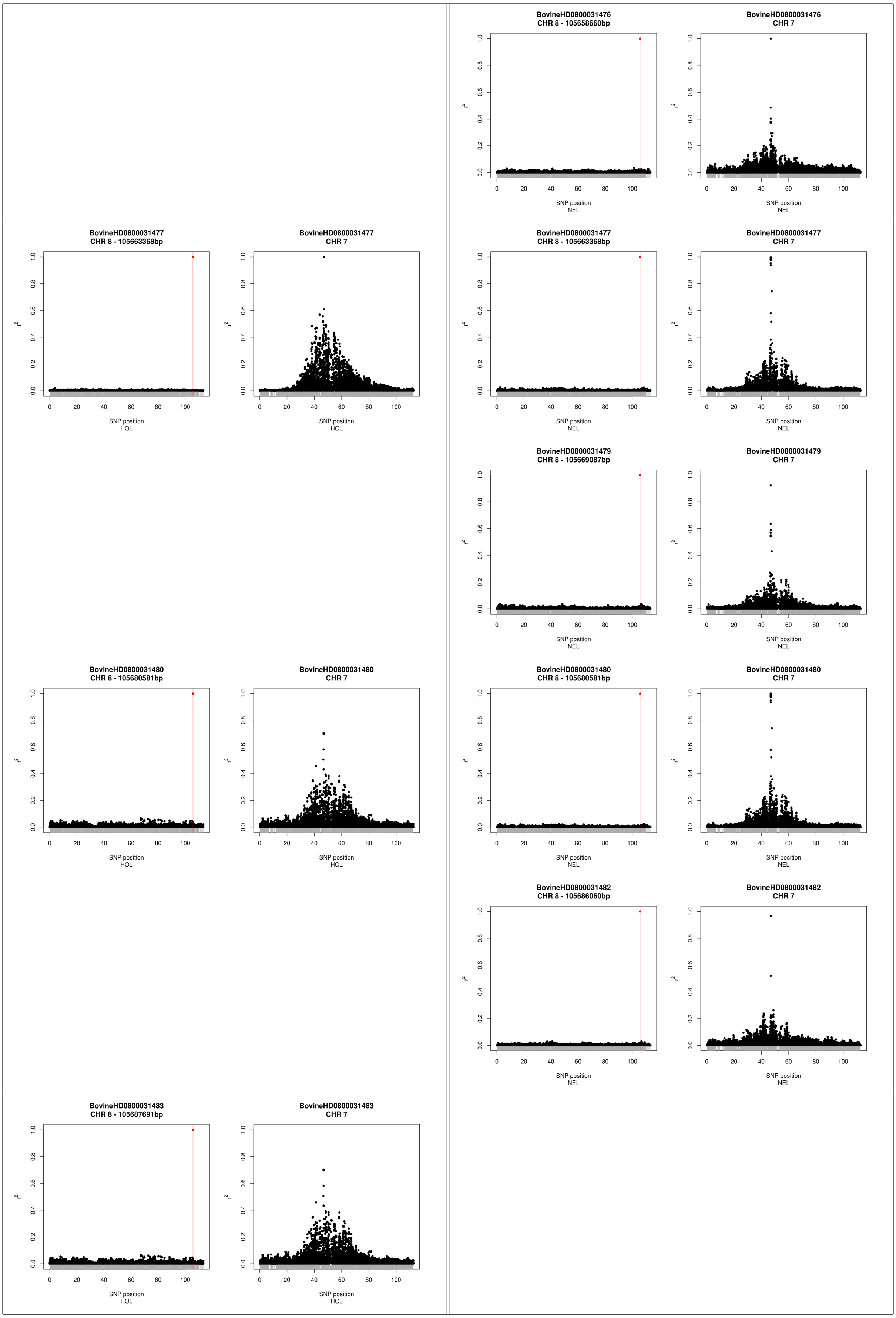
BovineHD0800031476
CHR 8 - 105658660bp
BovineHD0800031476
CHR 7
BovineHD0800031477
CHR 8 - 105663368bp
BovineHD0800031477
CHR 7
BovineHD0800031479
CHR 8 - 105669087bp
BovineHD0800031479
CHR 7
BovineHD0800031480
CHR 8 - 105680581bp
BovineHD0800031480
CHR 7
BovineHD0800031482
CHR 8 - 105686060bp
BovineHD0800031482
CHR 7
BovineHD0800031483
CHR 8 - 105687691bp
BovineHD0800031483
CHR 7
r^2
SNP position
HOL
NEL

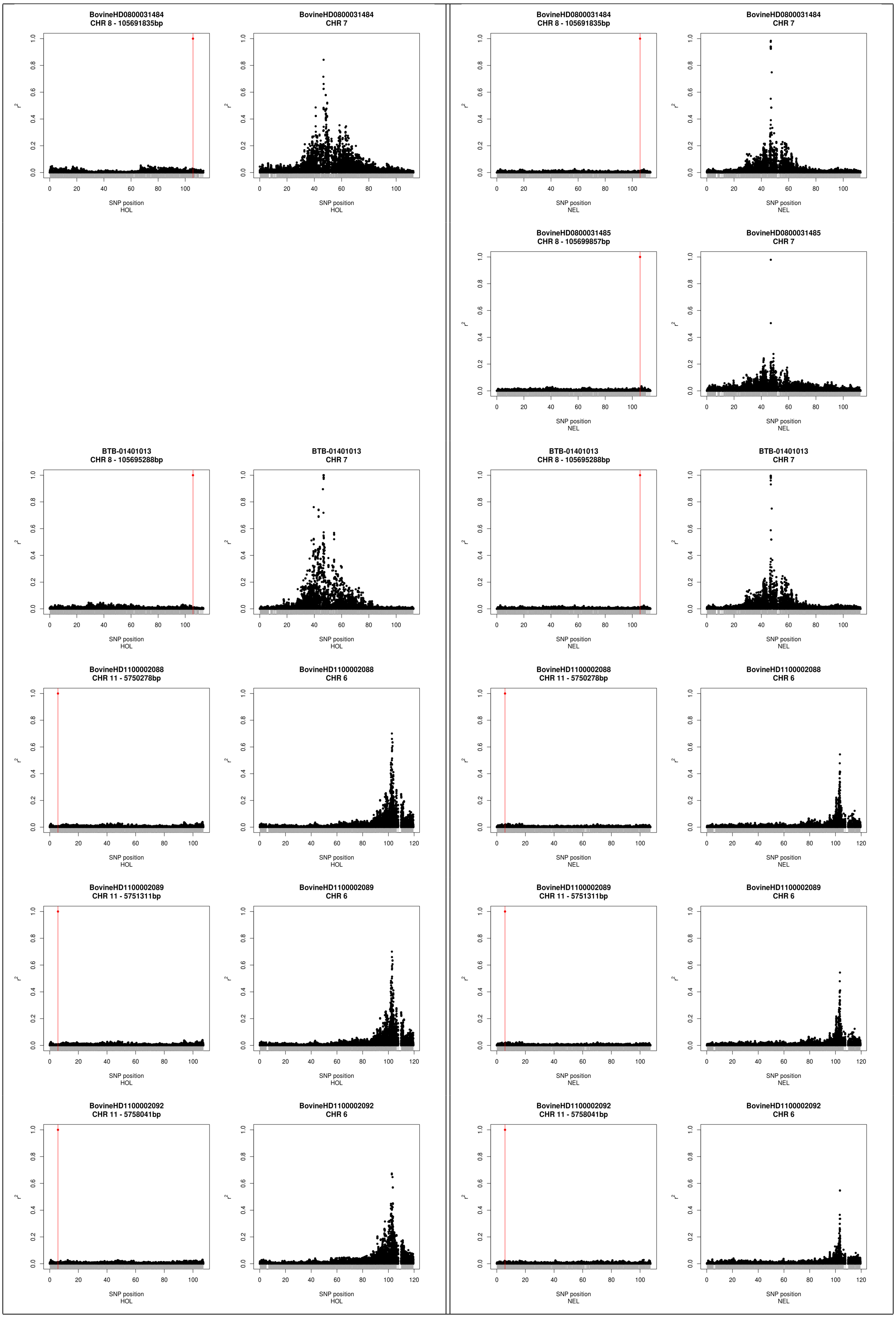
BovineHD0800031484
CHR 8 - 105691835bp
BovineHD0800031484
CHR 7
SNP position
HOL
BTB-01401013
CHR 8 - 105695288bp
BTB-01401013
CHR 7
BovineHD1100002088
CHR 11 - 5750278bp
BovineHD1100002088
CHR 6
BovineHD1100002089
CHR 11 - 5751311bp
BovineHD1100002089
CHR 6
BovineHD1100002092
CHR 11 - 5758041bp
BovineHD1100002092
CHR 6
BovineHD0800031485
CHR 8 - 105699857bp
BovineHD0800031485
CHR 7
NEL
r²

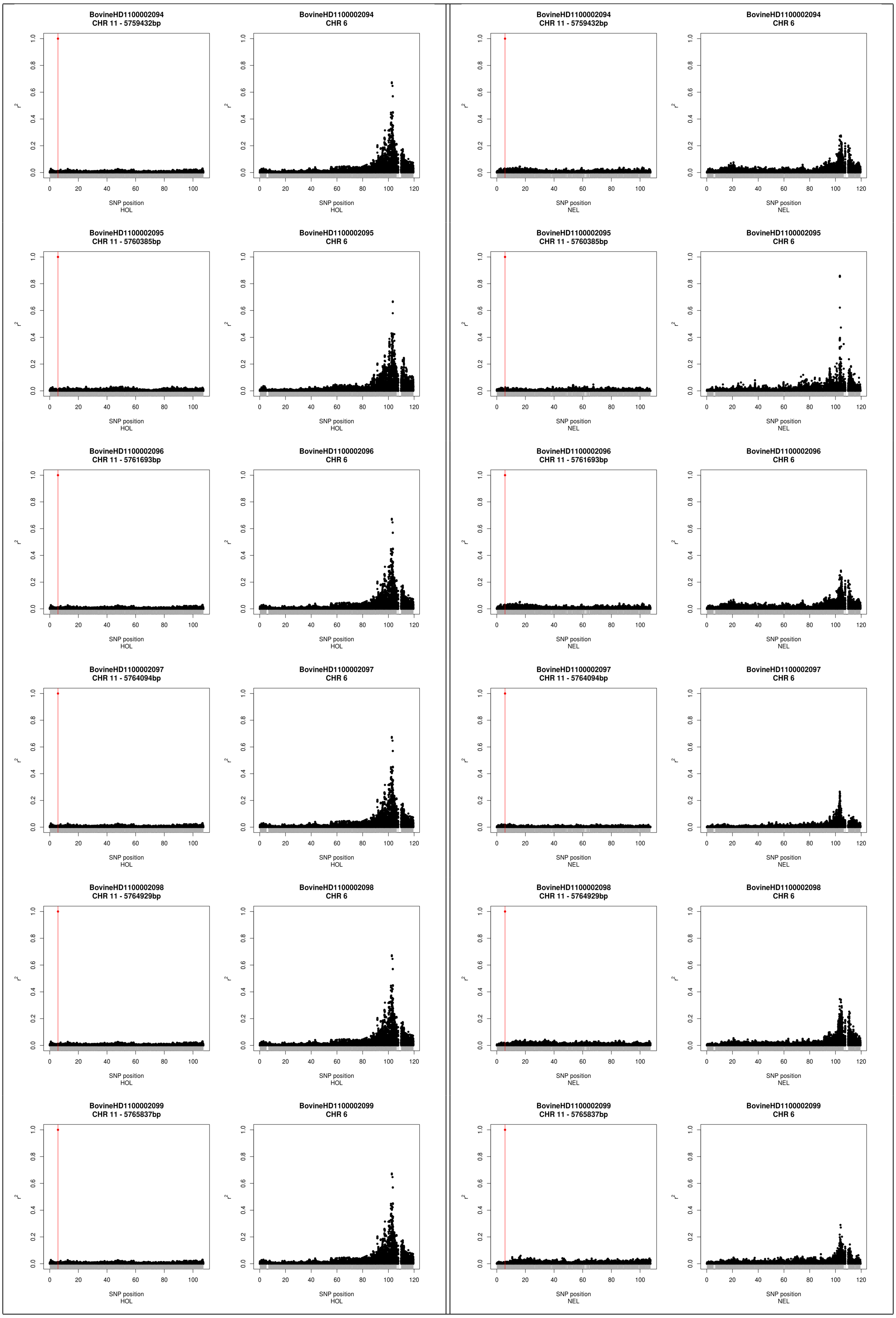
BovineHD1100002094
CHR 11 - 5759432bp
SNP position
HOL
BovineHD1100002094
CHR 6
SNP position
HOL
BovineHD1100002094
CHR 11 - 5759432bp
SNP position
NEL
BovineHD1100002094
CHR 6
SNP position
NEL
BovineHD1100002095
CHR 11 - 5760385bp
SNP position
HOL
BovineHD1100002095
CHR 6
SNP position
HOL
BovineHD1100002095
CHR 11 - 5760385bp
SNP position
NEL
BovineHD1100002095
CHR 6
SNP position
NEL
BovineHD1100002096
CHR 11 - 5761693bp
SNP position
HOL
BovineHD1100002096
CHR 6
SNP position
HOL
BovineHD1100002096
CHR 11 - 5761693bp
SNP position
NEL
BovineHD1100002096
CHR 6
SNP position
NEL
BovineHD1100002097
CHR 11 - 5764094bp
SNP position
HOL
BovineHD1100002097
CHR 6
SNP position
HOL
BovineHD1100002097
CHR 11 - 5764094bp
SNP position
NEL
BovineHD1100002097
CHR 6
SNP position
NEL
BovineHD1100002098
CHR 11 - 5764929bp
SNP position
HOL
BovineHD1100002098
CHR 6
SNP position
HOL
BovineHD1100002098
CHR 11 - 5764929bp
SNP position
NEL
BovineHD1100002098
CHR 6
SNP position
NEL
BovineHD1100002099
CHR 11 - 5765837bp
SNP position
HOL
BovineHD1100002099
CHR 6
SNP position
HOL
BovineHD1100002099
CHR 11 - 5765837bp
SNP position
NEL
BovineHD1100002099
CHR 6
SNP position
NEL

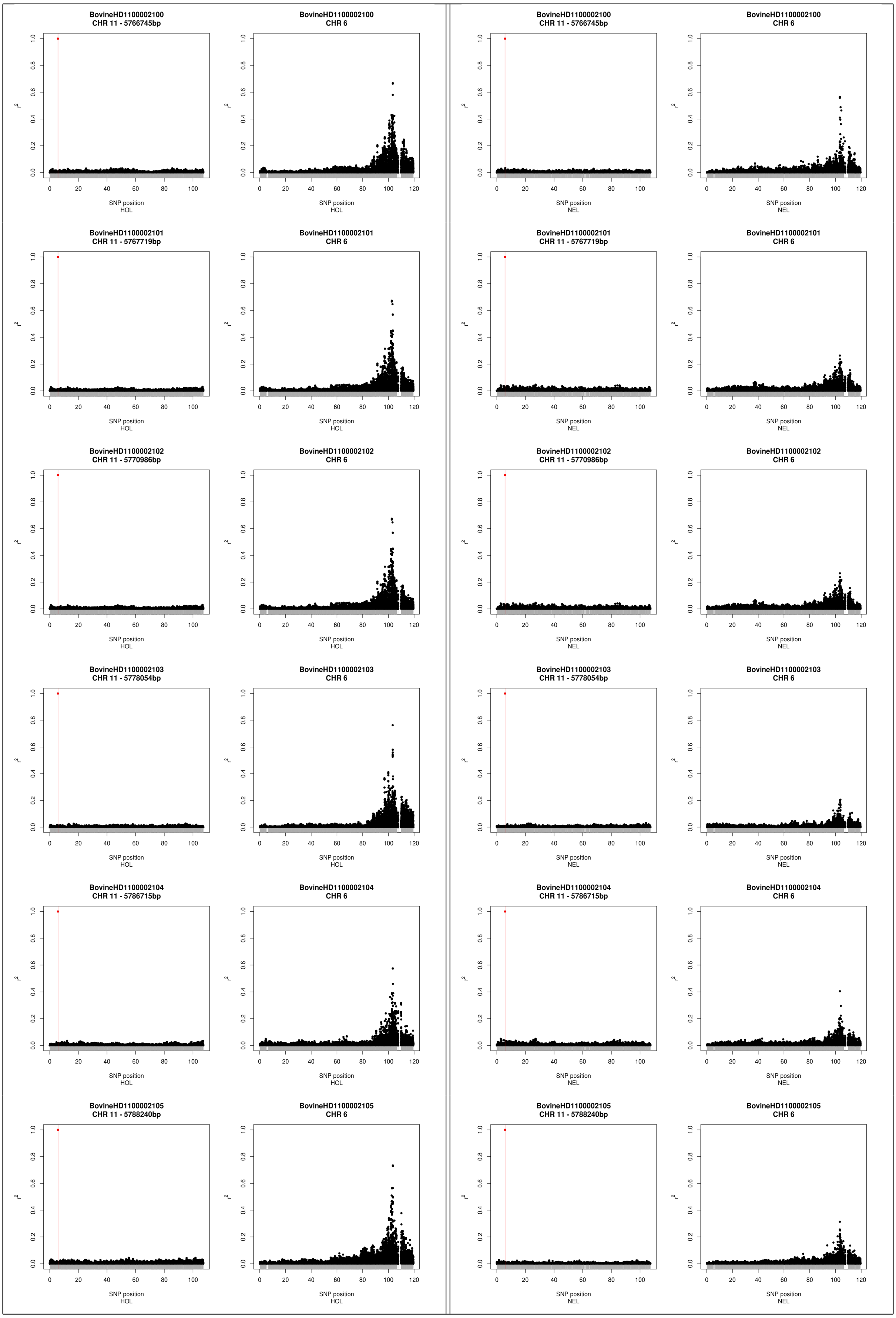
BovineHD1100002100
CHR 11 - 5766745bp
SNP position
HOL
BovineHD1100002100
CHR 6
SNP position
HOL
BovineHD1100002100
CHR 11 - 5766745bp
SNP position
NEL
BovineHD1100002100
CHR 6
SNP position
NEL
BovineHD1100002101
CHR 11 - 5767719bp
SNP position
HOL
BovineHD1100002101
CHR 6
SNP position
HOL
BovineHD1100002101
CHR 11 - 5767719bp
SNP position
NEL
BovineHD1100002101
CHR 6
SNP position
NEL
BovineHD1100002102
CHR 11 - 5770986bp
SNP position
HOL
BovineHD1100002102
CHR 6
SNP position
HOL
BovineHD1100002102
CHR 11 - 5770986bp
SNP position
NEL
BovineHD1100002102
CHR 6
SNP position
NEL
BovineHD1100002103
CHR 11 - 5778054bp
SNP position
HOL
BovineHD1100002103
CHR 6
SNP position
HOL
BovineHD1100002103
CHR 11 - 5778054bp
SNP position
NEL
BovineHD1100002103
CHR 6
SNP position
NEL
BovineHD1100002104
CHR 11 - 5786715bp
SNP position
HOL
BovineHD1100002104
CHR 6
SNP position
HOL
BovineHD1100002104
CHR 11 - 5786715bp
SNP position
NEL
BovineHD1100002104
CHR 6
SNP position
NEL
BovineHD1100002105
CHR 11 - 5788240bp
SNP position
HOL
BovineHD1100002105
CHR 6
SNP position
HOL
BovineHD1100002105
CHR 11 - 5788240bp
SNP position
NEL
BovineHD1100002105
CHR 6
SNP position
NEL

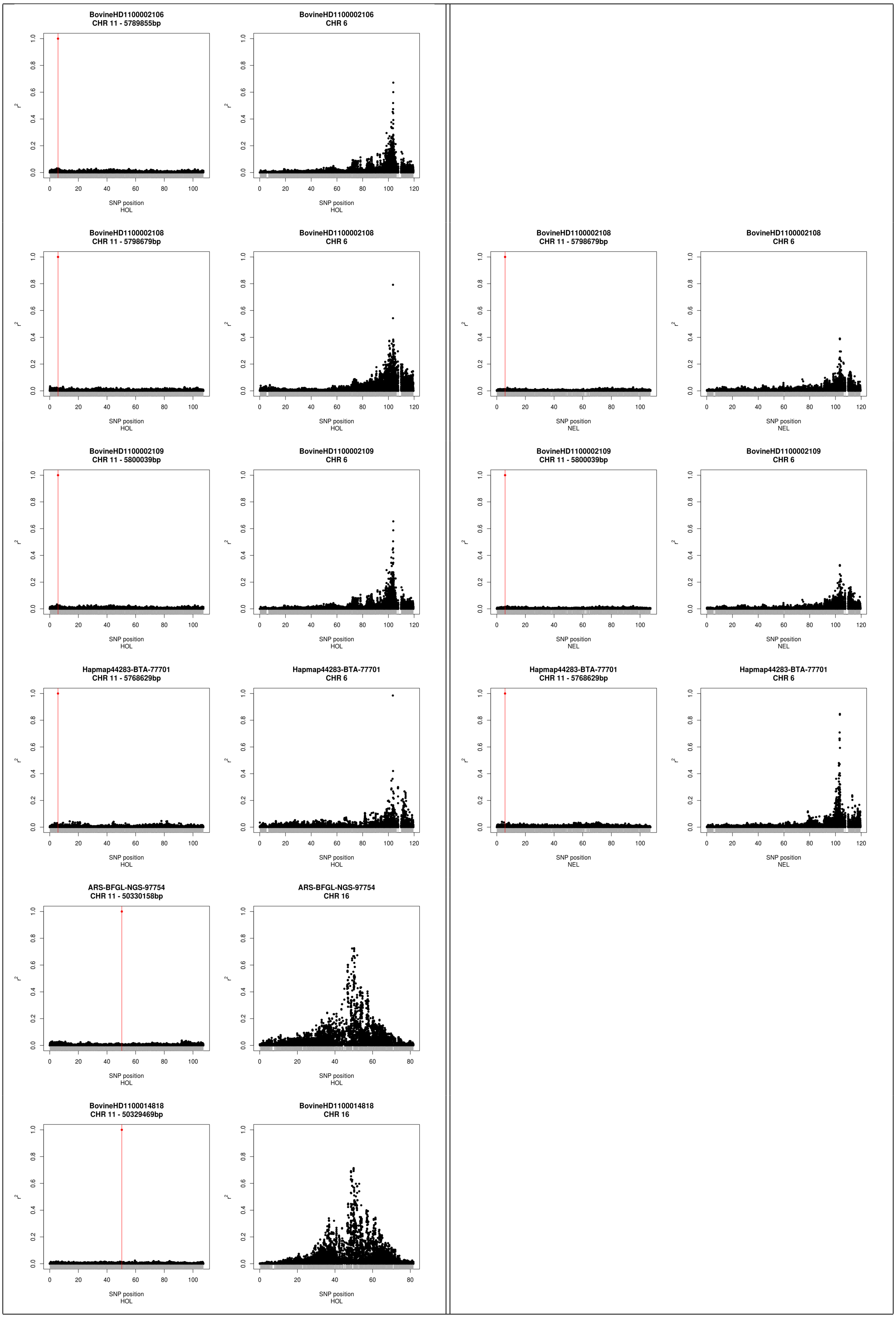
BovineHD1100002106
CHR 11 - 5789855bp
BovineHD1100002106
CHR 6
BovineHD1100002108
CHR 11 - 5798679bp
BovineHD1100002108
CHR 6
BovineHD1100002108
CHR 11 - 5798679bp
BovineHD1100002108
CHR 6
BovineHD1100002109
CHR 11 - 5800039bp
BovineHD1100002109
CHR 6
BovineHD1100002109
CHR 11 - 5800039bp
BovineHD1100002109
CHR 6
Hapmap44283-BTA-77701
CHR 11 - 5768629bp
Hapmap44283-BTA-77701
CHR 6
Hapmap44283-BTA-77701
CHR 11 - 5768629bp
Hapmap44283-BTA-77701
CHR 6
ARS-BFGL-NGS-97754
CHR 11 - 50330158bp
ARS-BFGL-NGS-97754
CHR 16
BovineHD1100014818
CHR 11 - 50329469bp
BovineHD1100014818
CHR 16
r^2
SNP position
HOL
NEL

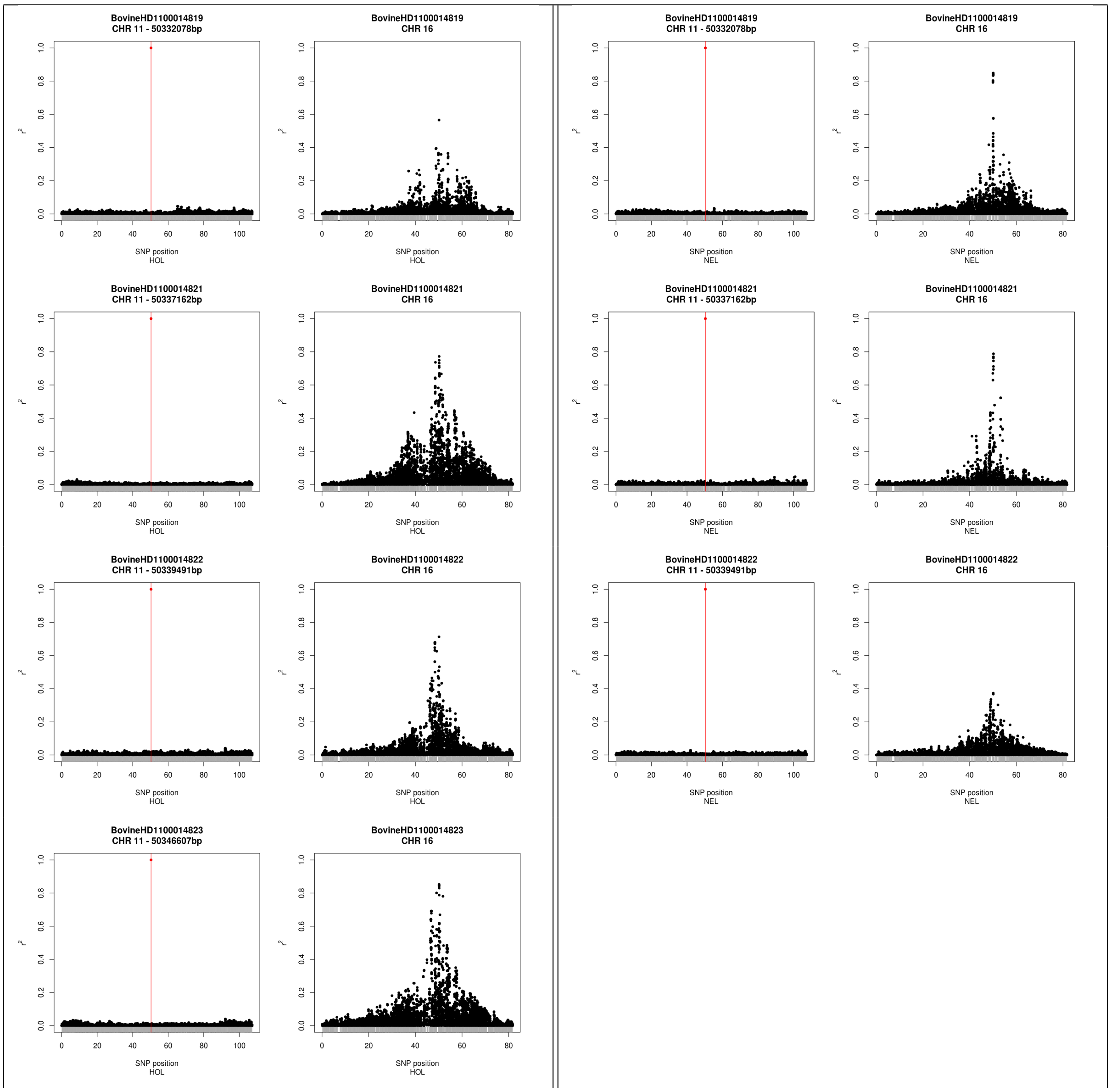
BovineHD1100014819
CHR 11 - 50332078bp
SNP position
HOL
BovineHD1100014819
CHR 16
SNP position
HOL
BovineHD1100014821
CHR 11 - 50337162bp
SNP position
HOL
BovineHD1100014821
CHR 16
SNP position
HOL
BovineHD1100014822
CHR 11 - 50339491bp
SNP position
HOL
BovineHD1100014822
CHR 16
SNP position
HOL
BovineHD1100014823
CHR 11 - 50346607bp
SNP position
HOL
BovineHD1100014823
CHR 16
SNP position
HOL
BovineHD1100014819
CHR 11 - 50332078bp
SNP position
NEL
BovineHD1100014819
CHR 16
SNP position
NEL
BovineHD1100014821
CHR 11 - 50337162bp
SNP position
NEL
BovineHD1100014821
CHR 16
SNP position
NEL
BovineHD1100014822
CHR 11 - 50339491bp
SNP position
NEL
BovineHD1100014822
CHR 16
SNP position
NEL
