# Supplementary material for: Revealing misassembled segments in the bovine reference genome by high resolution linkage disequilibrium scan
Source: BMC Genomics. 2016 Sep 5;17(1):705. doi: 10.1186/s12864-016-3049-8 (PMC5011828; doi:10.1186/s12864-016-3049-8)
Supplement: Additional file 8: — Tag marker count criteria. Describes the empirical criteria used for detecting candidate misplaced markers (PDF 229 kb) [file 12864_2016_3049_MOESM8_ESM.pdf]

Supplementary File S1. Tag marker count criteria

The criterion for selecting a Candidate Misplaced Marker (CMM) considering the largest tag marker count (>50%) was empirically defined, since high r^2 values are expected to be found between SNPs close to each other in the same chromosome.

Figure 1 shows the percentage of tag markers presenting high LD values (>0.5) with focal SNPs in different chromosomes in Holstein and Nellore datasets.

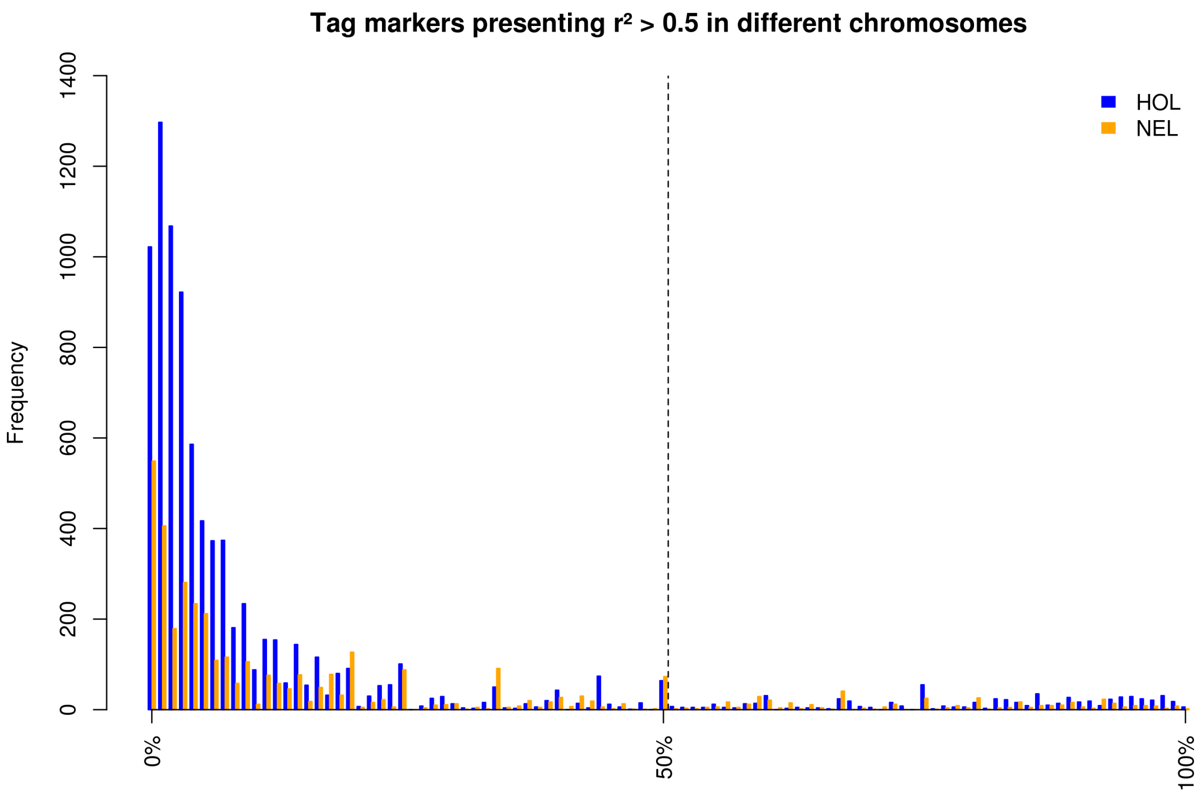

Figure 1. Histogram of the percentage of tag markers presenting r^2 values greater than 0.5 in different chromosomes. The vertical dashed line represents the 50% threshold of selecting a Candidate Misplaced marker (largest tag marker count).

As expected, the vast majority of SNPs show no high LD with SNPs in any other chromosome in the genome. It shows that our criteria for selecting cases where the number of tag markers in a different chromosome exceed the number of tag markers in the same chromosome satisfy the candidacy of a SNP to be misplaced.

Figure 2 shows the percentage of tag markers showing high LD values (>0.5) with focal SNPs far apart (>10 Mb) in the same chromosome.

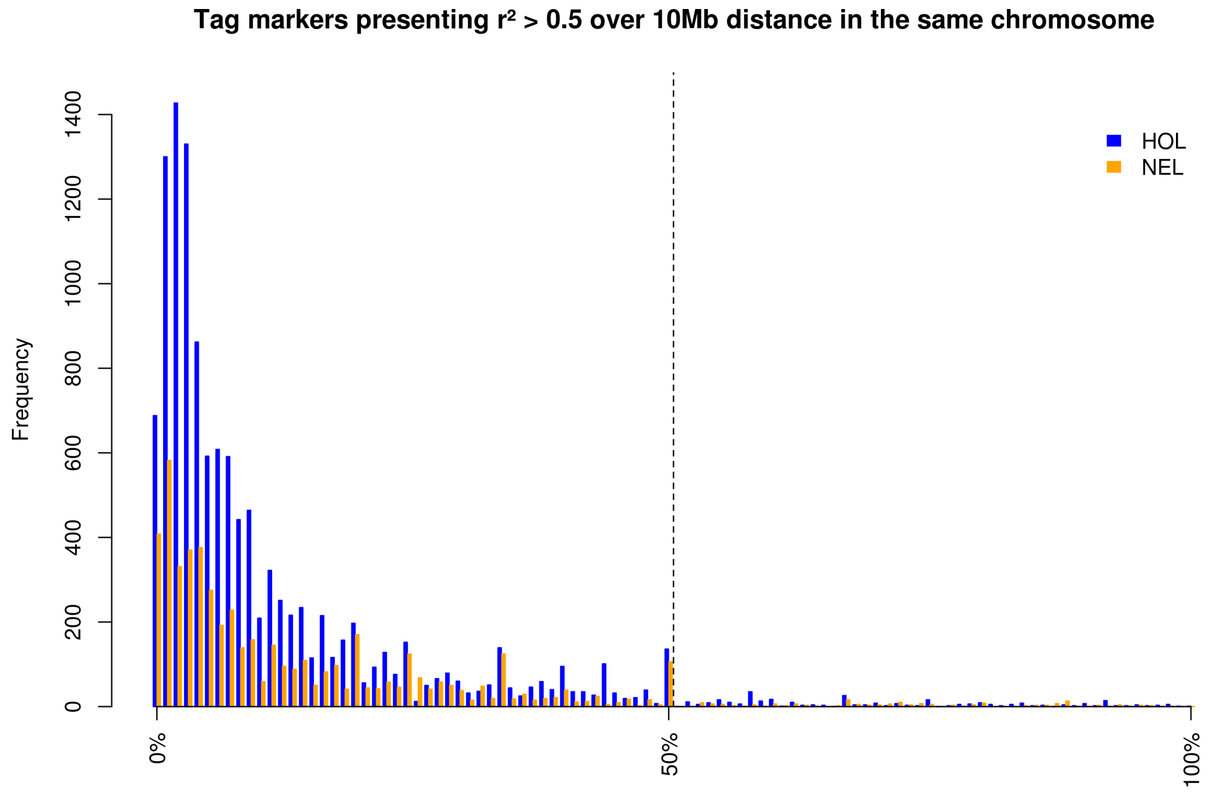

Figure 2. Histogram of the percentage of tag markers presenting r^2 values greater than 0.5 with the focal SNP far apart in the same chromosome. The vertical dashed line represents the 50% threshold of selecting a Candidate Misplaced Marker (largest tag marker count).

As before, the vast majority of SNPs do not present high r^2 values over 10 Mb distance, which reinforces the candidacy of a SNP to be misplaced whether it does not attend our criteria.
