# Supplementary material for: Revealing misassembled segments in the bovine reference genome by high resolution linkage disequilibrium scan
Source: BMC Genomics. 2016 Sep 5;17(1):705. doi: 10.1186/s12864-016-3049-8 (PMC5011828; doi:10.1186/s12864-016-3049-8)
Supplement: Additional file 9: — Benchmarking of the r2 calculations. Describes the computational performance for calculating all pair-wise r2 (PDF 301 kb) [file 12864_2016_3049_MOESM9_ESM.pdf]

Supplementary File S2. Benchmarking of the r² calculations

The benchmarking of the r² calculations was performed in a Dell Precision T7610 workstation with 16 GB RAM and 12 x 64-bit Intel Xeon 2.10 GHz CPUs, running PLINK v1.9 under Ubuntu 14.04 LTS.

Performance was evaluated in a single run, in each breed for each chromosome, using ten cores. Considering 886 samples and 564,865 autosomal SNPs and 811 samples and 485,455 autosomal SNPs in HOL and NEL, respectively, the processing time was a function of the number of r² calculations, which in turn is given by:

$$NC = 0.5 \cdot x \cdot (x-1)$$

where,

NC is the number of calculations performed;

x is the number of markers.

A total of 1.60 x 10^{11} and 1.18 x 10^{11} calculations were performed for HOL and NEL, resulting in 18h26m and 11h58m of processing time, respectively. Figure 1 shows the benchmark, for each chromosome in each breed. Such calculations may be affected by sample size.

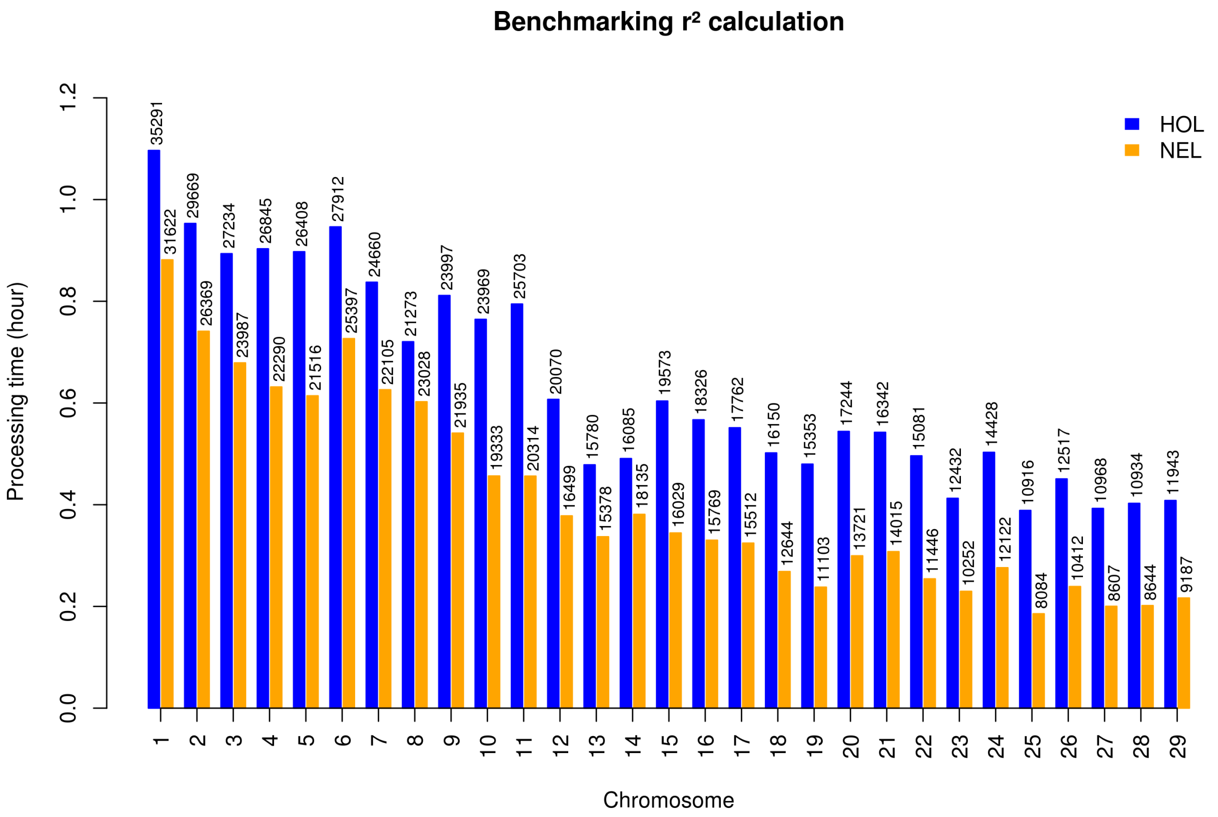

Figure 1. Histogram of the processing time to perform r² calculations. The numbers over the bars represent the amount of SNPs in the chromosome.
